# An inhibitor of ubiquitin conjugation and aggresome formation†
†Electronic supplementary information (ESI) available. See DOI: 10.1039/c5sc01351h



**DOI:** 10.1039/c5sc01351h

**Published:** 2015-06-22

**Authors:** Heeseon An, Alexander V. Statsyuk

**Affiliations:** a Department of Chemistry , Center for Molecular Innovation and Drug Discovery , Chemistry of Life Processes Institute , Northwestern University , Silverman Hall, 2145 Sheridan Road , Evanston , Illinois 60208 , USA

## Abstract

Proteasome inhibitors have revolutionized the treatment of multiple myeloma, and validated the therapeutic potential of the ubiquitin proteasome system (UPS). It is believed that in part, proteasome inhibitors elicit their therapeutic effect by inhibiting the degradation of misfolded proteins, which is proteotoxic and causes cell death. In spite of these successes, proteasome inhibitors are not effective against solid tumors, thus necessitating the need to explore alternative approaches. Furthermore, proteasome inhibitors lead to the formation of aggresomes that clear misfolded proteins *via* the autophagy–lysosome degradation pathway. Importantly, aggresome formation depends on the presence of polyubiquitin tags on misfolded proteins. We therefore hypothesized that inhibitors of ubiquitin conjugation should inhibit both degradation of misfolded proteins, and ubiquitin dependent aggresome formation, thus outlining the path forward toward more effective anticancer therapeutics. To explore the therapeutic potential of targeting the UPS to treat solid cancers, we have developed an inhibitor of ubiquitin conjugation (ABP A3) that targets ubiquitin and Nedd8 E1 enzymes, enzymes that are required to maintain the activity of the entire ubiquitin system. We have shown that ABP A3 inhibits conjugation of ubiquitin to intracellular proteins and prevents the formation of cytoprotective aggresomes in A549 lung cancer cells. Furthermore, ABP A3 induces activation of the unfolded protein response and apoptosis. Thus, similar to proteasome inhibitors MG132, bortezomib, and carfilzomib, ABP A3 can serve as a novel probe to explore the therapeutic potential of the UPS in solid and hematological malignancies.

## Abstract 2

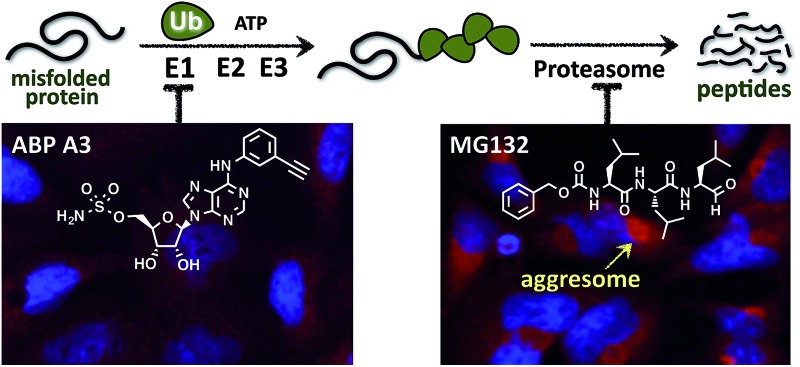
An inhibitor of ubiquitin activating E1 enzyme inhibits ubiquitin conjugation and aggresome formation.

## Introduction

The ubiquitin proteasome system (UPS) regulates intracellular protein concentration and localization, and the assembly of functional protein–protein complexes. As a consequence, the UPS controls a broad array of fundamental processes such as endocytosis, signal transduction, nuclear transport, transcription, protein quality control, and proteasomal protein degradation.^1–6^

Despite the essential function of the proteasome, it is remarkable that proteasome inhibitors bortezomib and carfilzomib show clinical efficacy in treating multiple myeloma and mantle cell lymphoma. The cytotoxicity of these agents is partly due to the accumulation of misfolded proteins in the cell, which is proteotoxic and contributes to cell death.^7^ Given that rapidly dividing cancer cells have an elevated rate of protein synthesis, they show an increased dependence on protein quality control and protein degradation.^8,9^ As a consequence, cancer cells, such as multiple myeloma cells, are more sensitive to proteasome inhibitors compared to normal cells.^7^

However, proteasome inhibitors have not found use as therapeutic agents to treat solid tumors. Furthermore, proteasome inhibitors have shown limited clinical efficacy in treating multiple myeloma.^10–12^ These limited responses are in part due to the alternative degradation of misfolded proteins *via* the aggresomal pathway.^13–17^ The aggresomal pathway clears misfolded proteins by delivering misfolded proteins to the lysosome, thereby alleviating proteotoxic stress and contributing to cell survival.

More specifically, proteasome inhibition causes the accumulation of polyubiquitinated misfolded proteins. The accumulated proteins are then recognized by histone deacetylase 6 (HDAC6) through its ubiquitin-binding domain. Subsequently, HDAC6 binds dynein motor and transports the misfolded proteins along microtubules to the microtubule-organizing center (MTOC). There, the collected misfolded proteins form a large spherical particle called an aggresome (∼10 μm^3^). Sequestration of the aggresome by autophagic vesicles, followed by fusion with a lysosome, leads to lysosomal degradation of the misfolded proteins.^18,19^ Accordingly, HDAC6 inhibitors have shown synergistic effects with bortezomib in killing patient-derived multiple myeloma cells.^14^ Similarly, disruption of aggresome formation has been effective to enhance the cytotoxic effects of bortezomib in pancreatic, breast, colon, prostate and ovarian cancer cells.^20–24^ Furthermore, bortezomib is in multiple clinical trials as a combination therapy agent, including trials for the treatment of lung cancer.

**Fig. 1:**
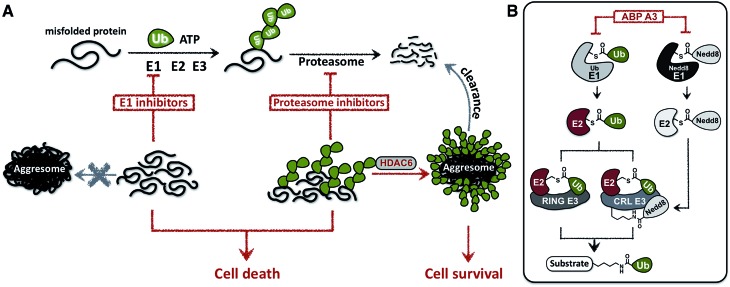
Cellular effects of E1 enzyme inhibitors *vs.* proteasome inhibitors. (A) A fraction of newly synthesized proteins misfold, followed by their polyubiquitination and proteasomal degradation. Thus, proteasome inhibitors cause the accumulation of the polyubiquitinated misfolded proteins, which induces ER stress and contributes to cell death. However, misfolded polyubiquitinated proteins can be cleared by an alternative degradation pathway, which requires ubiquitin tags on misfolded proteins and involves the formation of aggresomes. In contrast to proteasome inhibitors, E1 enzyme inhibitors would induce the accumulation of misfolded proteins, yet would not cause the formation of aggresomes due to the lack of ubiquitin tags on the misfolded proteins. (B) Dual inhibitors of ubiquitin and Nedd8 E1 enzymes inhibit ubiquitin conjugation. Ubiquitin is activated by E1 enzyme, transferred onto the catalytic cysteine of E2, and conjugated to the lysine of protein substrates in the presence of RING or Cullin-RING E3s (CRL E3s). Alternatively E2–Ub thioesters can transfer ubiquitin onto the catalytic cysteine of HECT or RBR E3s (not shown), which then conjugate the ubiquitin onto the lysine of protein substrates. CRL E3s require the covalent modification with Nedd8 to be activated. Therefore, dual inhibition of ubiquitin E1 and Nedd8 E1 would efficiently inhibit substrate ubiquitination.

In this paper we hypothesized that inhibition of the ubiquitin conjugation process by pharmacologically targeting ubiquitin-activating E1 enzyme should also cause the accumulation of misfolded proteins and induce proteotoxic stress. In contrast to proteasome inhibitors, however, E1 enzyme inhibitors should not induce the formation of aggresomes, because aggresome formation requires the presence of polyubiquitin tags on misfolded proteins (Fig. 1A).

To test this hypothesis, we developed a small molecule that inhibits the ubiquitin conjugation process by targeting two enzymes essential for the activity of entire ubiquitin conjugation system: the ubiquitin- and Nedd8-activating E1 enzymes.^25^ The developed inhibitor was designed based on the previously reported pan-E1 inhibitor, Compound I.^26^ The unique feature of our strategy was the activity-based profiling of the intracellular potency and selectivity of a panel of rationally designed inhibitor candidates.^27^ Our approach led to the eventual discovery of ABP A3, which efficiently inhibits ubiquitin and Nedd8 conjugation in cells.

Biological investigations revealed that ABP A3 induced unfolded protein response in A549 cells (non-small cell lung cancer), and apoptosis. In contrast to proteasome inhibitors, ABP A3 did not induce the formation of aggresomes in A549 cells, thus confirming our initial hypothesis. We envision that ABP A3 will serve as a useful tool to investigate the therapeutic potential of the UPS in treating solid and hematological malignancies in the future.

## Results and discussion

### 1. Strategic considerations

Due to the hierarchical organization of the E1, E2 and E3 enzymes comprising the ubiquitin system, E1 enzymes represent the most viable targets whose pharmacological suppression will inhibit ubiquitin conjugation. During the initial activation step, E1 activates the C-terminus of ubiquitin and forms a reactive Ub–E1 thioester complex.^28^ Ubiquitin is subsequently transferred to the catalytic cysteine of an E2 enzyme. The E2 then transfers the ubiquitin onto the lysine of a protein substrate in the presence of a RING/CRL E3, or onto the catalytic cysteine of a HECT/RBR E3, which then ubiquitinates a protein substrate.

Importantly, similar hierarchical organization (E1 → E2 → E3) is shared among other ubiquitin-like proteins (UBLs) such as SUMO1–3, Ufm1, ISG15, LC3, and Nedd8.^29^ Each UBL has its own set of E1, E2, and E3 enzymes. Notably, the major substrates of Nedd8 are cullin proteins, which are the essential components of the multisubunit Cullin-RING E3s (CRL E3).^30,31^ Covalent modification of cullins by Nedd8 is required for activation of CRL E3s, which ubiquitinate ∼20% of proteasomally degraded proteins (Fig. 1B).^25,32^ We therefore hypothesized that dual inhibition of ubiquitin and Nedd8 E1 enzymes would simultaneously suppress the enzymatic activity of Nedd8-dependent and Nedd8-independent E3 ligases and effectively inhibit ubiquitin conjugation (Fig. 1B). To avoid other pleiotropic effects, conjugation of other UBL proteins to their intracellular targets should not be inhibited.

It is important to note here that several non-cullin Nedd8 substrates have been identified recently by over-expressing Nedd8 in cells.^33,34^ However, it has been shown that over-expression of Nedd8 leads to its non-specific activation by the ubiquitin E1, followed by conjugation to these substrates *via* the ubiquitin system.^35–37^ Therefore, further evidence is required to validate the genuine nature of non-cullin Nedd8 substrates.^38^

### 2. Mechanistic considerations

To develop an inhibitor of ubiquitin- and Nedd8-activating E1 enzymes, we relied on our previous experience of developing the activity-based probe ABP1 for E1 enzymes.^39^ ABP1 was designed based on previously developed pan-E1 inhibitor Compound I, both of which closely mimic the structure of AMP.^26^ The unique feature of ABP1, however, is the presence of an alkyne tag that facilitates the rapid identification of intracellular targets of ABP1 and its intracellular selectivity and potency.^40^

**Fig. 2:**
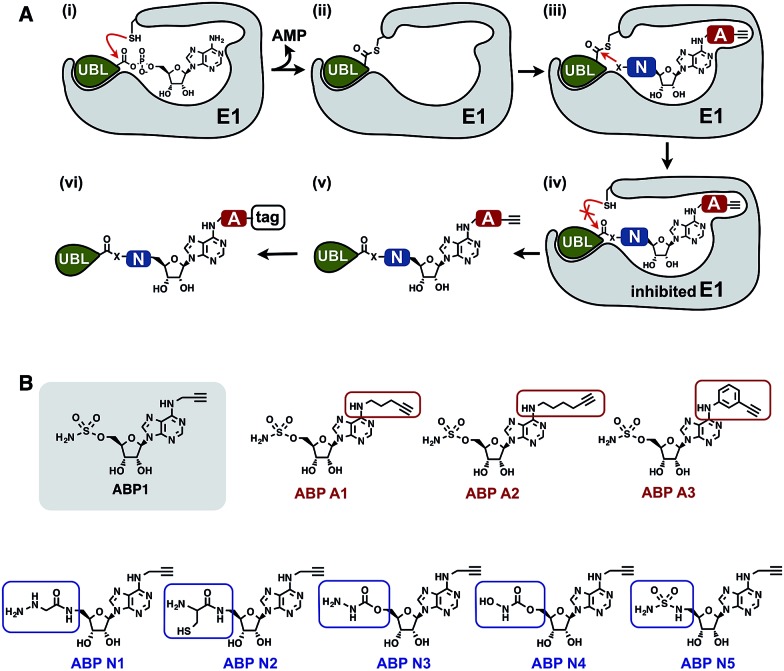
Mechanistic considerations in the design of selective inhibitors of E1 enzymes. (A) Mechanism of ABP1 analogue-mediated E1 inhibition (B) Structures of proposed ABP1 analogues.

ABP1 utilizes the conserved catalytic mechanism of E1s. First, ATP and UBL bind to E1, followed by the formation of UBL·AMP complex accompanied by the release of PP_i_ (Fig. 2A-i).^28^ Subsequently, the catalytic cysteine of the E1 enzyme forms a thioester bond with the C-terminus of UBL, releasing AMP (Fig. 2A-ii). ABP1 mimics AMP and therefore binds the AMP binding site of the Ub–E1 thioester, followed by nucleophilic attack of the sulfamate nitrogen of ABP1 onto the Ub–E1 thioester (Fig. 2A-iii). As a result, ABP1 forms a stable covalent complex with the UBL, which mimics the UBL adenylate. This UBL·ABP1 adduct binds tightly to the E1 enzyme active site and inhibits its function (Fig. 2A-iv).^41^ Based on this mechanism, we demonstrated that the ability of ABP1 to form UBL·ABP1 covalent adducts correlated with its ability to inhibit the corresponding E1s.

To design ABP1 analogues, which would selectively inhibit ubiquitin- and Nedd8-activating E1 enzymes, we first envisioned that having an alkyne tag on ABP1 analogues would facilitate the efficient profiling of intracellular potency and selectivity of inhibitors against E1 enzymes (Fig. 2B).^40^ Subsequently, we decided to explore two features of ABP1: (a) its intrinsic chemical reactivity toward UBL–E1 thioesters, and (b) its binding affinity to UBL–E1 thioesters.

To explore the chemical reactivity of ABP1 toward thioesters, we replaced the sulfamate in ABP1 with other nucleophiles. We hypothesized that different nucleophiles may exhibit different reactivities toward UBL–E1 thioesters, providing one selectivity filter for E1 enzymes. We call such ABP1 analogues ‘nucleophile analogues’.

To control binding affinity of ABP1 analogues to E1, we installed different hydrophobic groups at the N6-nitrogen position in ABP1. The different binding affinity of ABP1 analogues to UBL–E1 thioesters may cause different rates of covalent adduct formation. The hydrophobic N6-substituent should also alter the binding affinity of UBL·inhibitor adducts to E1 enzymes, affecting their inhibitory potency. This would provide another selectivity filter. We call such analogues ‘affinity analogues’. Altogether, we prepared five nucleophile analogues and three affinity analogues, all of which contain an alkyne tag (Fig. 2B).

### 3. Reactivity of nucleophile analogues toward thioesters

**Fig. 3:**
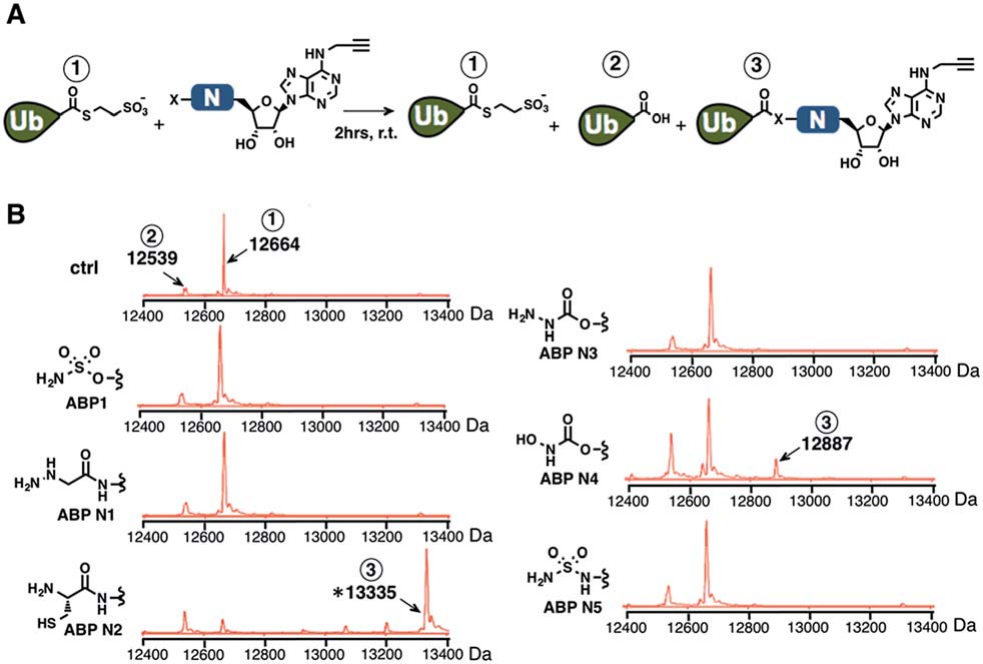
(A) Schematic description of the intrinsic reactivity test of the nucleophile analogues using pre-activated ubiquitin thioester Ub–MES. (B) Deconvoluted mass spectra of the Ub–MES (100 μM) and ABP (1 mM) reaction mixture, after 2 hours of incubation at r.t. Reaction products are labeled as follows (1) Ub–MES, (2) ubiquitin, the hydrolysis product of Ub–MES, (3) Ub·ABP. * Ub·ABP N2 formed disulfide bond with free ABP N2, which was reduced to Ub·ABP N2 in the presence of TCEP (Fig. S1†).

We prepared a series of nucleophile analogues of ABP1 that have a hydrazine (ABP N1), cysteine (ABP N2), hydrazide (ABP N3), *N*-acylhydroxylamine (ABP N4) or sulfamide (ABP N5) instead of the sulfamate moiety (Scheme S1–2,† and Fig. 2B). We first investigated the intrinsic reactivity of the nucleophile analogues toward thioesters in the absence of E1 enzyme. As a source of thioester, we used a ubiquitin thioester that has a β-mercaptoethane sulfonate thioester group at the C-terminus of ubiquitin (Ub–MES, Fig. 3A). We incubated 100 μM Ub–MES with 1 mM of each ABP1 analogue in pH 7.6 HEPES buffer for 2 hours at room temperature, followed by MS analysis of the reaction mixture.

ABP1 itself did not react with Ub–MES, suggesting that E1 enzyme is critical for the covalent labeling of ubiquitin with the sulfamate group (Fig. 3B). This could be because the E1 enzyme elevates the effective molarity between the two reactants, *i.e.* the sulfamate on ABP1 and the thioester in Ub–E1 complex. Alternatively, E1 could increase the reactivity of the sulfamate by forming hydrogen bonds with oxygens in the sulfamate, lowering the p*K*_a_ of the terminal amine.^26^ This result suggests that the sulfamate functional group should not display promiscuous thioester reactivity in cells because of its low intrinsic chemical reactivity.

Further analysis revealed that the cysteine analogue ABP N2 was the most reactive with Ub–MES, showing 90% conversion based on the ion intensities in MS spectra. ABP N2 most likely formed the covalent adduct *via* a native chemical ligation reaction. The second most reactive molecule was ABP N4, which showed 12% and 23% of Ub–MES conversion into Ub·ABP N4 and hydrolyzed ubiquitin, respectively. We suggest that the increased amount of hydrolyzed ubiquitin could be due to the hydrolysis of oxyester in the Ub·ABP N4 adduct.^42^ Taken together, our findings that ABP N2 displayed high reactivity to thioester indicate that cysteine can potentially be used to design thioester reactive probes. However, their promiscuity toward off-target thioesters needs also be considered.

### 4. Reactivity of ABP1 analogues toward Ub–E1 thioester

**Fig. 4:**
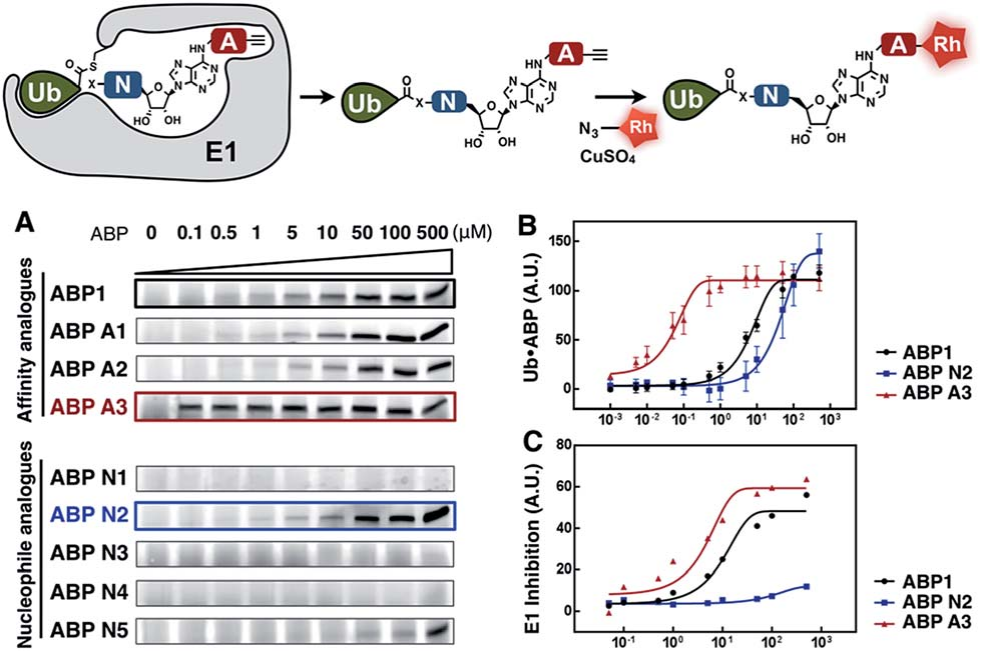
(A) Dose dependent formation of Ub·ABP covalent adducts in the presence of UBE1 (0.5 μM), ATP (50 μM) and Ub (50 μM), after 2 hours at r.t. (B) The labeling efficiency of ABP1, ABP N2 and ABP A3 was compared *via* the quantification of the Ub·ABP levels at different concentrations. Each data point is shown as a triplicate (C) The inhibition potency of protein ubiquitination by ABP1, ABP N2 and ABP A3 was plotted based on the fluorescence intensity ratio of ubiquitin-modified GFP-Sic60 to total GFP-Sic60.

Subsequently, we investigated the ability of ABP A1–3 and ABP N1–5 to label ubiquitin in the presence of UBE1 enzyme (Fig. 4). By comparing the labeling potency of affinity analogues, we expected to analyze the link between hydrophobic N6-substituents and their affinity to Ub–E1 (Fig. 4A). By comparing the nucleophile analogues, we intended to explore the combination effect of the intrinsic nucleophilicity and binding to Ub–E1 catalytic center on ubiquitin labeling (Fig. 4A). Lastly, we investigated the correlation between the ability of ABP1 analogues to form UBL·ABP adducts in the E1 binding pocket, and their ability to inhibit E1 enzyme activity (Fig. 4B and C). Such correlation would verify our approach to profile the potency and selectivity of ABP1 analogues in live cells based on alkyne tag-mediated detection of UBL·ABP covalent adduct levels.

Initially, ubiquitin and ubiquitin E1 (UBE1) were used as a model system. First, an increasing concentration of each ABP1 analogue was incubated with UBE1, Ub and ATP for two hours at r.t., followed by conjugation to rhodamine-azide under click chemistry reaction conditions. The resulting Ub·ABP adducts were quantified by in-gel fluorescence scanning.

Among the affinity analogues, ABP A3 was the most potent at labeling ubiquitin (Fig. 4A). The concentrations that reach 50% of the maximal Ub·ABP1 and Ub·ABP A3 adduct formation (*K*_½_) were 7.87 and 0.043 μM respectively, suggesting that ABP A3 is approximately 180 times more potent than ABP1 in labeling ubiquitin (Fig. 4B and S2†). Among nucleophile analogues, ABP N2 showed the strongest ability to covalently label ubiquitin, with a *K*_½_ of 33.3 μM (Fig. 4B). We reasoned that some amount of Ub·ABP N2 could be formed through the direct nucleophilic attack of ABP N2 onto Ub–E1 thioester, not involving binding to the E1 enzyme due to the strong intrinsic reactivity of the cysteine. Indeed, a control molecule cABP N2 that has a cysteine and alkyne, yet lacking the adenosine moiety also labeled ubiquitin (Fig. S3†).

It is worth noting that ABP N5, which has very high structural similarity to ABP1, was much less potent at labeling ubiquitin when compared to ABP1 (Fig. 4A and S4†). This result highlights the importance of the sulfamate, the closest bioisostere of phosphate, in AMP-mimicking inhibitors of E1 enzymes.

Finally, we tested the correlation between the ability of inhibitors to form Ub·ABP adduct and their E1 enzyme inhibition using a protein ubiquitination assay. The most reactive three inhibitors, ABP1, ABP N2, and ABP A3 were incubated with UBE1 (E1), UbcH5a (E2), Rsp5 (E3), ubiquitin, ATP, and the model GFP-Sic60 substrate (Fig. S5†). The fluorescence intensity ratio of ubiquitin-modified GFP-Sic60 to total GFP-Sic60 confirmed that strong labeling efficiency of ubiquitin could be correlated with strong inhibition of UBE1, revealing ABP A3 as the most potent inhibitor of E1 (Fig. 4C).

Taken together, our activity-based profiling of UBL·ABP levels using click chemistry can be used for the efficient screening of ABP1 analogues for E1 enzyme inhibition. After validating our detection approach *in vitro*, we moved forward to investigate the selectivity and potency of ABP1 analogues in live cells.

### 5. Discovery of ABP A3 as a dual inhibitor of ubiquitin and Nedd8 conjugation pathways

The unique feature of our approach is the ability to evaluate the intracellular potency and selectivity of ABP1 analogues inside intact cells using click chemistry. This property is important because *in vitro* assays do not exactly recapitulate an intracellular environment. Accordingly, many chemical probes such as proteasome and kinase inhibitors have shown differences in their *in vitro* and intracellular potencies and selectivities.^43,44^ We therefore hypothesized that the direct analysis of UBL·ABP formation in live cells would provide the most direct and relevant readout about inhibitor potency and selectivity.

**Fig. 5:**
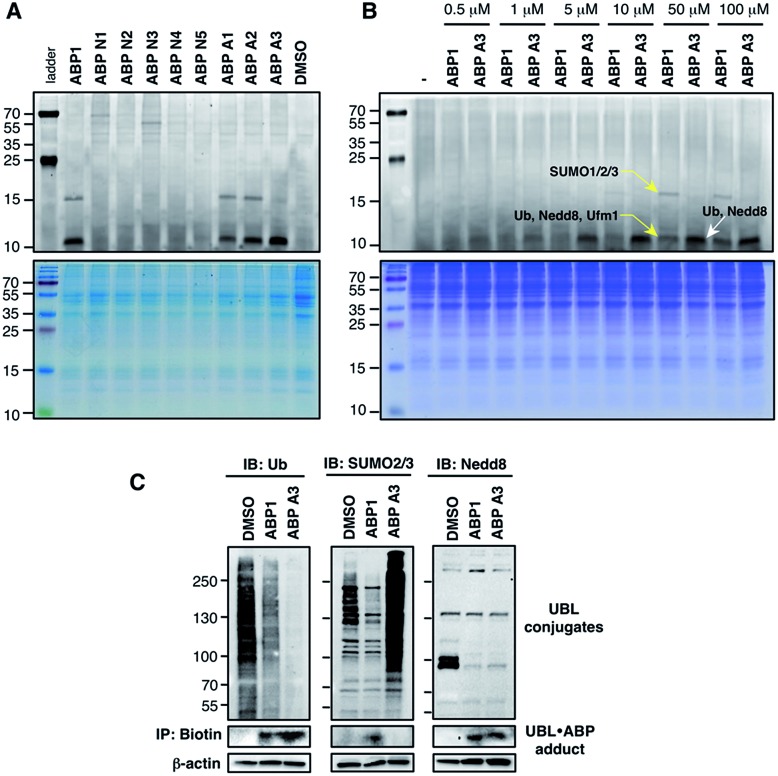
Labeling of proteins by ABPs in live A549 cells (A) A549 cells were treated with each ABP (100 μM) for 1 hour, followed by cell lysis, conjugation to fluorescent dye *via* copper-mediated [3 + 2] cycloaddition reaction, and in-gel fluorescence scanning. (B) Selectivity comparison between ABP1 and ABP A3 at different concentrations. (C) Correlation between UBL·ABP covalent adduct formation and the inhibition of the corresponding UBL conjugation pathways.

A549 cells were treated with 100 μM of each ABP, followed by cell lysis and conjugation to rhodamine dye using click chemistry. Subsequent in-gel fluorescence scanning showed that ABP1, ABP A1 and ABP A2 produced two fluorescent bands with MW ∼15 and ∼10 kDa, similar to our earlier reports (Fig. 5A). Interestingly, ABP A1 and ABP A2 showed similar labeling pattern to ABP1 in spite of the larger hydrophobic substituent at N6 of the adenosine moiety (Fig. 5A). However, treatment of A549 cells with ABP A3 produced only one fluorescent band at ∼10 kDa region, which was a surprising discovery (Fig. 5A and B). To investigate the identity of the covalently labeled proteins, we conjugated biotin to the UBL·ABP adducts using click chemistry and purified the biotinylated proteins using streptavidin beads, followed by on-bead tryptic digestion and MS analysis (Fig. S6†). We found that ABP A3 selectively labeled ubiquitin and Nedd8 proteins (∼10 kDa) with high efficiency. In contrast, ABP1 showed labeling of ubiquitin, Nedd8, SUMO1/2/3 and Ufm1 (∼10 kDa and ∼15 kDa) proteins with little selectivity.

It is important to note that both ABP1 and ABP A3 were equally effective at labeling SUMO1 *in vitro*, but ABP A3 was much less effective at labeling SUMO in cells (Fig. S7A,† and Fig. 5B). Also, labeling of proteins with ABPs in cell lysates showed significantly different labeling pattern from that of live cells, highlighting the importance of screening chemical probes in live cells (Fig. S8†). Altogether, the results emphasize the advantage of our activity-based profiling methods in evaluating the intracellular selectivity of E1 inhibitors with high accuracy.

Another interesting aspect of our studies is the fact that cysteine-containing ABP N2, which was the most chemically reactive probe to thioesters *in vitro*, did not show any labeling of proteins in intact cells. It is possible that ABP N2 did not cross the cell membrane, possibly due to chemical interaction with surface cysteines of membrane proteins (Compare ABP N2 in Fig. 5A and S8†).^45^

To further confirm the labeling selectivity of ABP A3, and to correlate it with the inhibition of the corresponding UBL conjugation, A549 cells were treated with ABP A3 or ABP1 followed by cell lysis, click reaction with biotin-azide, and purification of biotinylated proteins with streptavidin beads. The isolated UBL·ABP adducts as well as total cell extracts were immunoblotted with anti-Ub, SUMO2/3 and Nedd8 antibodies. The results showed that ABP A3 covalently labeled intracellular ubiquitin and Nedd8 very efficiently but not SUMO2/3. Accordingly, it inhibited protein ubiquitination and neddylation (Fig. 5C). On the other hand, ABP1 labeled all three UBLs tested, and showed decrease in Ub, SUMO2/3 and Nedd8 conjugation levels. Paradoxically, ABP A3 did not inhibit SUMO2/3 conjugation, but rather caused a dramatic increase in the SUMO2/3 conjugate levels in A549 cells (Fig. 5C). Given that protein SUMOylation is activated in response to cellular stresses that affects protein quality control, we hypothesized that the increase in SUMOylation is due to the accumulation of misfolded proteins caused by ABP A3 treatment.^46,47^ We will further address this in Section 8.

Altogether, our results indicate that the covalent labeling of UBL proteins with ABP1 analogues correlates with their ability to inhibit UBL signaling in cells. The key to this discovery was the activity-based proteomics that identified ABP1 as a pan-inhibitor of UBL pathways, while ABP A3 was identified as a dual inhibitor of ubiquitin and Nedd8 pathways.

### 6. ABP A3 inhibits ubiquitin and Nedd8 conjugation

**Fig. 6:**
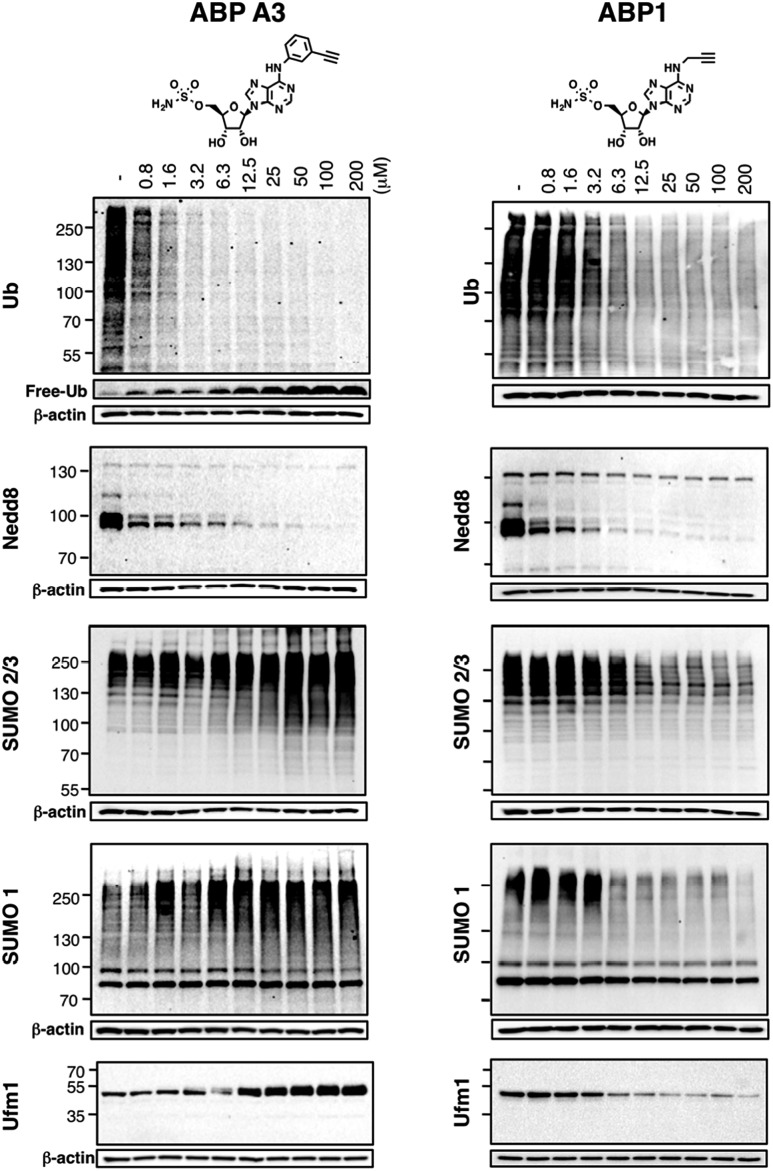
Intracellular selectivity of ABP A3 on UBL conjugation. A549 cells were treated with different concentrations of ABP A3 or ABP1 for 16 hours, lysed, and immunoblotted using anti-Ub, Nedd8, SUMO1, SUMO2/3 and Ufm1 antibodies. ABP A3 inhibited protein ubiquitination and neddylation, while not inhibiting but rather activating SUMOylation and ufmylation. In contrast, ABP1 inhibited all tested UBL conjugation systems.

Using the in-gel fluorescence and MS methods, we demonstrated that ABP A3 selectively labeled ubiquitin and Nedd8 in cells. Accordingly, ABP A3 inhibited ubiquitin and Nedd8 conjugation to intracellular substrates. To further confirm the observed selectivity, we investigated the effect of ABP A3 on conjugation levels of other UBLs. Specifically, we investigated the effects of ABP1 and ABP3 on ubiquitin, Nedd8, SUMO1–3, and Ufm1, because we identified these proteins as ABP1 targets in our MS experiments.^48^ The effect of ABP1 and ABP A3 on ISG15 (interferon stimulated gene 15) conjugation was also examined. A549 cells were incubated with different concentrations of ABP A3 or ABP1, and lysed at different time points. The level of UBL conjugates was subsequently examined using anti-Ub, Nedd8, SUMO1, SUMO2/3, Ufm1, and ISG15 antibodies (Fig. 6 and S9–S12†). As we expected, time- and ABP A3 dose-dependent decreases in ubiquitination and neddylation levels were observed. Concomitant accumulation of free ubiquitin was also detected (Fig. 6 and S11B†).

Consistent with our previous observation, ABP A3 treatment elevated SUMO1 and SUMO2/3 conjugate levels robustly and in a dose dependent manner. Similarly, we detected a gradual increase in Ufm1 conjugate levels upon ABP A3 treatment (Fig. 6). Ufm1 is a relatively new protein, and little is known about its biology; however, recent studies have found that ufmylation is induced by the unfolded protein response (UPR).^49^ Taken together, the enhancement in protein SUMOylation and ufmylation suggest that ABP A3 may cause the accumulation of misfolded proteins.

We also investigated whether ABP A3 inhibits ISG15 conjugation. Since ISG15 is not constitutively expressed in A549 cells, we induced ISG15 by treating the cells with IFN-β. Subsequent to ISG15 induction, cells were treated with different concentrations of ABP A3 and lysed at different time points. The assay showed that ABP A3 weakly inhibited ISG15 conjugation, revealing a decrease in ISG15 conjugates at 25 μM after 8 hour incubation (Fig. S11†). Since ISG15 is not normally expressed in A549 cells, we thought that the weak off-target inhibition of ISG15 would not interfere with further studies.

Unlike ABP A3, however, A549 cells treated with ABP1 showed global decrease of Ub, Nedd8, SUMO1, SUMO2/3, and Ufm1 conjugates, a result that was supported by our initial activity-based profiling experiments (Fig. 6, S10 and S12†). The most notable and unexpected difference between ABP A3 and ABP1 was the robust (up to 200 μM) increase in protein SUMOylation in ABP A3 treated cells, in contrast to a decrease in SUMOylation in ABP1 treated cells. Compound I, a previously reported pan-inhibitor of E1s, showed biphasic behavior with an increase in SUMOylated proteins at 10 μM, and then significant decrease at 50 μM (Fig. S10 and S13†). Finally, the sulfamide-containing ABP N5 did not label any UBLs in A549 cells, nor did it induce any change in Ub, Nedd8, or SUMO2/3 conjugate levels (Fig. S14†). This result further confirmed our notion that covalent adduct formation of ABPs with UBLs matches well with their ability to inhibit the corresponding UBL signaling pathways in cells.

### 7. The role of the ubiquitin system in regulating cell viability

**Fig. 7:**
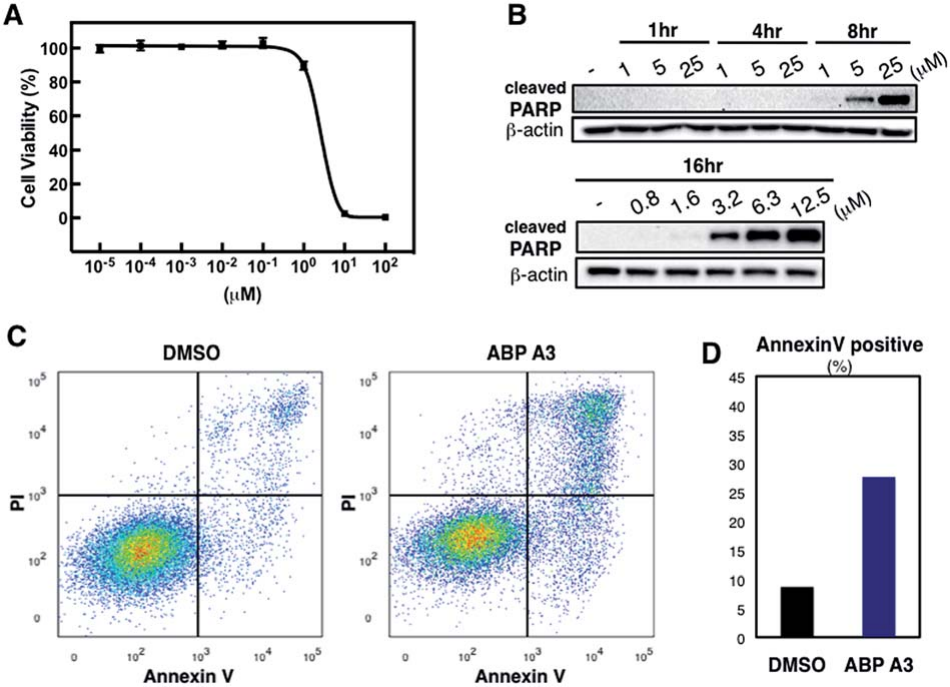
Effect of ABP A3 on cell viability and apoptosis (A) A549 cells were treated with different concentrations of ABP A3 for 48 hours. Cell viability was monitored using CellTiter-Glo and analyzed with Prism software. Each data point is an average of six replicates. (B) ABP A3 treatment of A549 cells induced PARP cleavage and apoptosis (C) Flow cytometry analysis of ABP A3 treated cells (D) Total percentage of Annexin V-positive cells was plotted based on the flow cytometry data in (C).

Having developed an inhibitor of the ubiquitin system, we decided to investigate the effect of ABP A3 on cell viability. A549 cells were treated with increasing concentrations of ABP A3 or DMSO for 48 hours, followed by cell viability assay using CellTiter-Glo (Fig. 7A). The calculated IC_50_ value was 2.5 μM.

To investigate the ABP A3-induced mechanism of cell death, we tested whether the decreased cell viability of A549 cells was associated with the induction of apoptosis. To do so, the accumulation of cleaved PARP (poly ADP ribose polymerase, cleaved by caspase-3) was analyzed as a marker of apoptosis. These experiments revealed that ABP A3 induced apoptosis in A549 cells in a time- and concentration-dependent manner (Fig. 7B). Initial appearance of the cleaved PARP was detected when A549 cells were treated with 3.2 μM ABP A3 for 16 hours, which was very close to the IC_50_ in our cell viability assay (2.5 μM).

To further confirm the apoptotic cell death, we conducted flow cytometry experiments, in which we stained A549 cells with fluorophore-labeled Annexin V that detects the amount of phosphatidyl serine displayed on the surface of apoptotic cells. Propidium iodide (PI), a DNA intercalator, was used to distinguish between dead and live cells, since PI is not cell membrane permeable. A549 cells were treated with 2.5 μM of ABP A3 or DMSO for 24 hours, and the number of apoptotic cells was counted (Fig. 7C and D). As we expected, an increased number of Annexin V-positive cells upon ABP A3 treatment was observed, confirming the induction of apoptosis.

### 8. ABP A3 activates unfolded protein response

Since we hypothesized that ABP A3 treatment should lead to accumulation of misfolded proteins in A549 cells, we investigated if the observed induction of apoptosis by ABP A3 was due to the induction of the unfolded protein response (UPR). We first analyzed the effect of ABP A3 on BiP.^50^

**Fig. 8:**
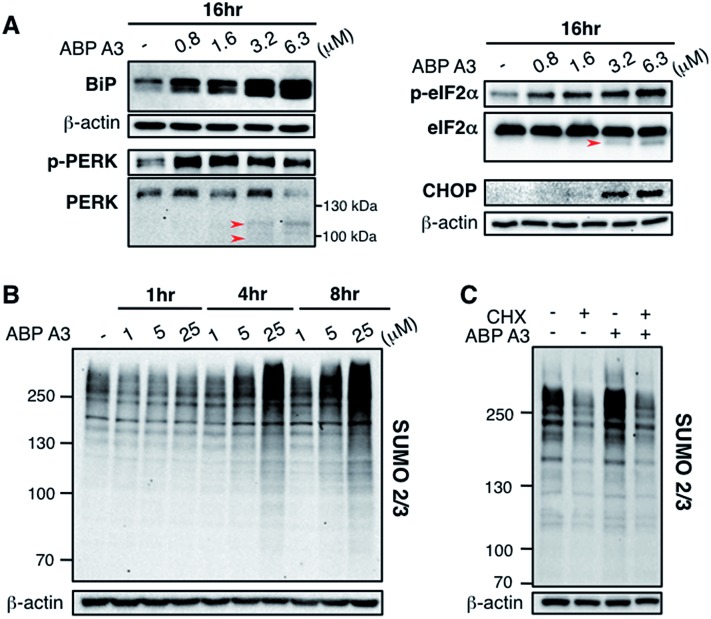
Effect of ABP A3 on protein quality control in A549 cells (A) A549 cells treated with ABP A3 for 16 hours were immunoblotted with antibodies for UPR markers BiP, PERK, eIF2α, and the apoptosis marker CHOP. (B) Immunoblotting with anti-SUMO2/3 antibody revealed dramatic accumulation of SUMO conjugates in ABP A3-treated A549 cells. (C) A549 cells were treated with or without cycloheximide (30 μg ml^–1^) for 1 hour followed by the treatment with ABP A3 (10 μM) for an additional 3 hours. Inhibition of protein synthesis by cycloheximide restored ABP A3-induced SUMO conjugation to its basal levels.

BiP (Binding inmmunoglobulin protein) is an HSP70 chaperone, which is located at the ER lumen and binds misfolded proteins for subsequent refolding.^51^ An increase in misfolded proteins reduces the amount of free BiP, which in turn activates BiP synthesis.^52^ Therefore, an increase in the total amount of BiP indicates an increase in misfolded protein levels. We found that ABP A3-treated cells showed a significant, dose-dependent increase in BiP levels (Fig. 8A).

To further investigate the effect of ABP A3 on the UPR, we then focused on PERK–eIF2α signaling arm of the UPR.^50^ PERK is an ER transmembrane kinase, which is activated *via* oligomerization and autophosphorylation upon the accumulation of misfolded proteins in the ER. Subsequently, autophosphorylated PERK phosphorylates eIF2α (eukaryotic translation initiation factor), which inhibits protein synthesis, and protects cells from further influx of misfolded proteins. Upon prolonged ER stress, however, PERK–eIF2α signaling switches its role from being cytoprotective to pro-apoptotic.^50^ In this case, PERK–eIF2α signaling triggers cell death partly by inducing CHOP (C/EBP homologous protein), which upregulates the transcription of genes involved in apoptosis.^53^

We observed that both p-PERK and p-eIF2α levels increased in the presence of ABP A3, while the total level of PERK and eIF2α were not affected (Fig. 8A). Furthermore, the induction of apoptotic transcription factor CHOP was observed at 3.2 μM of ABP A3, the concentration at which cleaved PARP was observed (Fig. 7B). Interestingly, cleaved form of PERK and eIF2α also appeared at the same 3.2 μM concentration of ABP A3, which may explain the disappearance of PERK band at 6.3 μM ABP A3 (Fig. 8A, red arrows). Furthermore, eIF2α cleavage is known to be dependent on caspase-3 activity, which is activated upon apoptosis.^54,55^ Taken together, our results suggest that ABP A3-induced apoptosis in A549 cells was in part due to the PERK–eIF2α arm of UPR.

In addition, it is believed that SUMOylation controls protein quality by enhancing the solubility of misfolded proteins, or by recruiting SUMO-targeted ubiquitin ligases.^56–58^ Notably, cells treated with proteasome inhibitors showed elevated levels of SUMO2/3 conjugates mainly on the newly synthesized proteins.^47,59^ Similarly, we observed an increase in SUMO2/3 conjugates in ABP A3-treated cells accompanied by the decrease of free SUMO2/3 (Fig. 8B and S15B†). Based on the previous findings, we hypothesized that the SUMOylation targets during ABP A3 treatment could also be newly synthesized proteins. To test this hypothesis, we co-treated A549 cells with ABP A3 and cycloheximide (CHX, an inhibitor of protein synthesis), and monitored the accumulation of SUMOylated proteins (Fig. 8C). Indeed, the accumulation of SUMO2/3 conjugates induced by ABP A3 was restored to basal levels when protein synthesis was blocked. The accompanying increase of free SUMO2/3 proved that the decreased SUMOylation level was not due to decreased SUMO2/3 synthesis (Fig. S16†). These results suggest that similar to proteasome inhibitors, newly synthesized proteins are SUMOylated in ABP A3-treated cells, probably as a result of stress response.

### 9. ABP A3 treatment does not lead to aggresome formation

Given the critical role of protein ubiquitination in aggresome formation and degradation,^18,60^ we were interested in interrogating the effect of ABP A3 on aggresome formation and autophagy. We hypothesized that misfolded proteins would lack polyubiquitin tags, and therefore would not be recognized and transported by HDAC6. Therefore, aggresome formation would not be observed.

**Fig. 9:**
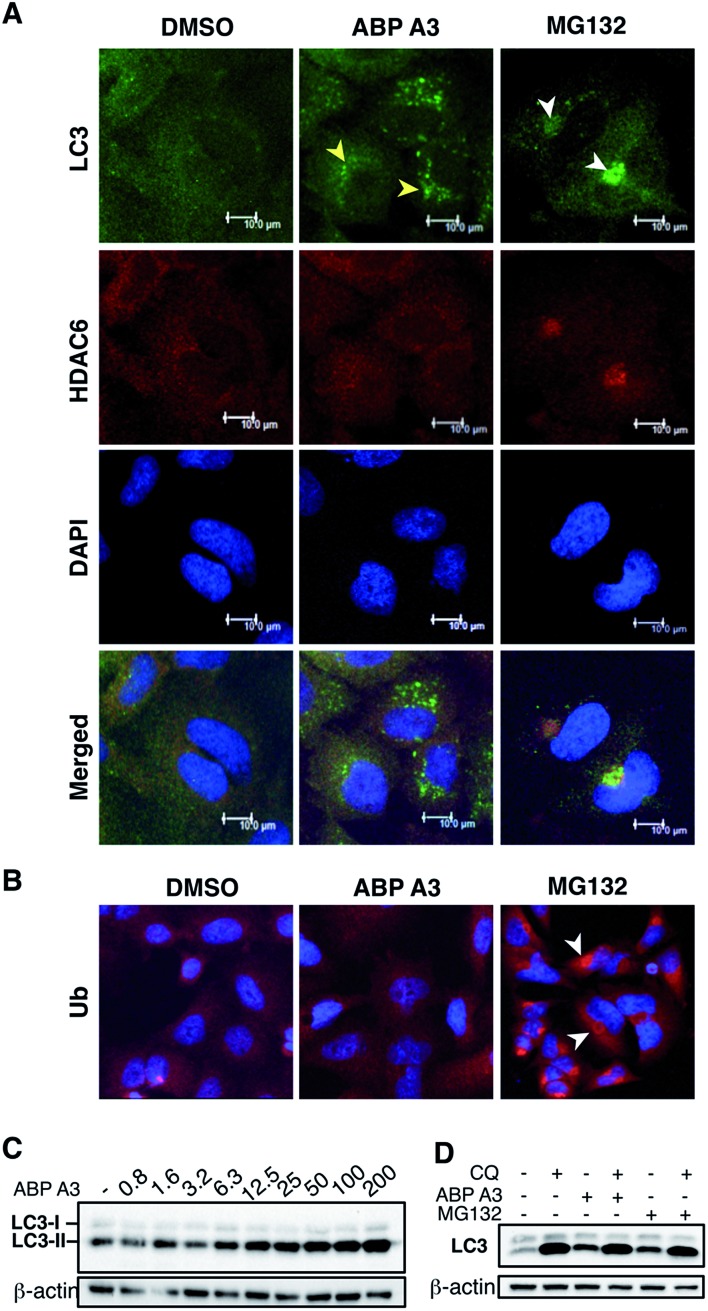
Effect of ABP A3 on the aggresome-autophagy system in A549 cells. (A) A549 cells were treated with DMSO (0.025%), ABP A3 (2.5 μM) or MG132 (5 μM) for 24 hours, and immunostained with anti-LC3 and HDAC6 antibodies, and DAPI. Subsequent confocal images demonstrated the appearance of small autophagic puncta in ABP A3 treated cells (yellow arrows) and large aggresomes in MG132 treated cells (white arrows). (B) Cells prepared as in (A) were immunostained with ubiquitin antibody. (C) Immunoblotting of A549 cells treated with ABP A3 for 16 hours using anti-LC3 antibody showed accumulation of the lipidated form of LC3 (LC3-II) (D) A549 cells were treated with ABP A3 (5 μM) or MG132 (5 μM) in the presence or absence of chloroquine (CQ, 10 μM), followed by immunoblotting with LC3 antibodies. Increased amount of LC3-II upon ABP A3 + CQ treatment suggests the active autophagosome turnover in ABP A3 and MG132 treated cells.

To test our hypothesis, we incubated A549 cells with ABP A3, MG132 (proteasome inhibitor) or DMSO. The immunostaining of LC3, which is a component of autophagosomes, and HDAC6 showed that MG132 induced the formation of large aggresomes localized at the juxtanuclear region of cells (Fig. 9A). However, ABP A3 did not induce the formation of large aggresomes, as judged by the HDAC6, LC3, and ubiquitin staining.

Instead, several small autophagic puncta scattered throughout the cytoplasm were observed. Immunostaining with ubiquitin antibody also showed the ubiquitin-enriched aggresomes in MG132-treated cells, but no discernable accumulation of ubiquitin-containing aggregates was observed in DMSO or ABP A3 treated cells (Fig. 9B).

Since ABP A3 induced the formation of small autophagic puncta, we asked if ABP A3 could still activate autophagy, which is an alternative protein and organelle degradation pathway. Therefore, we monitored the steady state level of autophagosomes using LC3 western blotting (Fig. 9C). LC3 (LC3-I) is a ubiquitin-like protein that is covalently conjugated to phosphatidylethanolamine (PE) through the Atg7 (E1)-Atg3 (E2)-Atg5/Atg12/Atg16L (E3) system.^61^ LC3-modified PE (LC3-II) is brought to the early autophagic vesicle and assists its elongation.^5,62^ We found that ABP A3 induced an increase of LC3-II levels in a dose-dependent manner.

To clarify whether the accumulation of lipidated LC3-II was due to an increase in conjugation of LC3 to PE, or a decrease in lysosomal degradation of LC3-II, we decided to monitor the turnover of LC3-II in the presence and absence of lysosomal inhibitor chloroquine (CQ) (Fig. 9D). When A549 cells were treated with ABP A3 in the presence of CQ, further accumulation of LC3-II was observed, indicating that lysosomal degradation is still active in the presence of ABP A3. Therefore, the observed increase in LC3-II could be due to the increased conjugation of LC3 to PE. Similarly, MG132-treated cells showed an accumulation of LC3-II, suggesting that proteasome inhibition activates autophagy.^23^

Based on our observations, it is tempting to conclude that ABP A3 activates autophagy similar to proteasome inhibitors as an alternative degradation pathway, yet ABP A3 does not induce aggresome formation. The mechanism of autophagic puncta formation in ABP A3 treated cells and whether this formation is cytoprotective or pro-apoptotic needs further investigation. However, an increase in LC3-II upon ABP A3 treatment suggests that ABP A3 does not inhibit LC3 conjugation, further highlighting the selectivity of ABP A3.

## Conclusion

Proteasome inhibitors bortezomib and carfilzomib are clinically used to treat multiple myeloma; however these agents are not effective against solid tumors. Therefore, new approaches to target UPS are urgently needed.

To this end, inhibitors of ubiquitin activating E1 enzyme hold substantial promise as therapeutic agents. Pharmacological inhibition of E1 enzyme should inhibit ubiquitin conjugation and as a result, similar to proteasome inhibitors, inhibit protein degradation. However, in contrast to proteasome inhibitors, E1 enzyme inhibitors should not lead to the formation of aggresomes, which are known to limit the therapeutic efficacy of bortezomib and carfilzomib in both hematological malignancies and solid tumors.^10–17,20–24^ Specifically, this paper reports the discovery of ABP A3, a dual inhibitor of the ubiquitin- and Nedd8-activating E1 enzymes, which inhibits both Nedd8-dependent and Nedd8-independent ubiquitin conjugation and protein degradation. We then used these probes to investigate the therapeutic potential of the UPS system to treat solid tumors using A549 cell line (non-small cell lung cancer) as a model system.

Initially, we had to discover the ABP A3 inhibitor, because compound I was reported as a pan-E1 inhibitor with little selectivity,^26^ and Pyr41, an inhibitor of the ubiquitin E1 enzyme, is also known to inhibit DUBs.^63^ Furthermore, Pyr41 did not inhibit the activity of UBE1 in A549 cells based on our previous results.^39^ The key to our discovery was the use of activity-based proteomics. The developed ABP A3 induces selective decrease of ubiquitin and Nedd8 conjugates in A549 cells, but not SUMO1-3, Ufm1, ISG15, or LC3 conjugates. In contrast, the original probe ABP1 was not selective in cell-based assays. Furthermore, the intracellular selectivity of ABP1 and ABP A3 correlated very well with their ability to covalently label corresponding UBL proteins, highlighting the power of activity-based proteomics in profiling the intracellular selectivity of chemical probes.

Following the inhibitor discovery, we investigated the pharmacological properties of ABP A3. When tested in A549 cells, ABP A3 displayed 2.5 μM IC_50_ values in cell viability assays. The detection of cleaved PARP and Annexin V-positive cells revealed that ABP A3 induced apoptosis in A549 cells.

Since a fraction of freshly synthesized proteins misfold and are degraded by the ubiquitin proteasome system, we hypothesized that ABP A3 should cause accumulation of misfolded proteins, which can contribute to the observed apoptosis *via* the unfolded protein response (UPR). Furthermore, the increase in SUMOylation and ufmylation that we observed upon ABP A3 treatment are associated with protein quality control stress.^46,47,49^ We therefore investigated whether ABP A3 induced the UPR.

First, we observed a time- and dose-dependent increase in the levels of the chaperone BiP upon ABP A3 treatment, which indicated ER stress. Accumulation of misfolded proteins in the ER activates three major sensors of unfolded proteins: IRE1, ATF6 and PERK.^50^ We have shown that the treatment of A549 cells with ABP A3 induces phosphorylation of kinase PERK and its downstream target eIF2α, both of which are major hallmarks of the UPR. Phosphorylation of eIF2α leads to inhibition of mRNA translation, thereby reducing ER stress. Under increased ER stress, however, PERK activation induces transcription factor CHOP, which controls genes involved in apoptosis. Remarkably, we observed the induction of CHOP at 3.2 μM ABP A3 in A549 cells (but not at <1 μM), a concentration at which cleaved PARP was observed. This indicates that UPR signaling contributes to apoptosis in ABP A3-treated A549 cells. The overall results suggest that at low concentration of ABP A3, cells are coping with the accumulation of misfolded proteins by activating UPR. At higher concentrations of ABP A3, however, cells can no longer cope with the stress and the UPR activates apoptosis.

Finally, we tested the hypothesis that ABP A3 will not cause aggresome formation in A549 cells due to the lack of ubiquitin tags on the accumulated proteins. As we expected, ABP A3 treatment did not cause aggresome formation in A549 cells as judged by the lack of HDAC6, ubiquitin, and LC3 staining, while proteasome inhibitor MG132 did. Interestingly, ABP A3 still induced the formation of autophagic puncta and the lysosomal degradation process was still active. Furthermore, since ABP A3 does not inhibit LC3 conjugation to PE, this indicates that LC3 E1 (ATG7) is active during ABP A3 treatment.

Taken together, our results suggest that ABP A3 induces accumulation of misfolded proteins in cells without the formation of HDAC6, LC3, and ubiquitin-enriched aggresomes, leading to ER stress and apoptosis. However, we cannot rule out the possibility that ABP A3 may prevent proteasomal degradation of cell cycle inhibitors and tumor suppressor proteins, leading to cell cycle arrest and apoptosis.^64^ Indeed, upon ABP A3 treatment, we observed the upregulation of tumor suppressor p53 and cyclin dependent kinase inhibitor p21 (Fig. S17A†). Accordingly, we observed a G_2_M cell cycle arrest in asynchronized A549 cells upon ABP A3 treatment (Fig. S17B and C†). These last results are in agreement with previous observations for mammalian cells harboring temperature-sensitive UBA1 alleles that also underwent cell cycle arrest at G_2_M phase at non-permissive temperatures.^65,66^

Importantly, a selective inhibitor of the ubiquitin E1, MLN7243, is in phase I clinical trials to treat solid tumors, yet the structure of MLN7243 is not publicly available. Thus, we envision that in the future, ABP A3 will serve as a useful pharmacological probe to further explore the therapeutic potential of the ubiquitin system to treat solid tumors as well as hematological malignancies.

We envision that ABP A3, similar to proteasome inhibitor MG132, can be widely used for basic and translational research purposes to investigate the role of the UPS in eukaryotic biology and medicine. Further investigations of the pharmacological properties of ABP A3 *in vitro* and *in vivo* will be reported in the near future.
