# Supplementary material for: An inhibitor of ubiquitin conjugation and aggresome formation
Source: Chem Sci. 2015 Jun 22;6(9):5235–45. doi: 10.1039/c5sc01351h (PMC5500945; doi:10.1039/c5sc01351h)
Supplement: Supplementary file 1 [file SC-006-C5SC01351H-s001.pdf]

Electronic Supplementary Material (ESI) for Chemical Science.
This journal is © The Royal Society of Chemistry 2015

<Supporting Information>

An Inhibitor of Ubiquitin Conjugation and Agressome Formation

Heeseon An and Alexander V. Statsyuk*

Department of Chemistry, Center for Molecular Innovation and Drug Discovery, Chemistry of Life Processes Institute, Northwestern University, Silverman Hall, 2145 Sheridan Road, Evanston, Illinois 60208

Table of Contents

I.	Supporting data	S2
II.	Synthesis	S19
III.	Bio-assay protocols	S40
IV.	Spectral data of ABPs	S48
V.	References	S56

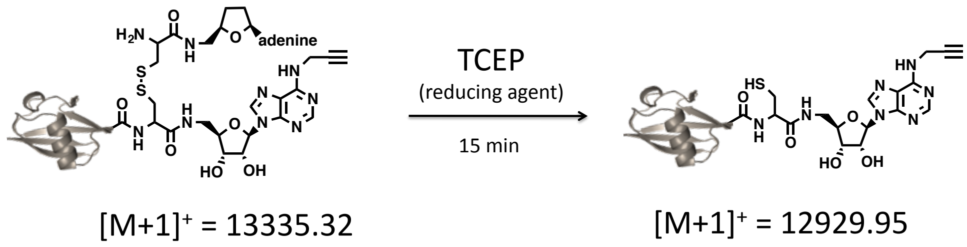

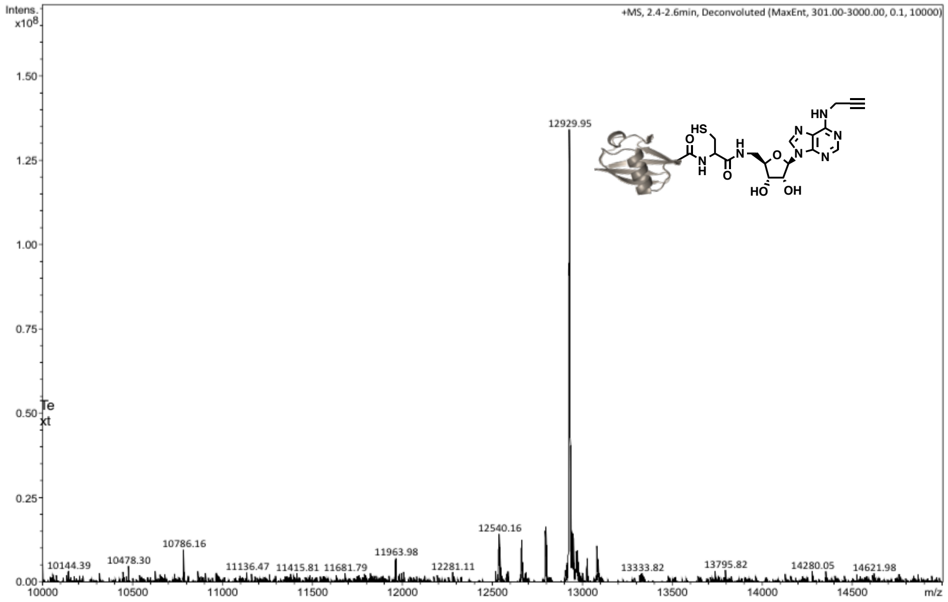

Figure S1. MS analysis of Ub•ABP N2 after reduction. Ub•**ABP N2** formed dithiobond with a free **ABP N2** at the given condition in Figure 3. The reduction of this dithiobond was observed when incubated with 1 mM TCEP for 15 min.

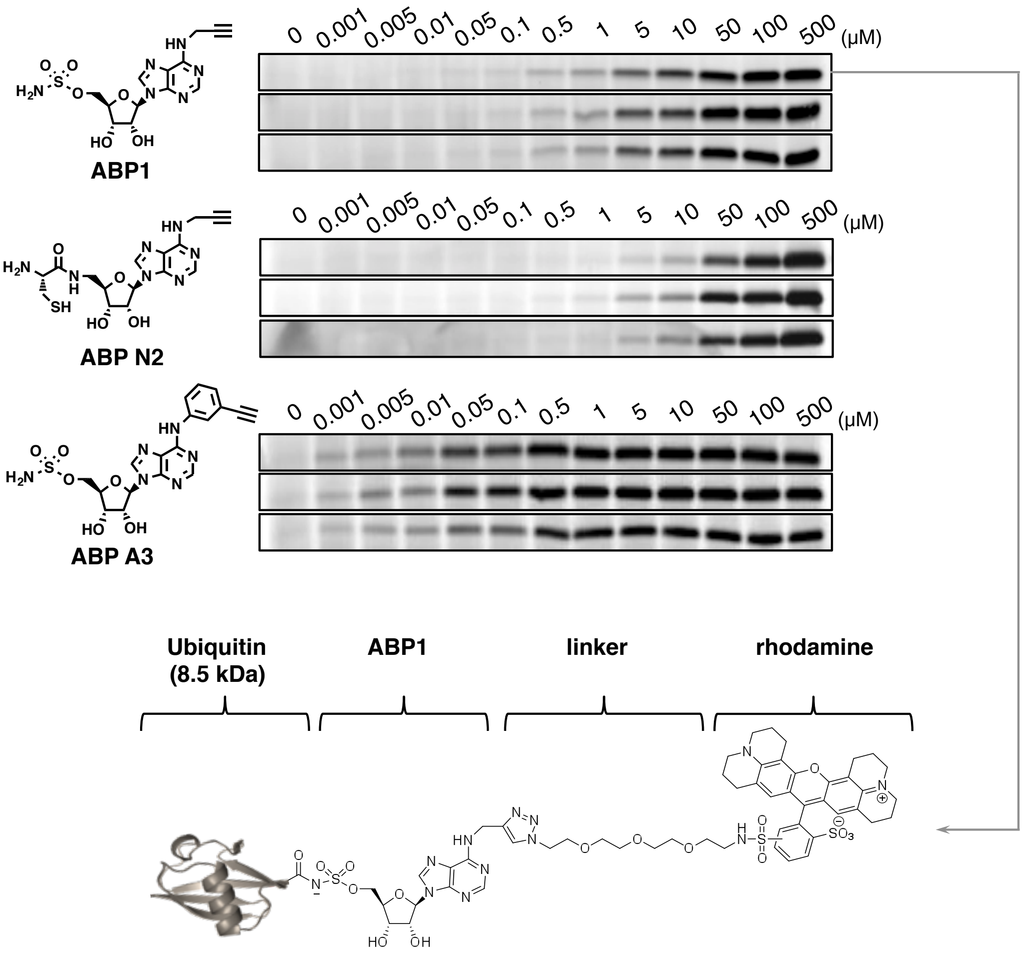

Figure S2. In-gel fluorescence image of the three independent experiments in figure 4B. UBE1 (0.5 μM), ubiquitin (50 μM) and ATP (50 μM) were incubated in the presence of different concentrations of ABP1, ABP N2, and ABP A3 for 2 hours at r.t. Covalent ubiquitin•ABP conjugates were visualized and quantified using the standard protocol.

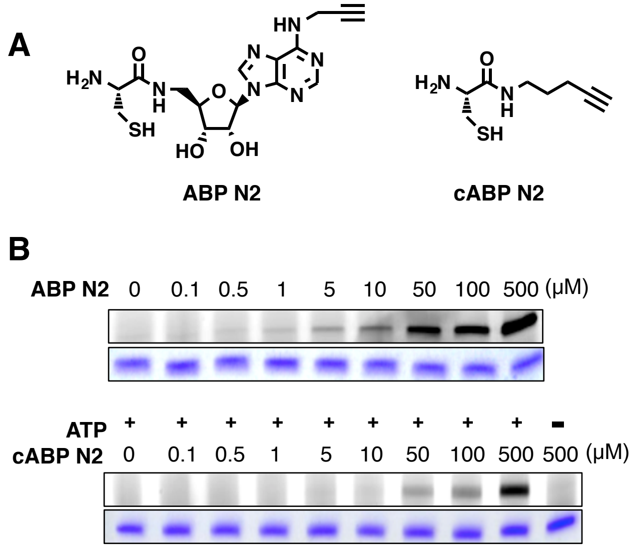

Figure S3. Cysteines can label ubiquitin by attacking the E1~Ub thioester without binding to the E1 binding pocket (A) Molecular structure of ABP N2 and its control molecule cABP N2. (B) UBE1 (0.5 µM), ubiquitin (50 µM) and ATP (50 µM) were incubated in the presence of different concentrations of ABP N2 or cABP N2 for 2 hours at r.t. The covalent conjugates were visualized and quantified using the standard protocol.

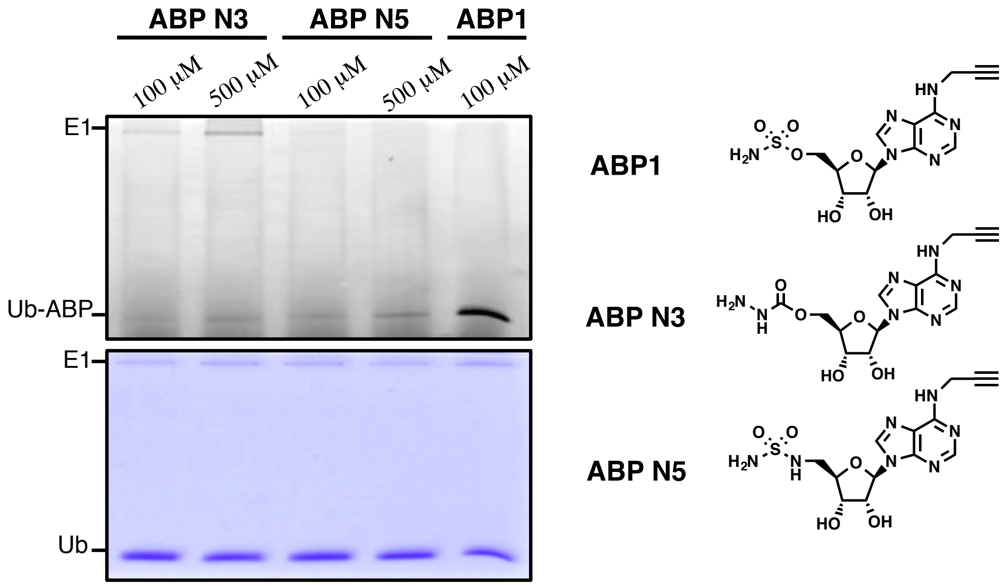

Figure S4. ABP N5 shows significantly low labeling efficiency relative to ABP1. For the direct comparison of the labeling efficiency between ABP N3, ABP N5, and ABP1, covalent conjugates prepared from the standard protocol were loaded onto one SDS-PAGE gel before the in-gel fluorescence scanning.

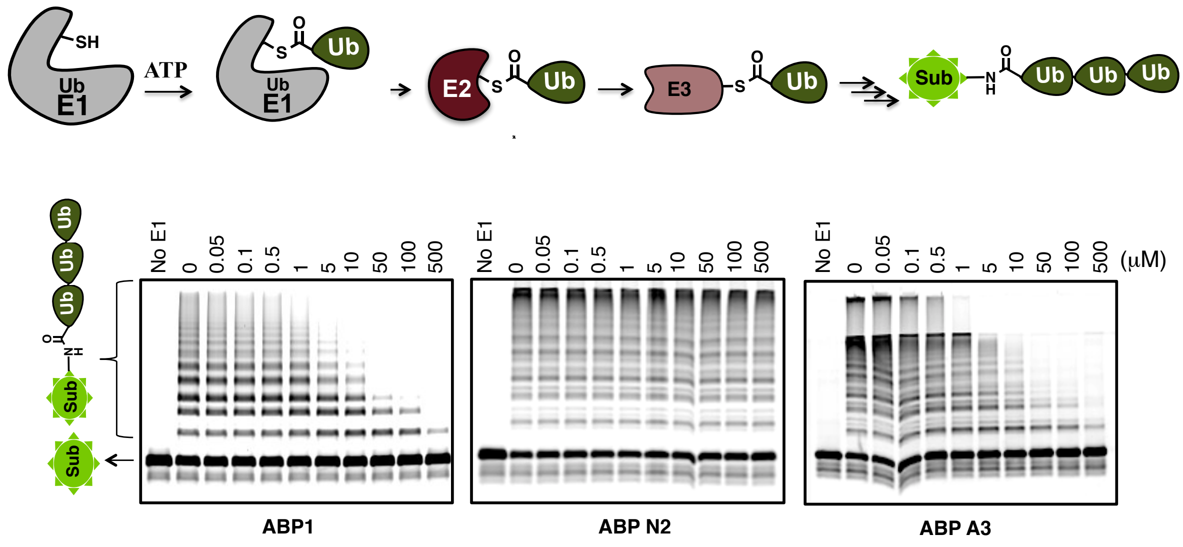

Figure S5. Assay for the inhibition of protein ubiquitination with ABP1, ABP N2, and ABP A3. (A raw data for Figure 4C)

A

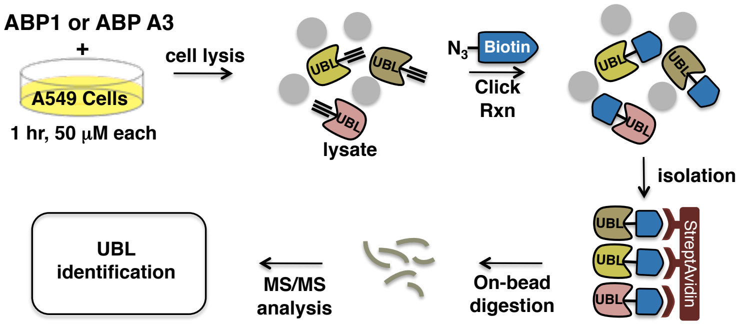

B

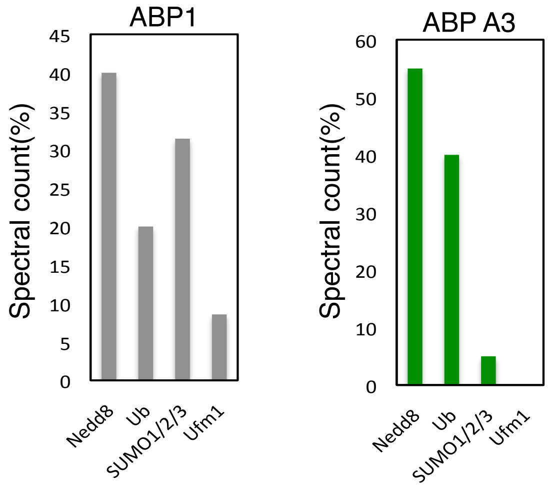

C

Probe	UBL	Sequence	# PSMs	# Proteins	# Protein Groups	q-Value	PEP	XCorr	Charge	MH+ [Da]
ABP A3	Nedd8 total SC= 11	EIEIDIEPTDKVER	2	5	1	0	0.0029	3.54	2	1685.86247
		ILGGSVLHLVLALR	4	1	1	0	8.31e-05	3.84	2	1460.93401
		ILGGSVLHLVLALRGGGGLRQ	1	1	1	0.919	1	1.06	3	2086.26743
		MLIKVKTLTGK	3	5	1	0.986	1	0.79	2	1231.77422
		TAADYKILGGSVLHLVLALR	1	1	1			0.34	2	2110.24565
	Ub total SC= 8	ESTLHLVLR	2	23	1	0	0.00987	2.88	2	1067.62358
		LRGGmQIFVKTLTGK	4	16	1	0.94	1	1.05	2	1664.96343
		TITLEVEPSDTIENVK	2	22	1	0	1.64e-05	4.17	2	1787.92461
	SUMO2/3 total SC= 1	VAGQDGSVVQFK	1	6	1	0	0.0931	2.49	2	1234.64250
ABP1	Nedd8 total SC= 14	EIEIDIEPTDKVER	6	5	1	0	0.00261	3.78	2	1685.86174
		ILGGSVLHLVLALR	4	1	1	0	0.0181	3.19	2	1460.93718
		EGIPPQQQR	1	5	1	0.688	0.95	1.25	2	1052.54607
		MLIKVKTLTGK	1	5	1	0.971	1	0.86	2	1231.77471
		ILGGSVLHLVLALRGGGGLRQ	2	1	1	0.979	1	0.76	2	2086.25957
	Ub total SC= 7	TITLEVEPSDTIENVK	2	22	1	0	0.00188	3.31	2	1787.92668
		ESTLHLVLR	2	23	1	0	0.0586	2.60	2	1067.62029
		LRGGIIEPSLRQLAQK	3	3	1	0.971	1	1.42	2	1779.07732
	SUMO2/3 total SC= 5	VAGQDGSVVQFK	1	6	1	0	0.0452	2.41	2	1234.64250
		TENNDHINLK	2	2	1	0.003	0.151	2.71	2	1197.58928
		RHTPLSK	2	5	1	0.003	0.168	2.37	2	838.49498
	SUMO1 total SC= 6	FLFEGQR	2	3	1	0	0.135	2.07	2	896.46099
		VIGQDSSEIHFK	2	4	1	0.003	0.168	2.81	2	1359.69194
		VKMTTHLK	2	4	1			1.15	2	957.54936
	Ufm1 total SC= 6	VLSVPESTPFTAVLK	3	2	1	0	0.145	2.55	2	1587.90288

Figure S6. Identification of the UBL•ABP adducts using MS analysis A549 cells were treated with **ABP1** or **ABP A3** (50 μM) for 1 hour, then lysed, conjugated to biotin-azide, and isolated by streptavidin beads. The beads were treated with trypsin, followed by the tryptic peptide analysis by LTQ Orbitrap Velos mass spectrometer (Thermo Scientific, San Jose, CA). (A) Schematic description of the MS analysis (B) The relative selectivity of UBL labeling with **ABP1** and **ABP A3** was obtained by calculating the % of each UBL spectral count. The total sum of UBL spectral counts per MS run was set as 100 %. (C) The MS data of the isolated UBLs' tryptic peptides.

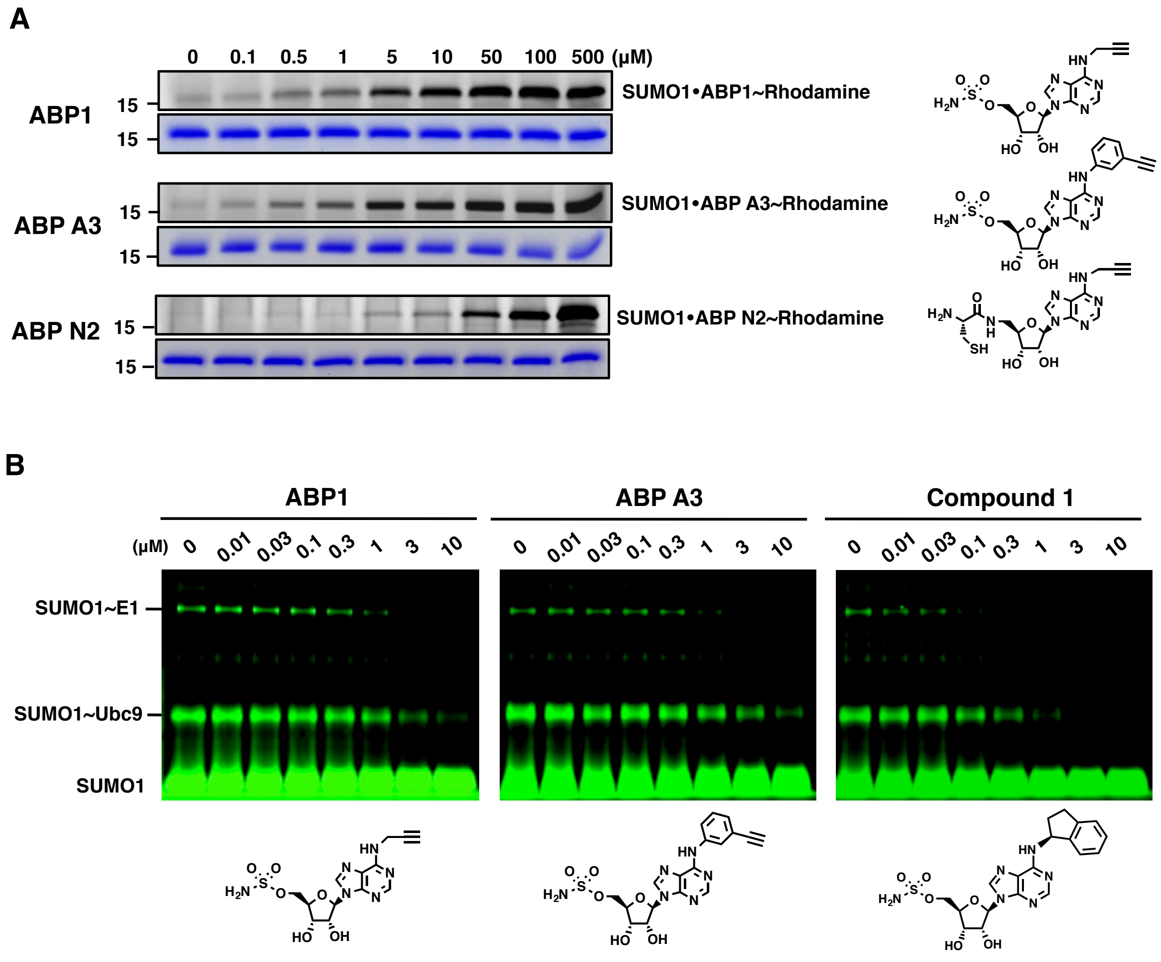

Figure S7. In-vitro comparison of the SUMO1 labeling and SUMO E1 inhibition potency of ABP1, ABP A3, ABP N2 and Compound 1. (A) The labeling of SUMO1 with **ABP1**, **ABP A3** and **ABP N2** was performed by incubating each probe with SAE1/SAE2 (0.5 μM), SUMO1 (50 μM), ATP (50 μM) and TCEP (1 mM) for 2 hours. (B) Inhibition of SAE1/SAE2 and Ubc9 trans-thiolations with **ABP1, ABP A3** and **Compound 1** was detected by incubating increasing concentration of the probes with SAE1/SAE2 (0.4 μM), Ubc9 (0.5 μM), ATP (10 μM), TCEP (0.5 mM), and FITC labeled SUMO1 (0.5 μM) for 1 hour at r.t. Proteins were separated on SDS-PAGE without DTT. FITC labeled SUMO1 was visualized by Typhoon 9600. **ABP1** and **ABP A3** showed similar inhibitory effect on SUMO E1 enzyme in these in vitro experiments.

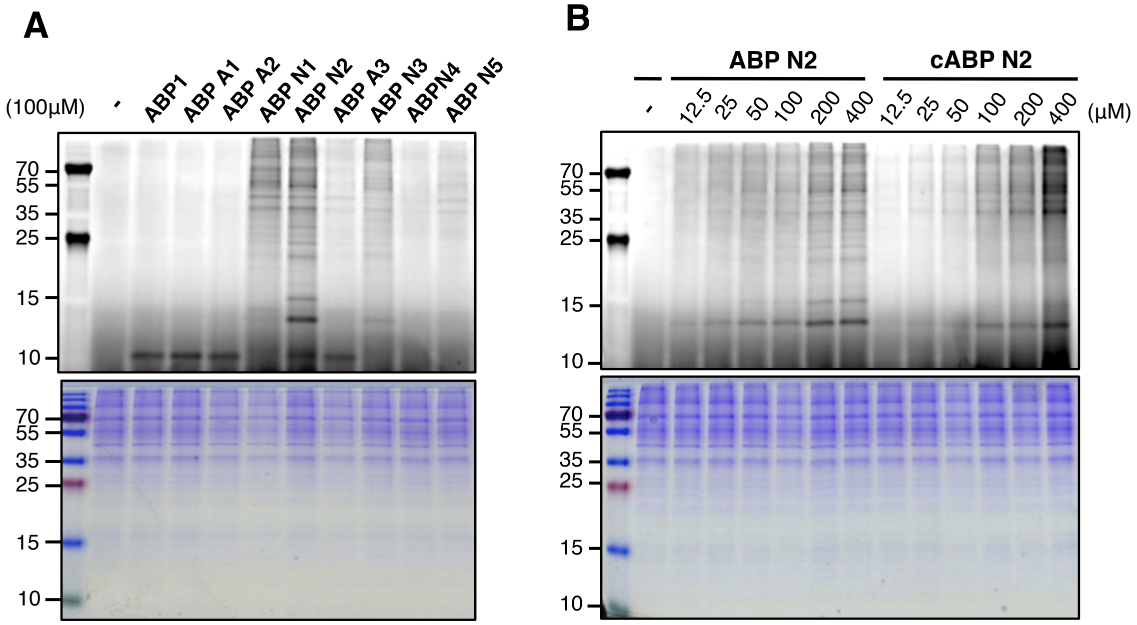

Figure S8. Labeling of proteins using ABPs in A549 cell lysate. Lysates from A549 cells were concentrated into 2 mg/ml, and treated with each **ABP** at the given concentration for 2 hours at room temperature. Conjugation to rhodamine dye and subsequent in-gel fluorescence scanning showed total proteomes labeled by each probe. The overall labeling pattern was notably different from that of live-cell experiment. Particularly, **ABP N1**, **ABP N2** and **ABP N3** labeled numerous proteins with various molecular weights in the cell extract.

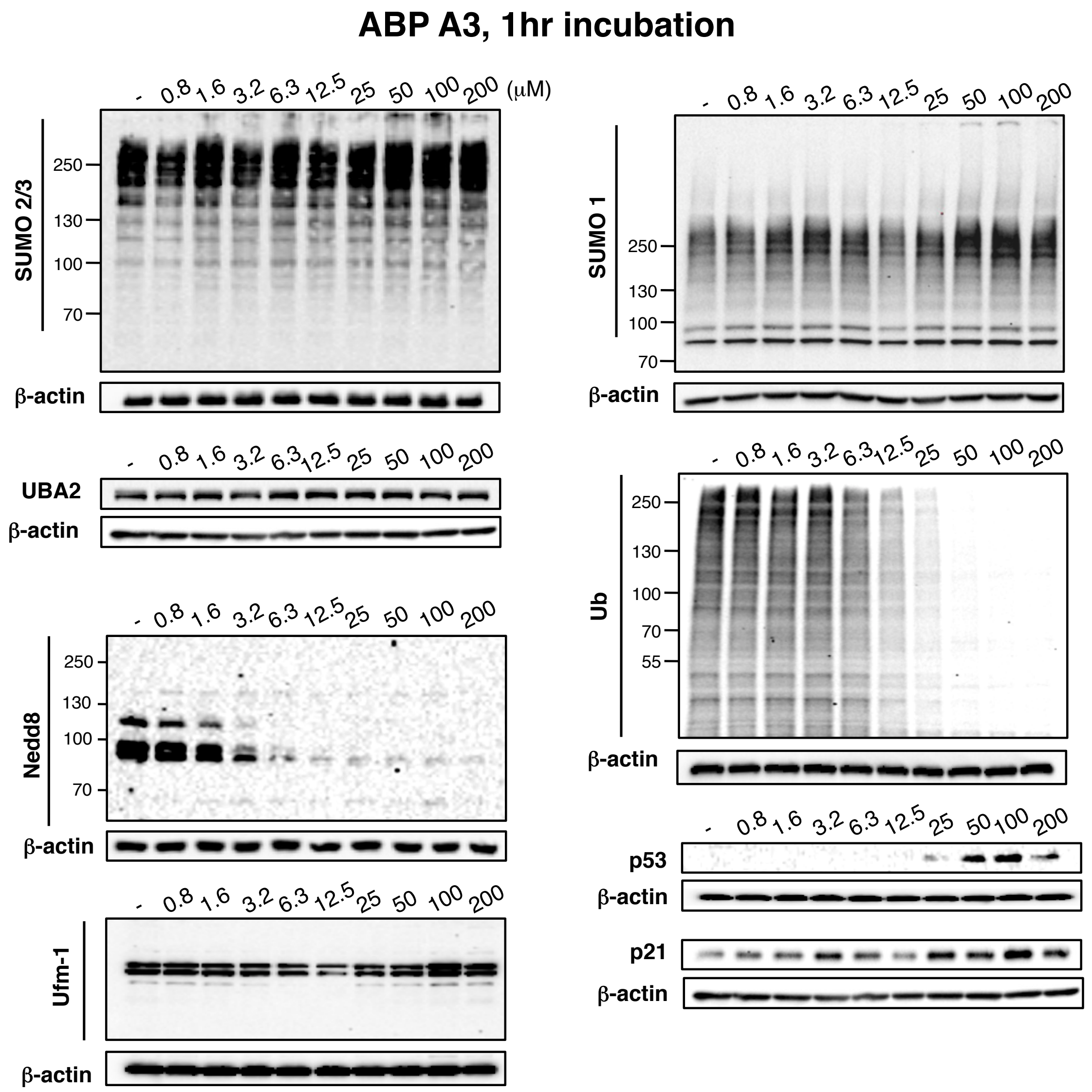

Figure S9. ABP A3 inhibits protein ubiquitination and Neddylation in 1 hour. A549 cells were incubated with different concentration of **ABP A3** for 1 hour. The cells were washed with DPBS and lysed by adding 2x SDS loading buffer directly to the cells. The lysate was boiled and subjected for immunoblotting using anti-Ub, Nedd8, SUMO1, SUMO2/3, UBA2, Ufm1, p53 and p21 antibodies. In this case, Ufm1 was resolved with 8% gel during SDS-PAGE, therefore showing the separation of the two close MW proteins. On the other hand, in the main Figure 6, Ufm1 was immunoblotted with 13% gel in order to detect free Ufm1. However, we were not able to detect free Ufm1 in this case, and the two close bands were not separated.

ABP A3, ABP 1, and Compound 1 (3hr incubation)

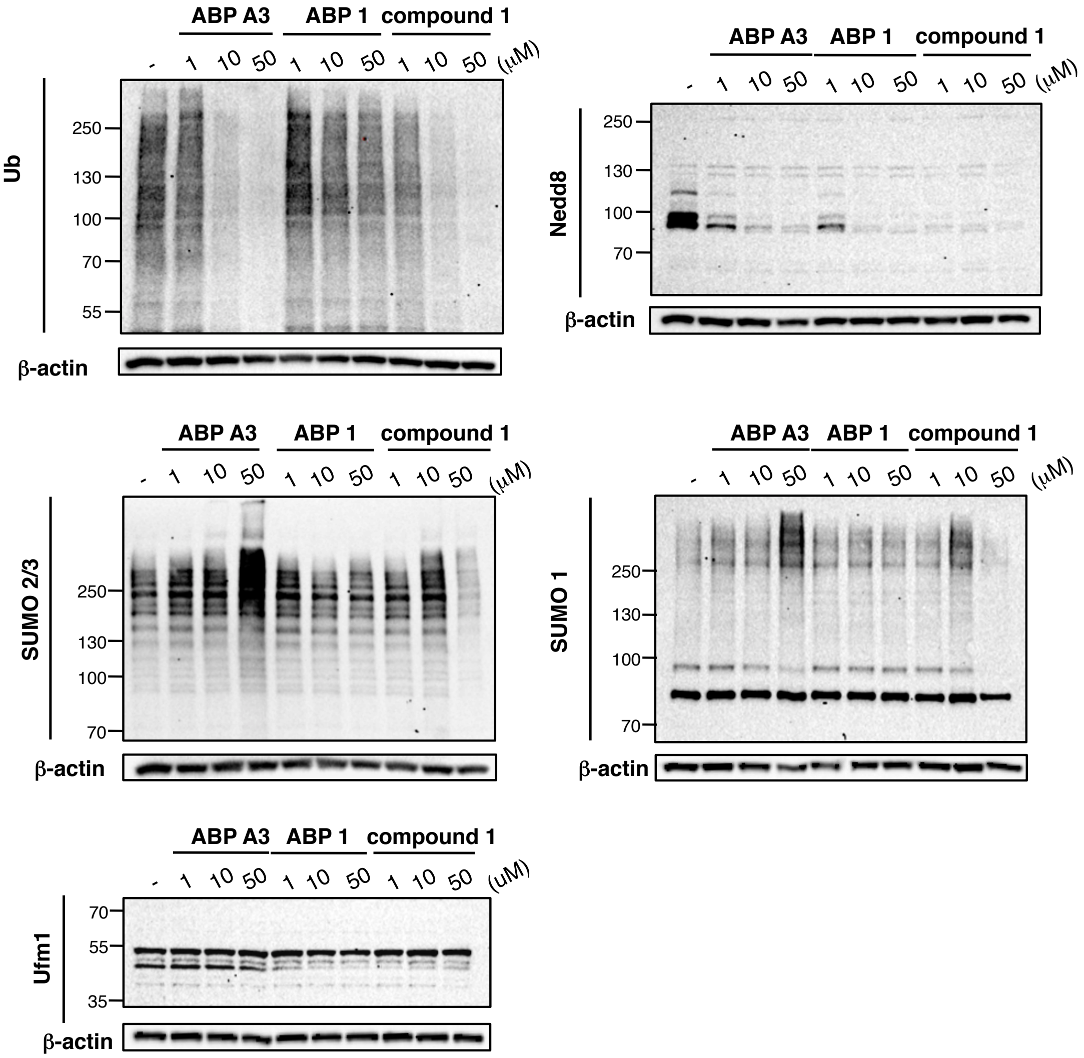

Figure S10. Comparison of ABP A3, ABP1, and Compound 1 on the inhibition of UBL conjugation systems A549 cells were incubated with each small molecule with different concentration and time. The cells were washed with DPBS and lysed by adding 2x SDS loading buffer directly to the cells. The lysate was boiled and subjected for immunoblotting using anti-Ub, Nedd8, SUMO1, SUMO2/3, and Ufm1 antibodies. **ABP1** showed low inhibitory potency to the tested UBL pathways. Compound 1 showed decrease in protein SUMOylation and Ufmylation levels at high concentration.

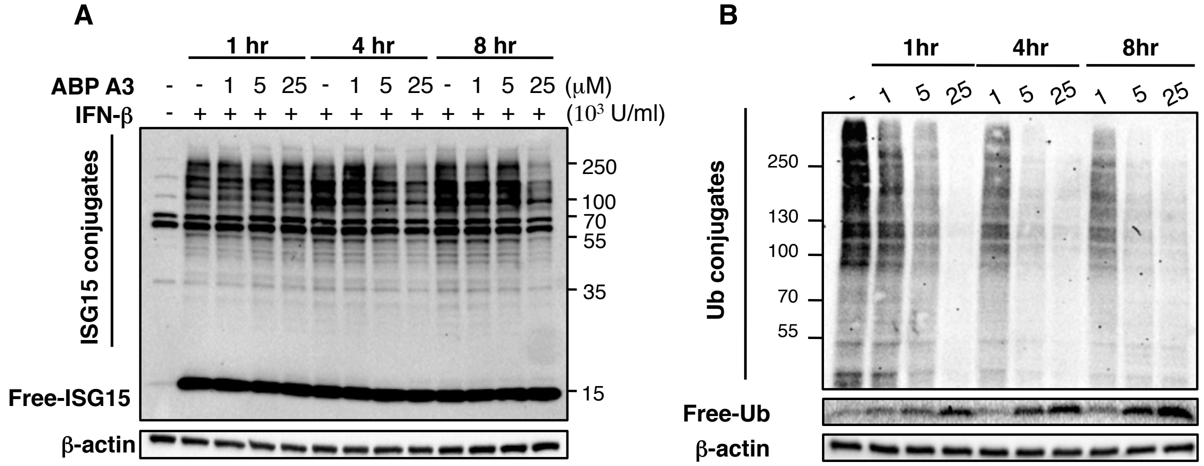

Figure S11. ISG15 conjugation system showed little sensitivity to ABP A3 treatment. (A) A549 cells were incubated with IFN-β for 12 hours to induce ISG15. Then the given concentration of **ABP A3** was added for the appeared time ranges. The resulting cell lysates were immunoblotted with anti-ISG15 antibody. The ISG15-conjugate level did not show significant decrease upon **ABP A3** treatment and free ISG15 level did not change either. (B) A549 cells were treated with different concentrations of **ABP A3** for the given time, and immunoblotted using anti-Ub antibody. In contrast to the ISGylation level in (A), **ABP A3** induced significant decrease in ubiquitin conjugates and increase in free ubiquitin level at the same condition.

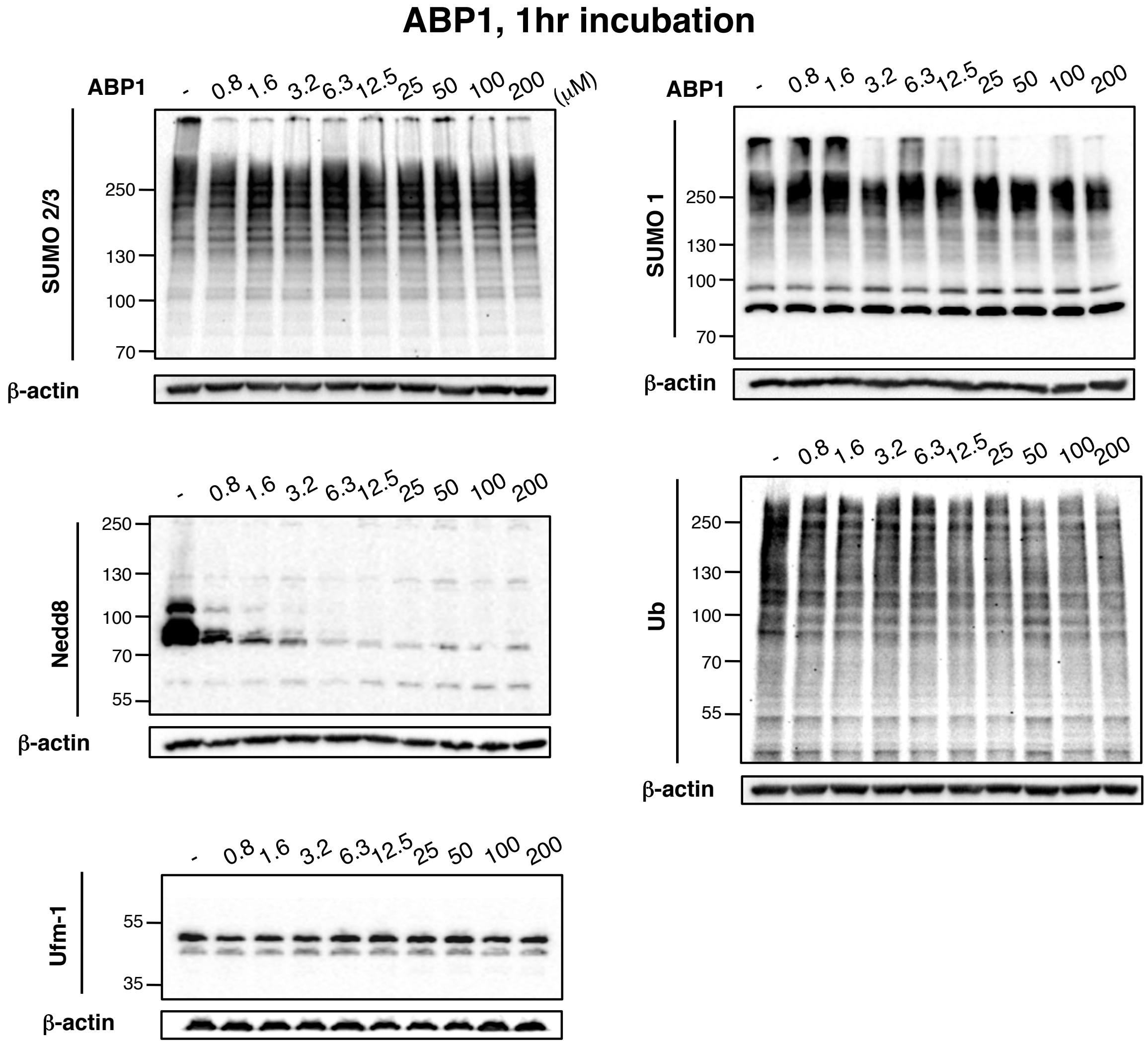

Figure S12. ABP1 has no selectivity on inhibiting protein UBL conjugation in A549 cells. A549 cells were incubated with different concentration of ABP1 for 1 hour. The lysates were immunoblotted using anti-Ub, Nedd8, SUMO1, SUMO2/3, and Ufm1 antibodies. The result well matched with in-gel fluorescent and pull-down MS analysis.

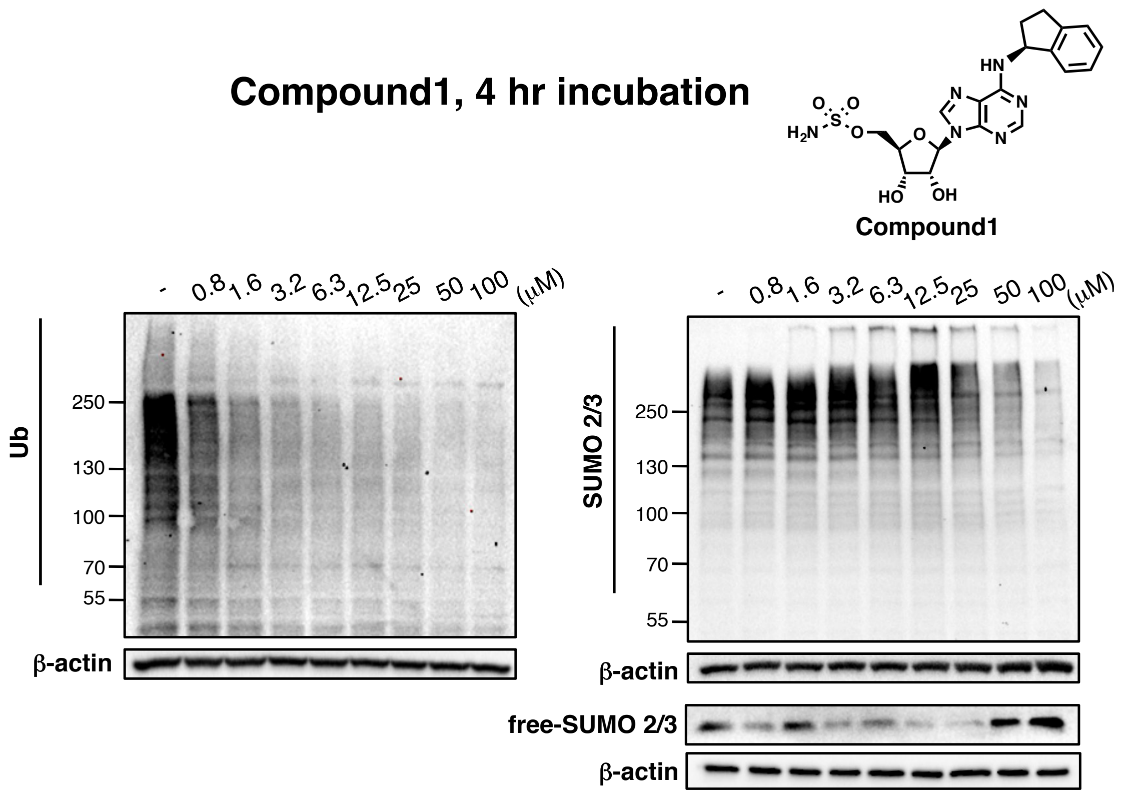

Figure S13. Compound 1 inhibits both protein ubiquitination and SUMOylation A549 cells were incubated with increasing concentration of Compound 1 for 4 hrs. The cell lysates were immunoblotted using anti-ubiquitin and anti-SUMO2/3 antibodies. SUMO2/3 Westernblot showed that Compound 1 induced the slight increase of total SUMO conjugates at the concentrations below 12.5 μM. However, the total SUMOylation level dropped when high concentration (>12.5 μM) of Compound 1 was applied.

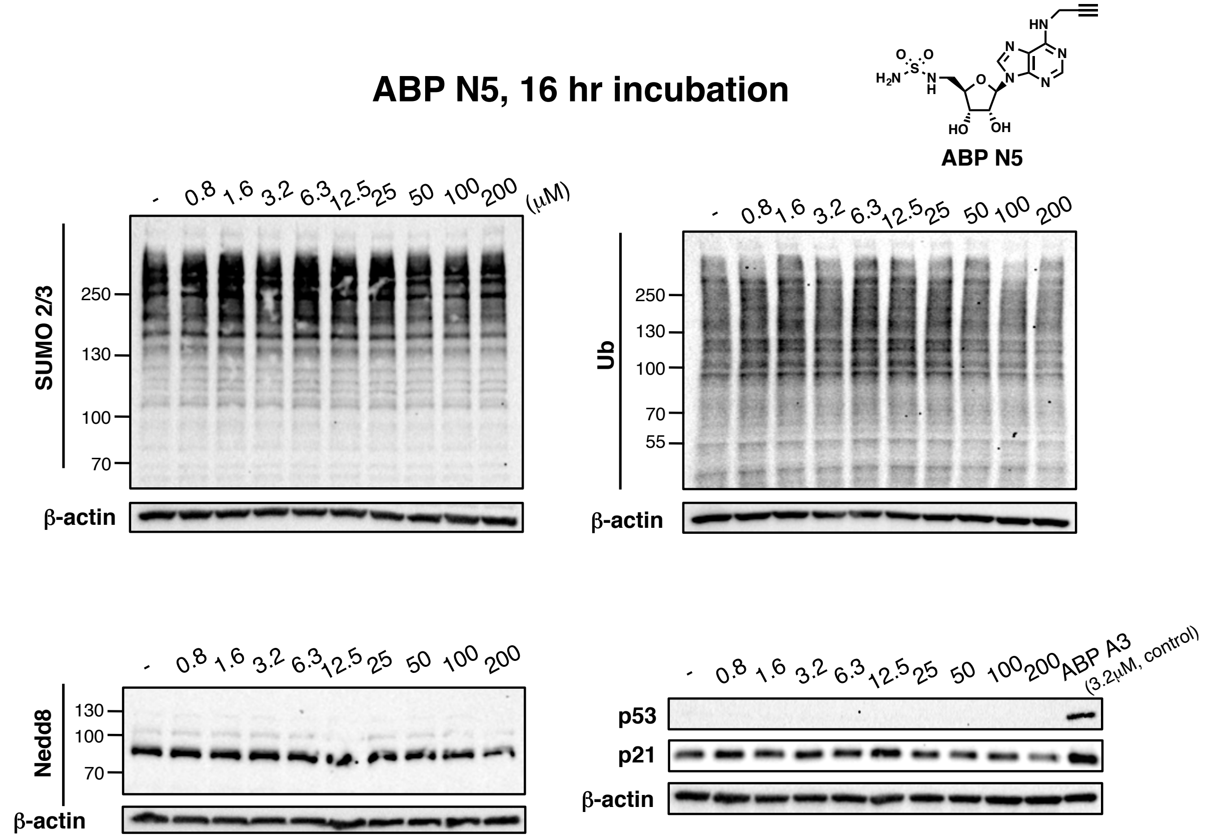

Figure S14. ABP N5 has no impact on ubiquitination, SUMOylation, Neddylation, p53 level and p21 level. A549 cells were incubated with increasing concentration of **ABP N5** for 16 hours. The lysates were immunoblotted using anti-Ub, Nedd8, SUMO2/3, p53 and p21 antibodies.

ABP A3 vs. MG132 (4 hr incubation)

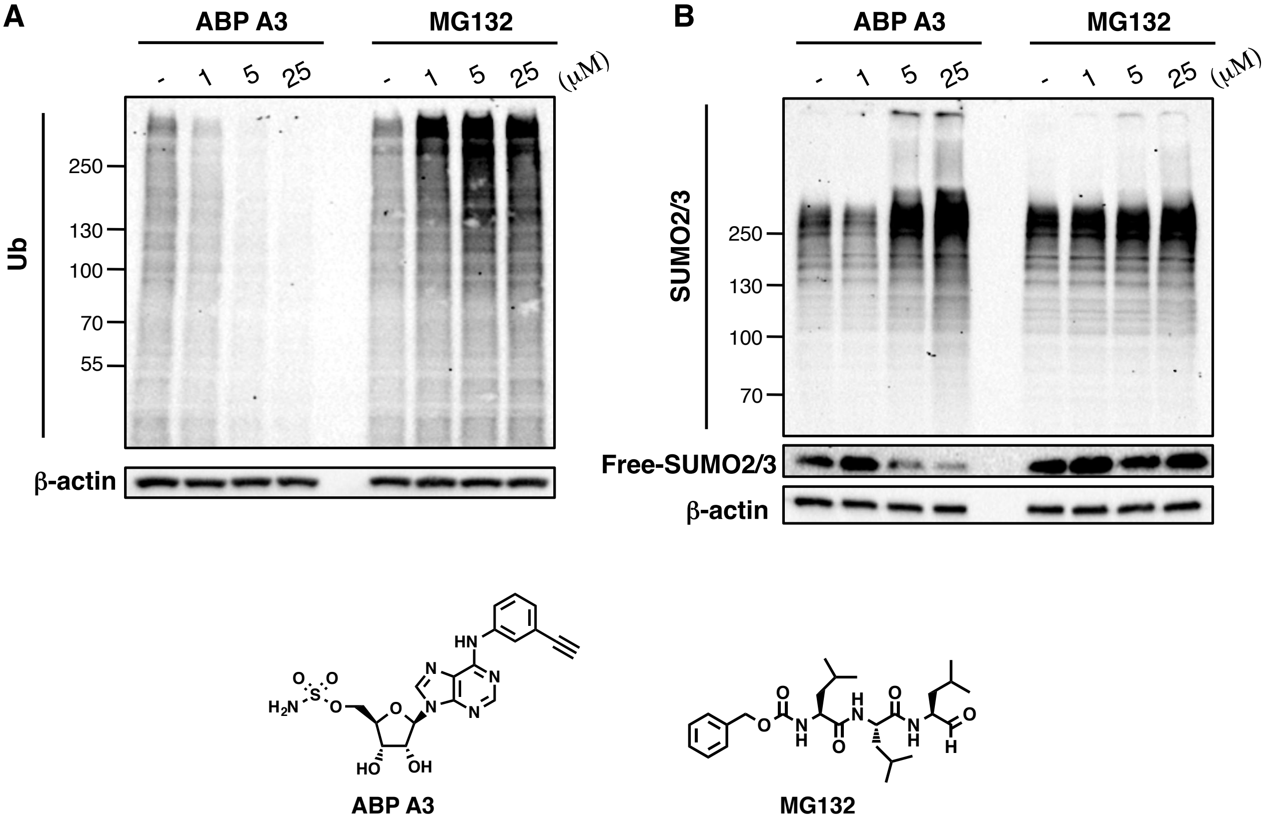

Figure S15. Comparison of ABP A3 and MG132 on ubiquitination and SUMOylation levels. A549 cells were treated with ABP A3 or MG132 (proteasome inhibitor) for 4 hours at different concentration. Immunoblotting with anti-Ub and anti-SUMO2/3 antibody showed the expected decrease of ubiquitin conjugates in ABP A3 treated cells and increase of ubiquitin conjugates in MG132 treated cells. However, both compounds showed increase of SUMO2/3 conjugate level, with **ABP A3** exhibiting more distinctive increase. The results suggest the induction of cellular stress response in both ubiquitin-proteasome inhibitors, albeit the different cellular targets.

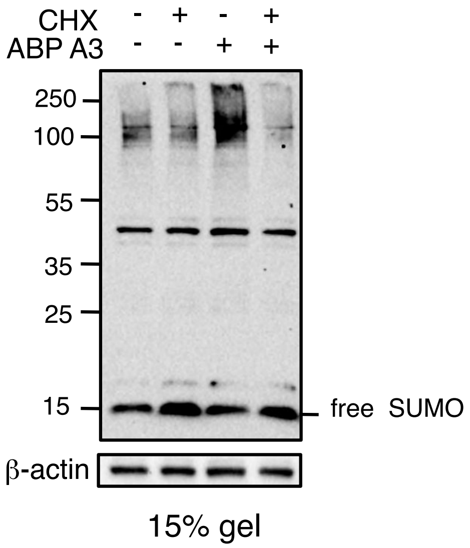

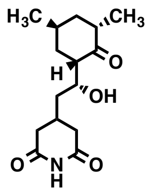

Cycloheximide

Figure S16. Immunoblotting of the same samples prepared for figure 9C using 15% gel revealed the concomitant accumulation of free-SUMO2/3 upon the treatment of cycloheximide (protein translation inhibitor).

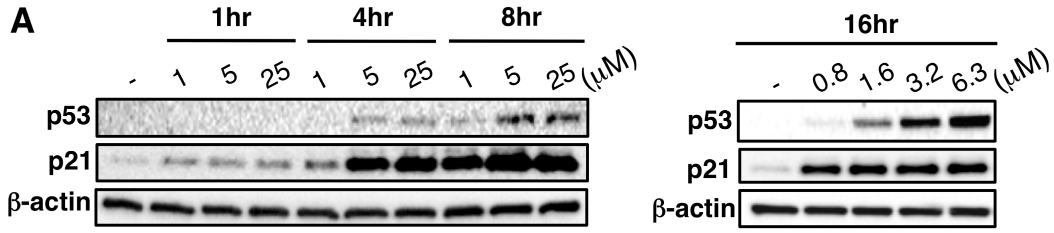

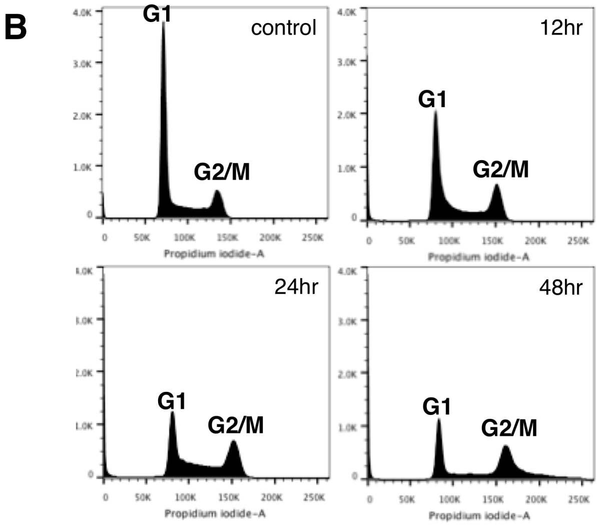

C

	G0/G1	S	G2/M
DMSO	56 %	32 %	12 %
12 hr	43 %	38 %	19 %
24 hr	27 %	48 %	24 %
48 hr	30 %	27 %	43 %

Figure 17. Effect of ABP A3 on p53 and p21 protein levels, and the cell cycle in A549 cells (A) A549 cells were treated with ABP A3, followed by immunoblotting with anti-p53 and p21 antibodies. (B) A549 cells were harvested after 12, 24 and 48 hours of treatment with ABP A3 (2.5 μM), and stained with propidium iodide. DNA content was analyzed by flow cytometry. (C) Population of cells at each cell cycle phase was quantified based on B.

II. Synthesis

General information.

Methanol (ACS grade), ethyl acetate (ACS grade, and HPLC grade), hexane (ACS grade), acetonitrile (ACS grade), chloroform (ACS grade) and acetone (ACS grade) were purchased from Fisher Scientific and used without further purification. Dichloromethane and toluene were purified by passing over activated alumina. Commercially available reagents were used without further purification. Reactions were monitored by thin-layer chromatography (TLC) on pre-coated glass-backed plates (60 Å silica gel, 0.25mm, Whatman), and components were visualized by UV light (254 and 365 nm) or by treating the plates with anisaldehyde, $KMnO_4$, and ninhydrin stains followed by heating. Flash column chromatography was performed over ultra pure silica gel (230-400 mesh) from Silicycle. ^{1}H and ^{13}C NMR spectra were obtained on Bruker AVANCE III 500 MHz spectrometers. Chemical shifts were reported in ppm relative to the residual solvent peak ($CDCl_3$, ^{13}C 77.00; TMS: 0.00). Multiplicity was indicated as follows: s (singlet); d (doublet); t (triplet); q (quartet); m (multiplet); dd (doublet of doublets); ddd (doublet of doublet of doublets); dt (doublets of triplets); td (triplet of doublets); brs (broad singlet). Coupling constants were reported in Hz. High resolution mass analysis of the final ABP analogs were conducted using LC-TOF: Agilent 6210A. Mass analysis of the other intermediates were conducted using LC-MS: Bruker AmaZon SL Ion Trap and LC-MS: Bruker AmaZon X Ion Trap.

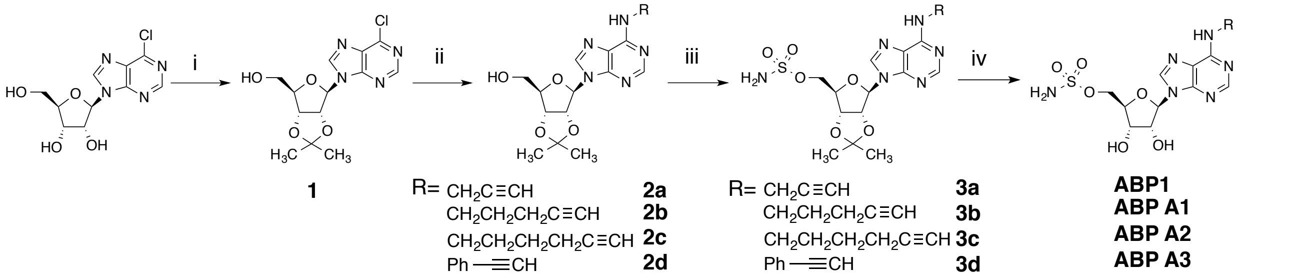

Scheme S1. Synthesis of affinity analogues (i) 2, 2-dimethoxypropane (5eq), p-toluenesulfonic acid monohydrate (1eq), acetone, r.t, 2 hrs (ii) acetylene reagent (2 eq), neat, 110 °C, 1 hr (iii) sulfamoyl chloride (3eq), triethyl amine (1eq), DMF, 0°C, 1 hr (iv) CH_2Cl_2, TFA/ddH_2O=4:1, 0°C, 1 hr.

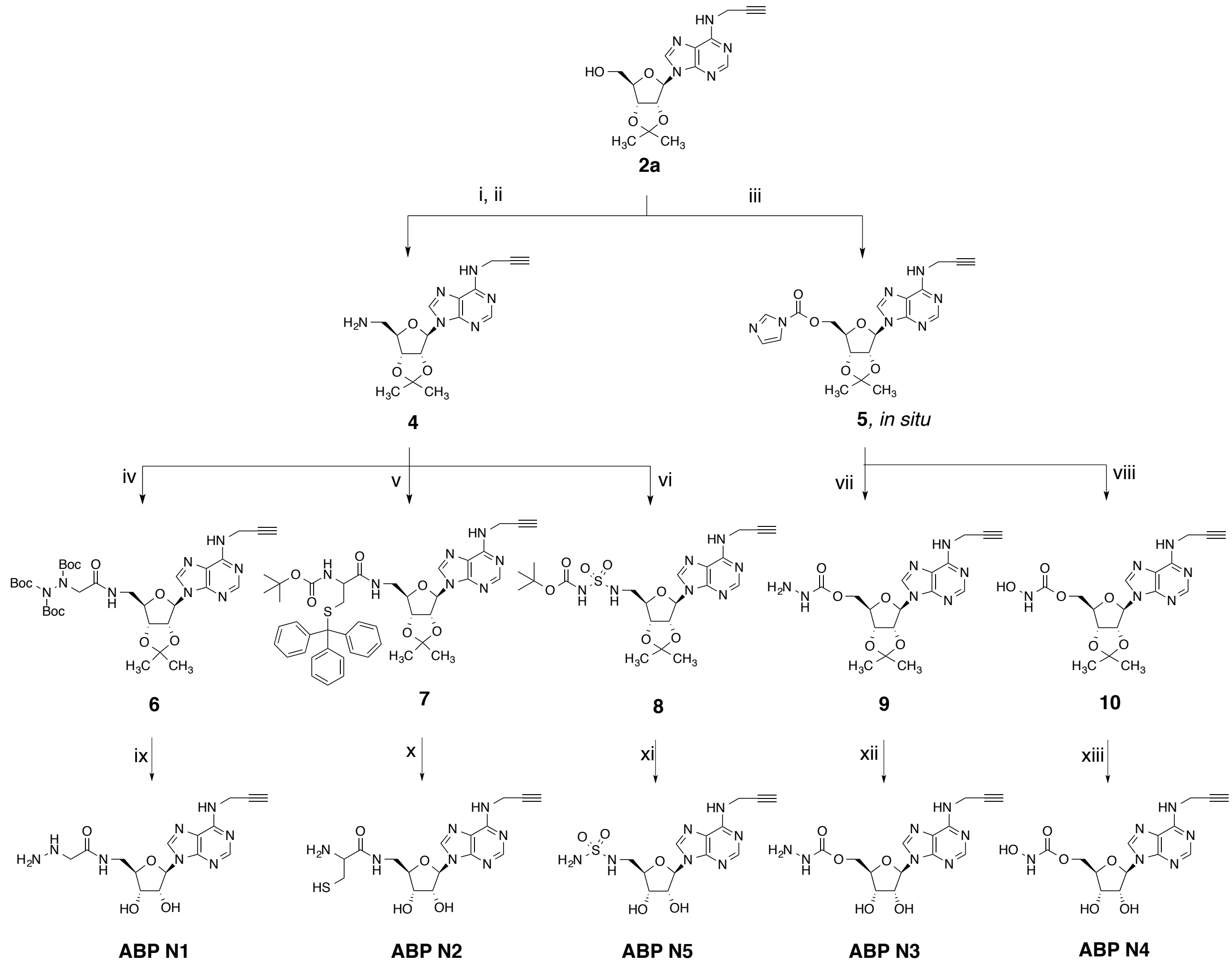

Scheme S2. (i) phthalimide (1.2eq), triphenylphosphine (1.3eq), diisopropyl azodicarboxylate (1.5eq), THF, 0°C~r.t, 4 hrs (ii) hydrazine (10eq), EtOH, reflux, 30min (iii) 1,1'-carbonyldiimidazole (1.2eq), ACN, 0°C, 1hr (iv) Tri-Boc-hydrazinoacetic acid (1.1eq), HATU (1.2eq), diisopropylethylamine, ACN, 0°C. (v) (Boc)-Cys(Trt)-OH (1.1eq), HATU (1.2eq), diisopropylethylamine, ACN, 0°C. (vi) Boc-sulfamyl chloride (1.5eq), TEA (3eq), CH_2Cl_2, 0°C, 30min (vii) hydrazine solution (80%, 2eq), ACN, 0°C, 1hr (viii) hydroxylamine hydrochloride salt

(excess), sodium carbonate (2eq), THF/H_2O, 0 °C~r.t, 3hr (ix) hydrazine (2eq), CH_2Cl_2, TFA/ddH_2O=4:1, 0°C, 1 hr (x) Et_3SiH (4eq), CH_2Cl_2, TFA/ddH_2O=4:1, 0°C, 1 hr (xi) CH_2Cl_2, TFA/ddH_2O=4:1, 0°C, 1 hr (xii) hydrazine (2eq), CH_2Cl_2, TFA/ddH_2O=4:1, 0°C, 1 hr (xiii) hydroxyl amine (2eq), CH_2Cl_2, TFA/ddH_2O=4:1, 0°C, 1 hr

Synthesis of compound 1

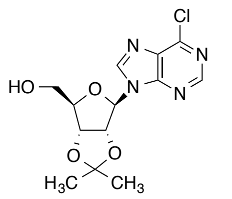

To a flask containing 6-chloropurine riboside (1.50 g, 5.23 mmol, 1 eq.) in anhydrous acetone (50 mL), was added 2, 2-dimethoxypropane (3.21 mL, 26.15 mmol, 5 eq.) and p-toluenesulfonic acid monohydrate (995 mg, 5.23 mmol, 1 eq.). The reaction mixture was stirred at room temperature for 2 hrs. After two hours of the reaction time saturated sodium bicarbonate solution was added slowly to the reaction mixture until the pH reached 8.0. We noticed that when we added sodium bicarbonate to the reaction mixture, white precipitate started to form immediately. This white solid precipitate was collected by vacuum filtration, washed with cold acetone and dried thoroughly under high vacuum to provide Compound S1 (1.56 g, 97 %) as a white solid; TLC R_f = 0.53 (MeOH:CH_2Cl_2 = 1:10); ^{1}H NMR (500 MHz, $CDCl_3$) δ 1.40 (s, 3H), 1.67 (s, 3H), 3.85 (ddd, J = 12.5, 10.3, 2.1 Hz, 1H), 4.00 (dt, J = 12.5, 2.1 Hz, 1H), 4.58 (q, J = 1.8 Hz 1H), 5.08 (dd, J = 10.4, 2.4 Hz, 1H), 5.13 (dd, J = 6.0, 1.5 Hz, 1H), 5.22 (dd, J = 5.9, 4.6 Hz, 1H), 6.01 (d, J = 4.6 Hz, 1H), 8.29 (s, 1H), 8.78 (s, 1H); ^{13}C NMR (125 MHz, $CDCl_3$): δ 25.2, 27.5, 63.2, 81.5, 83.3, 86.3, 94.1, 114.5, 133.2, 144.8, 150.4, 151.7, 152.3. HRMS calcd for $C_{13}H_{15}ClN_4O_4$ $[M + H]^+$: 327.08; Found: *m/z* 327.0857

Synthesis of compound 2a

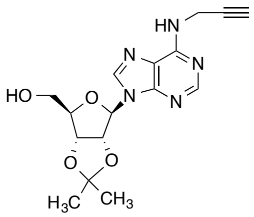

To a 10 mL round-bottom flask charged with compound 1 (1.0 g, 3.06 mmol, 1 eq.) and EtOH (2 mL), was added propargylamine (392 μL, 6.12 mmol, 2 eq.). The reaction mixture was stirred at 110 °C for one hour without a cap in order to let the ethanol evaporate slowly. The reaction progress was monitored by TLC, which showed that the reaction was completed after 1 hour of the reaction time. The reaction mixture was cooled to 0 ºC, followed by the addition of diethyl ether to yield brown precipitate, which was collected by vacuum filtration. Brown precipitate was washed with cold EtOH (20 mL) followed by cold diethyl ether (20 mL). We found that running the reaction in neat condition rather than adding solvent such as n-BuOH led to 100% conversion of the starting material and higher yield in shorter reaction time (1 hr vs. 5 hrs). Collected crystals were dried under high vacuum to yield compound 2a (943.8 mg, 94.6 %) as a white solid; TLC R_f 0.62 (MeOH:CH_2Cl_2 = 1:10), 0.23 (EtOAc:n-hexane = 2:1); ^{1}H NMR (500 MHz, $CDCl_3$) δ 1.39 (s, 3H), 1.66 (s, 3H), 2.30 (q, J = 2.1 Hz, 1H), 3.50 (qd, J = 7.0, 1.5 Hz, 1H), 3.76-3.86 (m, 1H), 3.99 (dt, J = 12.7, 1.6 Hz, 1H), 4.45-4.50 (m, 2H), 4.56 (t, J = 1.6 Hz, 1H), 5.13 (dd, J = 5.8, 1.1 Hz, 1H), 5.22 (t, J = 5.4 Hz, 1H), 5.87 (d, J = 4.9 Hz, 1H), 6.43 (s, 1H), 6.68 (d, J = 11.6 Hz, 1H), 7.85 (s, 1H), 8.41 (s, 1H); ^{13}C NMR (125 MHz, $CDCl_3$): δ 25.3, 27.7, 63.5, 65.9, 71.8, 79.7, 81.7, 82.9, 86.0, 94.4, 114.0, 121.5, 140.1, 152.6, 154.3. HRMS calcd for $C_{16}H_{19}N_5O_4$ $[M + H]^+$: 346.14; Found: *m/z* 346.1510

Synthesis of compound 2b

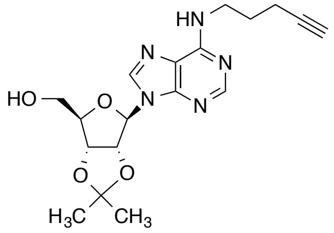

White solid; Yield = quantitative; TLC R_f 0.67 (MeOH:CH2Cl2 = 1:10) ^{1}H NMR (500 MHz, Chloroform-*d*) δ 8.35 (s, 1H), 7.80 (s, 1H), 6.84 (d, *J* = 11.8 Hz, 1H), 6.16 (s, 1H), 5.86 (d, *J* = 5.0 Hz, 1H), 5.22 (t, *J* = 5.4 Hz, 1H), 5.13 (dd, *J* = 5.7, 1.1 Hz, 1H), 4.56 (s, 1H), 4.00 (dt, *J* = 13.0, 1.6 Hz, 1H), 3.85 – 3.75 (m, 3H), 2.35 (td, *J* = 6.9, 2.7 Hz, 2H), 2.04 (t, J = 2.7 Hz, 1H), 1.94 (q, J = 6.7 Hz, 2H), 1.66 (s, 3H), 1.39 (s, 3H). ^{13}C NMR (126 MHz, CDCl3) δ 155.30, 152.73, 139.58, 113.93, 94.46, 86.00, 83.27, 82.93, 81.76, 69.39, 63.48, 39.59, 28.06, 27.71, 25.25, 16.00. MS calcd for $C_{18}H_{23}N_5O_4$ $[M + H]^+$: 374.18; Found: *m/z* 373.94

Synthesis of compound 2c

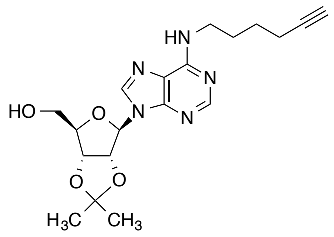

White solid; Yield = quantitative; TLC R_f 0.68 (MeOH:CH2Cl2 = 1:10), ^{1}H NMR (500 MHz, Chloroform-*d*) δ 8.34 (s, 1H), 7.79 (s, 1H), 6.85 (d, *J* = 11.8 Hz, 1H), 6.02 (s, 1H), 5.85 (d, *J* = 5.0 Hz, 1H), 5.22 (t, *J* = 5.4 Hz, 1H), 5.13 (dd, *J* = 5.8, 1.1 Hz, 1H), 4.56 (d, *J* = 1.8 Hz, 1H), 3.99 (dt, *J* = 12.9, 1.7 Hz, 1H), 3.80 (td, *J* = 12.9, 12.4, 1.9 Hz, 1H), 3.68 (d, *J* = 8.2 Hz, 2H), 2.27 (td, *J* = 7.0, 2.7 Hz, 2H), 1.99 (t, *J* = 2.6 Hz, 1H), 1.87 – 1.78 (m, 2H), 1.70 – 1.63 (m, 5H), 1.39 (s, 3H). ^{13}C NMR (126 MHz, CDCl3) δ

155.29, 152.76, 139.48, 113.92, 94.43, 86.00, 83.92, 82.94, 81.76, 68.83, 63.47, 40.04, 28.62, 27.70, 25.60, 25.25, 18.13. MS calcd for $C_{19}H_{25}N_5O_4$ $[M + H]^+$: 388.19; Found: *m/z* 387.95

Synthesis of compound 2d

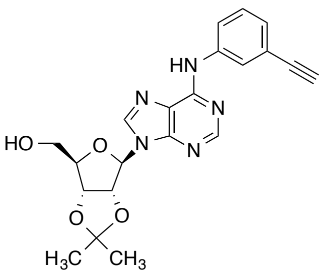

White solid; Yield = 57%; ^1H NMR (500 MHz, Chloroform-*d*) δ 8.47 (s, 1H), 7.96 (t, *J* = 1.8 Hz, 1H), 7.87 (s, 1H), 7.86 (s, 1H), 7.76 (ddd, *J* = 8.5, 2.3, 1.1 Hz, 1H), 7.31 (t, *J* = 7.9 Hz, 1H), 7.24 (dd, 7.7, 1.3 Hz, 1H), J = 6.40 (dd, *J* = 11.7, 2.0 Hz, 1H), 5.86 (d, *J* = 4.9 Hz, 1H), 5.21 (t, *J* = 5.4 Hz, 1H), 5.10 (dd, *J* = 5.8, 1.4 Hz, 1H), 4.53 (d, *J* = 2.0 Hz, 1H), 3.96 (tt, *J* = 12.0, 1.7 Hz, 1H), 3.79 (ddd, *J* = 13.0, 11.6, 2.0 Hz, 1H), 3.08 (s, 1H), 1.63 (s, 3H), 1.36 (s, 3H). ^{13}C NMR (126 MHz, CDCl3) δ 152.52, 148.05, 140.51, 138.31, 129.08, 127.64, 123.76, 122.91, 121.94, 120.93, 114.10, 94.44, 86.14, 83.35, 83.05, 81.73, 63.42, 27.68, 25.26. LRMS calcd for $C_{21}H_{21}N_5O_4$ $[M + H]^+$: 408.16; Found: *m/z* 407.94

Synthesis of Sufamoyl Chloride

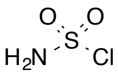

To a 10 mL round-bottom flask charged with chlorosulfonyl isocyanate (600 μL, 6.85 mmol) under N_2 in ice bath, was added formic acid (285.5 μL, 6.85 mmol) dropwise over 5 min with vigorous stirring. 10 min later, the reaction temperature was raised to room temperature. Within 5 min, generation of white fog was detected in the flask. After another

10 min, 2 mL of acetonitrile was added to the flask, which generated gas with foaming of the mixture. One hour later, the reaction mixture was concentrated under the reduced pressure (oil pump, overnight) to provide sulfamoyl chloride as a white solid, which was directly used without purification for the synthesis of **compound 3a – 3d**.

Synthesis of compound 3a

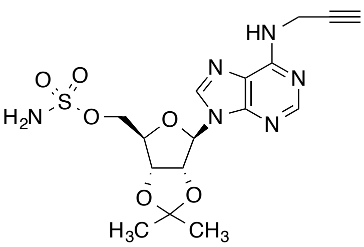

To a 10 ml round-bottom flask containing Compound S2 (100 mg, 0.29 mmol, 1 eq.) in DMF (1 mL), sulfamoyl chloride (100.4 mg, 0.87 mmol, 3 eq.) was added, followed by the slow addition of triethyl amine (40.4 µL, 0.29 mmol, 1.0 eq.) at 0 ºC. The reaction solution was stirred at r.t. for 1 hr. DMF was evaporated under high vacuum (oil pump, overnight). The resulting residue was dissolved in ddH_2O and extracted with EtOAc (3×20 mL). Combined organic layers were dried with sodium sulfate and filtered. The filtrate was concentrated under the reduced pressure and the residue was purified with silica gel flash column chromatography (MeOH:CH_2Cl_2 = 1:20) to provide compound S3 (120.2 mg, 98%) as a white solid. TLC R_f = 0.43 (MeOH:CH_2Cl_2 = 1:9); ^{1}H NMR (500 MHz, $CDCl_3$) δ 1.39 (s, 3H), 1.63 (s, 3H), 2.29 (t, *J* = 2.5 Hz, 1H), 4.25-4.51 (m, 3H), 4.57 (dt, *J* = 6.2, 3.4 Hz, 1H), 5.12 (dd, *J* = 6.3, 3.0 Hz, 1H), 5.39 (dd, *J* = 6.3, 2.5 Hz, 1H), 5.97 (s, 2H), 6.14 (d, *J* = 2.5 Hz, 1H), 6.45 (s, 1H), 7.96 (s, 1H), 8.41 (s, 1H); ^{13}C NMR (125 MHz, $CDCl_3$): δ 25.3, 27.1, 53.5, 69.5, 71.6, 80.1, 81.0, 83.9, 84.3, 90.9, 114.9, 120.1,

139.5, 153.3, 153.9. HRMS calcd for $C_{16}H_{20}N_6O_6S$ $[M + H]^+$: 425.12; Found: *m/z* 425.1250

Synthesis of compound 3b

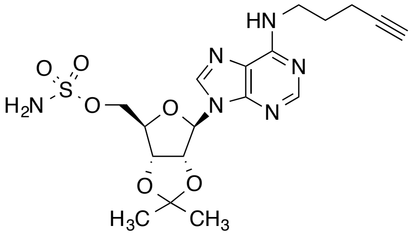

White solid; Yield = 89.2%. TLC R_f = 0.47 ($MeOH:CH_2Cl_2$ = 1:10); ^1H NMR (500 MHz, Chloroform-*d*) δ 8.33 (s, 1H), 8.08 (s, 1H), 6.58 (s, 1H), 6.16 (d, *J* = 2.6 Hz, 1H), 5.29 (dd, *J* = 6.3, 2.7 Hz, 1H), 5.06 (dd, *J* = 6.4, 2.9 Hz, 1H), 4.54 (q, *J* = 4.0 Hz, 1H), 4.40 (ddd, *J* = 13.7, 8.0, 4.1 Hz, 2H), 3.80 – 3.62 (m, 2H), 2.30 (td, J = 7.1, 2.7 Hz, 2H), 2.03 (t, J = 2.7 Hz, 1H), 1.91 – 1.87 (m, 2H), 1.59 (s, 3H), 1.35 (s, 3H). ^{13}C NMR (126 MHz, CDCl3) δ 154.71, 153.42, 138.86, 119.51, 114.67, 90.40, 84.27, 83.78, 81.07, 69.25, 60.45, 36.57, 27.40, 25.26, 21.10, 16.02. MS calcd for $C_{18}H_{24}N_6O_6S$ $[M + H]^+$: 453.15; Found: *m/z* 452.93

Synthesis of compound 3c

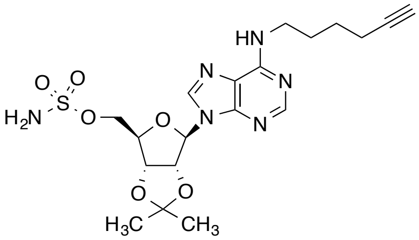

White solid; Yield = quantitative TLC R_f = 0.51 ($MeOH:CH_2Cl_2$ = 1:10); ^1H NMR (500 MHz, Chloroform-*d*) δ 8.33 (s, 1H), 8.07 (s, 1H), 6.42 (s, 1H), 6.16 (d, *J* = 2.7 Hz, 1H), 5.30 (dd, *J* = 6.4, 2.7 Hz, 1H), 5.09 – 5.04 (m, 1H), 4.56 – 4.52 (m, 1H), 4.41 (t, *J* = 5.2 Hz, 1H), 3.70 – 3.55 (m, 3H), 2.24 (td, *J* = 7.0, 2.6 Hz, 2H), 1.99 (t, *J* = 2.7 Hz, 1H), 1.81 – 1.75 (m, 2H), 1.67 – 1.61 (m, 2H), 1.60 (s, 3H), 1.35 (s, 3H). ^{13}C NMR (126

MHz, CDCl3) δ 154.72, 153.41, 138.76, 119.46, 114.69, 90.42, 84.25, 84.08, 81.07, 69.25, 60.45, 27.09, 25.68, 25.26, 21.09, 18.12, 14.21. MS calcd for $C_{19}H_{26}N_6O_6S$ $[M + H]^+$: 467.16; Found: *m/z* 466.96

Synthesis of compound 3d

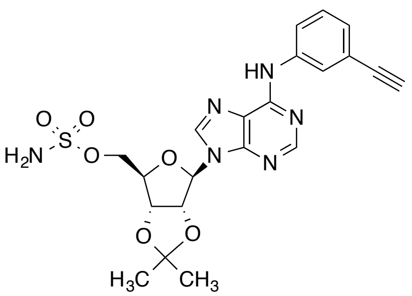

White solid; Yield = quantitative. TLC R_f = 0.45 (MeOH:CH_2Cl_2 = 1:10); ^1H NMR (500 MHz, Chloroform-d) δ 8.41 (s, 1H), 8.00 (d, *J* = 7.9 Hz, 2H), 7.94 (d, *J* = 1.9 Hz, 2H), 7.71 – 7.66 (m, 1H), 7.24 (t, *J* = 7.9 Hz, 1H), 7.16 (dt, *J* = 7.6, 1.3 Hz, 1H), 6.55 (brs, 1H), 6.12 (d, *J* = 2.3 Hz, 1H), 5.32 – 5.24 (m, 1H), 5.01 (dd, *J* = 6.3, 2.8 Hz, 1H), 4.51 (dt, *J* = 6.0, 3.3 Hz, 1H), 4.35 (qd, *J* = 11.0, 4.2 Hz, 2H), 3.08 (s, 1H), 1.55 (s, 3H), 1.31 (s, 3H). LRMS calcd for $C_{13}H_{16}N_6O_6S$ $[M + H]^+$: 487.13; Found: *m/z* 486.98

Synthesis of ABP1

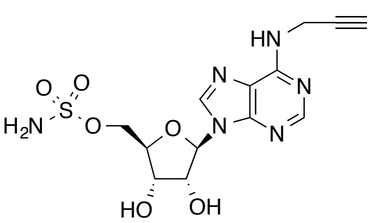

A flask containing compound S3 (10 mg, 0.024 mmol, 1 eq.) in CH_2Cl_2 (150 μL), was treated with a solution of TFA in ddH_2O (4:1 v/v, total volume 200 μL) at 0 ºC. The reaction mixture was stirred at r.t. for 1hr. The reaction progress was monitored by thin

layer chromatography. CH_2Cl_2, TFA and ddH_2O were removed under high vacuum (oil pump, overnight). The remaining residue was purified by reverse-phase high performance liquid chromatography (*rp*-HPLC, solvent A: 95% ddH_2O, 5% acetonitrile, 0.1% TFA, solvent B: 95% acetonitrile, 5% ddH_2O, 0.1% TFA. Gradient method: a) 0-4 min: solvent A(100%), solvent B(0%); b) 4-19 min: solvent A (100→0%), solvent B (0→100%), gradient; c) 19-27 min: solvent A (0%), solvent B (100%), the flow rate of the mobile phases was kept at 20mL min^{-1}). Collected fractions were lyophilized to yield **ABP1** (7.17 mg, 79%) as a white solid; TLC R_f = 0.53 (MeOH:CH_2Cl_2 = 5:1, 1% TEA)); ^1H NMR (500 MHz, MeOD) δ 2.78 (t, *J* = 2.4 Hz, 1H), 3.31-3.35 (m, 2H), 4.33-4.48 (m, 4H), 4.68 (t, *J* = 5.0 Hz, 1H), 6.13 (d, *J* = 4.9 Hz, 1H), 8.40 (s, 1H), 8.42 (s, 1H); ^1H NMR (500 MHz, DMSO-d6) δ 3.09 (s, 1H), 4.11-4.37 (m, 5H), 4.63 (t, *J* = 5.2 Hz, 1H), 5.98 (d, *J* = 5.2 Hz, 1H), 7.63 (s, 2H), 8.35 (s, 1H), 8.40 (s, 1H), 8.42-8.51 (m, 1H); ^{13}C NMR (125 MHz, MeOD): δ 31.5, 69.6, 71.8, 73.7, 75.9, 79.5, 83.9, 90.1, 120.8, 142.0, 149.6, 150.4, 153.2, 161.5; HRMS calcd for $C_{13}H_{16}N_6O_6S$ $[M + H]^+$: 385.09; Found: *m/z* 385.0937

Synthesis of ABP A1

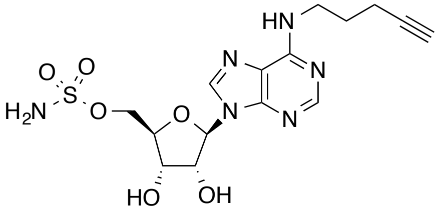

White solid; Yield = 28.8%, purified by HPLC; ^1H NMR (500 MHz, MeOD) δ 1.99 (m, 2H), 2.33 (s, 1H), 2.39 (m, 2H), 3.66-3.74 (m, 2H), 4.33-4.47 (m, 4H), 4.67 (t, *J* = 5.1 Hz, 1H), 6.13 (d, *J* = 5.1 Hz, 1H), 8.38 (s, 1H), 8.43 (s, 1H); ^1H NMR (500 MHz, DMSO-d6) δ 3.09 (s, 1H), 4.11-4.37 (m, 5H), 4.63 (t, *J* = 5.2 Hz, 1H), 5.98 (d, *J* =

5.2 Hz, 1H), 7.63 (s, 2H), 8.35 (s, 1H), 8.40 (s, 1H), 8.42-8.51 (m, 1H); ^{13}C NMR (125 MHz, MeOD): δ 16.5, 28.6, 41.8, 69.6, 70.7, 71.8, 75.9, 83.6, 84.0, 90.2 120.8, 142.2, 148.3, 148.9, 152.4, 162.1; HRMS calcd for $C_{15}H_{20}N_6O_6S$ $[M + H]^+$: 413.12; Found: *m/z* 413.1247

Synthesis of ABP A2

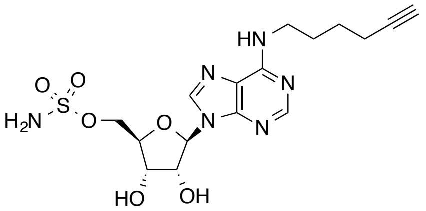

White solid; Yield = 51.6% Purified by HPLC; ^{1}H NMR (500 MHz, MeOD) δ 1.69 (m, 2H), 1.91 (m, 2H), 2.29 (m, 3H), 3.62 (t, *J* = 7.4 Hz, 2H), 4.33-4.43 (m, 4H), 4.68 (t, *J* = 5.1 Hz, 1H), 6.15 (d, *J* = 5.1 Hz, 1H), 8.33-8.57 (m, 2H); ^{1}H NMR (500 MHz, DMSO-d6) δ 3.09 (s, 1H), 4.11-4.37 (m, 5H), 4.63 (t, *J* = 5.2 Hz, 1H), 5.98 (d, *J* = 5.2 Hz, 1H), 7.63 (s, 2H), 8.35 (s, 1H), 8.40 (s, 1H), 8.42-8.51 (m, 1H); ^{13}C NMR (125 MHz, MeOD): δ 18.7, 26.8, 28.7, 42.7, 69.6, 70.2, 71.8, 75.9, 84.1, 84.4, 90.3 120.7, 142.6, 146.2, 147.0, 148.8, 151.4, 162.2; HRMS calcd for $C_{16}H_{22}N_6O_6S$ $[M + H]^+$: 427.13; Found: *m/z* 427.1403

Synthesis of ABP A3

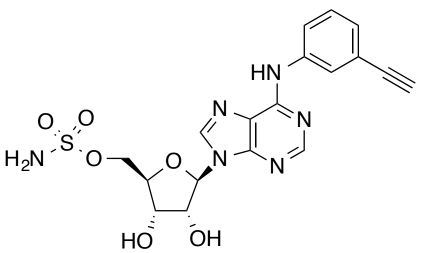

White solid; Yield = 42%, ^{1}H NMR (500 MHz, DMSO-d_6) δ 8.54 (s, 1H), 8.49 (s, 1H), 8.21 (d, J = 6.8, 1H), 8.03 – 7.95 (m, 1H), 7.62 (s, 1H), 7.36 (t, J = 8.0 Hz, 1H), 7.17 (d, J = 7.8, 1H), 6.04 (d, J = 5.1 Hz, 1H), 4.69 (t, J = 5.2 Hz, 1H), 4.36 – 4.19 (m, 4H), 4.18 (s, 1H). ^{13}C NMR (126 MHz, DMSO) δ 152.52, 152.24, 150.09, 141.13, 140.21, 129.34, 126.35, 123.79, 122.22, 121.74, 120.68, 88.22, 84.21, 82.09, 80.89, 73.49, 70.71, 69.14. HRMS calcd for $C_{18}H_{18}N_6O_6S$ [M + H]$^+$: 447.10; Found: *m/z* 447.1082

Synthesis of compound 4

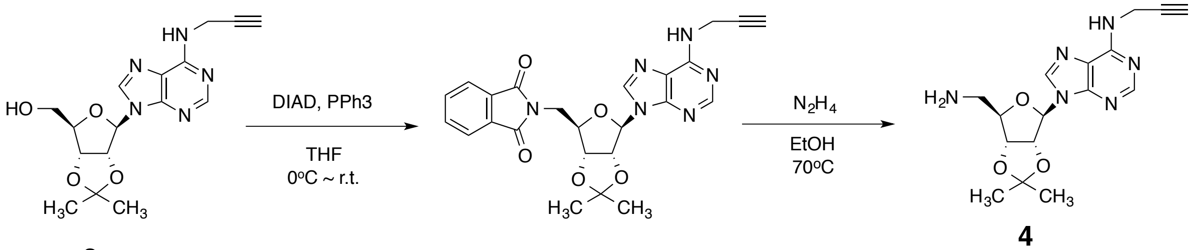

2a **4** 2 step yield = 80 %

To a 50 ml RBF charged with **compound 2a** (600 mg, 1.737 mmol) and phthalimide (306. 6 mg, 2.084 mmol, 1.2 equiv.) dissolved in 12 ml THF, was added triphenylphosphine (59 2 mg, 2.259 mmol, 1.3 equiv.). The reaction mixture was stirred until it became homogene ous. Then the reaction mixture was cooled down to 0 °C, followed by drop-wise addition of diisopropyl azodicarboxylate (513 μl, 2.61 mmol, 1.5 equiv.) in 3 ml THF solution. The reaction mixture was stirred at r.t. for 4 hrs. The crude mixture was briefly purified using a short column chromatography (EtOAc : n-hexane = 1:1) and redissolved in 8 ml EtOH. The reaction mixture was heated to 70 °C, then added by hydrazine (545 μl, 17.37 mmol, 10 equiv). Within 30 min, appearance of while precipitate was monitored and the reaction was completed when detected by TLC. After evaporating the EtOH in reduced pressure, the neat mixture was redissolved in H_2O. Glacier acetic acid was added to the s olution until pH became 4.0. At this condition, the white precipitant was filtered. The filtrat e was cooled down to 0 °C, then added by 5N NaOH until the pH became 11.0. The basi

c aqueous solution was extracted with CH_2Cl_2 three times. Gathered organic layer was dried over $MgSO_4$, filtered, and purified by with silica gel flash column chromatography (MeOH:CH_2Cl_2, gradient 0 → 15% MeOH). to provide **compound 4** (477 mg, 80%) as a sticky, lemony liquid. TLC R_f = 0.15 (MeOH:CH_2Cl_2 = 1:10); ^{1}H NMR (500 MHz, Methanol-*d4*) δ 8.32 (s, 1H), 8.27 (s, 1H), 6.17 (d, *J* = 3.0 Hz, 1H), 5.50 (dd, *J* = 6.4, 3.0 Hz, 1H), 5.04 (dd, *J* = 6.4, 3.3 Hz, 1H), 4.41 (brs, 2H), 4.26 (td, *J* = 5.8, 3.4 Hz, 1H), 2.93 (d, *J* = 5.8 Hz, 2H), 2.64 (t, *J* = 2.5 Hz, 1H), 1.62 (s, 3H), 1.40 (s, 3H). ^{13}C NMR (126 MHz, MeOD) δ 155.53, 153.89, 141.85, 121.30, 115.66, 91.65, 88.33, 84.92, 83.28, 81.19, 72.23, 44.54, 30.40, 27.52, 25.58. MS calcd for $C_{16}H_{20}N_6O_3$ $[M + H]^+$: 345.16; Found: *m/z* 344.88

Synthesis of compound 6

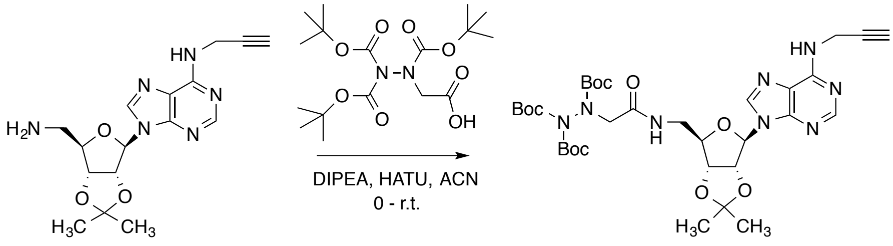

To a flask containing Tri-Boc-hydrazinoacetic acid (62.5 mg, 0.16 mmol, 1.1 equiv.) and ACN (1.5 ml), was added HATU (66 mg, 0.18 mmol, 1.2 equiv.) at 0 °C, followed by addition of compound 4 (50 mg, 0.15 mmol) and diisopropylethylamine (28 μl, 0.16 mmol, 1.1 equiv.). The reaction was completed in 30 min when monitored by TLC. ACN was evaporated under reduced pressure. Then, the reaction mixture was redissolved in CH_2Cl_2 and saturated NH_4Cl solution. The organic compound was extracted three times with CH_2Cl_2. The gathered organic layer was dried over $MgSO_4$ and filtered. The filtrate was concentrated under the reduced pressure and the residue was purified with silica gel flash column chromato

graphy ($MeOH:CH_2Cl_2$ gradient 0 → 7 % MeOH) to provide **compound 6** (88.6 mg, 85%) as a white solid. TLC R_f = 0.65 ($MeOH:CH_2Cl_2$ = 1:10); ^1H NMR (500 MHz, Chloroform-*d*) δ 8.44 (s, 1H), 7.97 (s, 1H), 6.40 (d, *J* = 18.8 Hz, 2H), 6.04 (s, 1H), 5.36 (dd, *J* = 6.4, 2.9 Hz, 1H), 4.94 (dd, *J* = 6.4, 3.2 Hz, 1H), 4.47 (brs, 2H), 4.39 – 4.32 (m, 2H), 3.68 – 3.45 (m, 2H), 2.28 (t, J = 2.5 Hz, 1H), 1.51 (s, 3H), 1.48 – 1.46 (m, 27H). ^{13}C NMR (126 MHz, CDCl3) δ 169.15, 168.80, 165.72, 154.02, 153.89, 153.27, 153.11, 151.27, 151.02, 150.93, 139.59, 139.42, 114.60, 114.49, 90.77, 90.46, 85.54, 84.98, 84.95, 84.91, 84.88, 84.81, 84.14, 83.96, 83.65, 83.32, 82.66, 82.42, 82.00, 80.04, 71.55, 56.42, 54.74, 41.20, 38.60, 30.27, 27.95, 27.17, 25.40.

Synthesis of compound 7

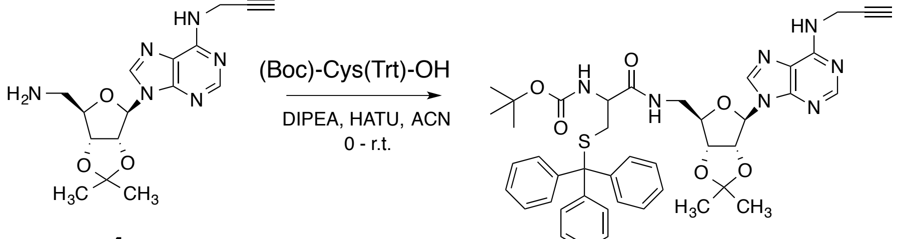

4 7

Compound 7 was prepared as white solide following the same procedure as the **compound 6** preparation. TLC showed > 95% conversion of starting material into the desired product. However, the correct yield was not measured due to residual amount of urea which was coeluted from the silica column chromatography, TLC R_f = 0.73 ($MeOH:CH_2Cl_2$ = 1:10); ^1H NMR (500 MHz, Chloroform-*d*) δ 8.98 – 8.88 (m, 1H), 8.27 (s, 1H), 7.76 (s, 1H), 7.31 – 7.24 (m, 6H), 7.02 (t, *J* = 7.7 Hz, 6H), 6.92 (t, *J* = 7.3 Hz, 3H), 6.33 – 6.17 (m, 1H), 5.65 – 5.59 (m, 2H), 4.90 – 4.79 (m, 1H), 4.58 (d, *J* = 5.9 Hz, 1H), 4.42 (brs, 2H), 4.26 – 4.13 (m, 1H), 3.09 (d, *J* = 14.5 Hz, 1H), 2.55 – 2.38 (m, 2H), 2.23 (t, *J* = 2.5 Hz, 1H), 1.52 (s, 3H), 1.39 (s, 12H). ^{13}C NMR (126 MHz, CDCl3) δ 170.85, 165.76, 155.03,

152.81, 144.19, 140.06, 129.31, 127.76, 126.48, 114.48, 93.49, 82.74, 81.62, 79.88, 71.65, 6 6.55, 52.80, 41.35, 38.63, 34.83, 28.46, 27.50, 24.76. MS calcd for $C_{43}H_{47}N_7O_6S$ $[M + H]^+$: 790.33; Found: m/z 790.22

Synthesis of Boc-Sulfamyl Chloride[1]

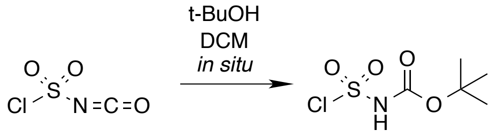

t-BuOH (714 μl, 7.46 mmol, 1.3 equiv.) and CH_2Cl_2 (5.7 ml) in a RBF is stirred at 0 °C. To the mixture, chlorosulfonyl isocyanate (500 μl, 5.74 mmol, 1 equiv.) was added drop-wisely over the course of 10 min. 5 min later, the reaction mixture was removed from the ice bath and warmed to r.t. Then, the solvent was evaporated until the total volume of the crude mixture became 2 ml. When the mixture was placed back into the ice bath, white crystals appeared, which was then filtered and washed with n-hexane. The white crystal was stored at -20 °C until use.

Synthesis of compound 8

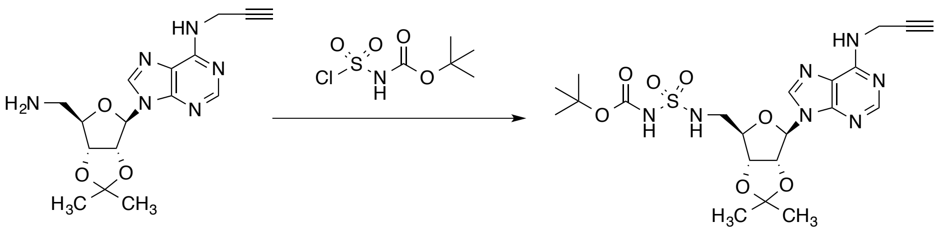

4 **8**

To **compound 4** (50 mg, 0.15 mmol, 1 equiv.) dissolved in CH_2Cl_2 (2ml), was added Boc-sulfamyl chloride (47 mg, 0.22 mmol, 1.5 equiv.) and TEA (61 μl, 0.44 mmol, 3 equiv.)

at 0 °C. The reaction was completed in 30 min when monitored by TLC. The reaction mix ture was filtered. With the filtrate, work-up process was followed (CH_2Cl_2/H_2O). The water layer was extracted twice using EtOAc. Gathered organic layer was dried over $MgSO_4$, and filtered. The filtrate was concentrated under the reduced pressure and the residue was purif ied with silica gel flash column chromatography ($MeOH:CH_2Cl_2$ gradient 0 → 5 % MeOH) to provide **compound 8.** However, ^1H NMR showed an unidentified byproduct, the R_f of w hich was exactly the same as that of **compound 8**. Therefore, this byproduct was completel y separated after deprotection of acetonide protecting group in the following step, using HP LC.

Synthesis of compound 9

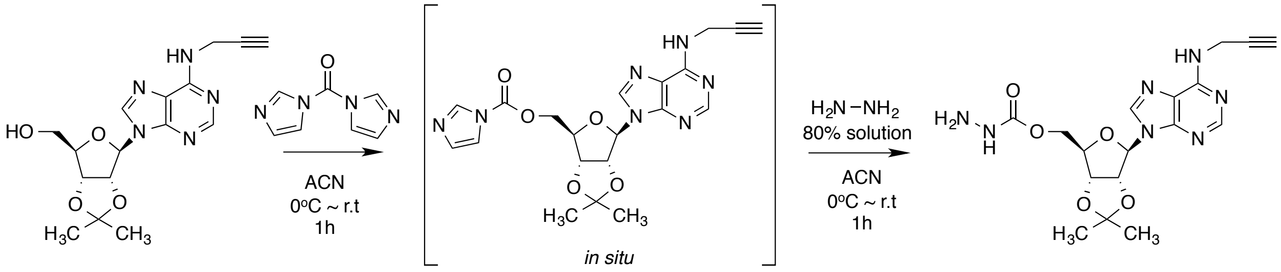

2a **5** **9**, 92%

To 1,1'-carbonyldiimidazole (56.3 mg, 0.35 mmol, 1.2 equiv.) in 0.7 ml of ACN, was add ed **compound 2a** (100 mg, 0.29 mmol, 1 equiv.) dissolved in ACN (0.7 ml) at 0 °C. The reaction mixture was further stirred at r.t. under N_2. In 1 hour, the starting material was c ompletely converted into the desired product when monitored by TLC. ACN was evaporated under reduced pressure. Then, the resulting residue was dissolved in H_2O and extracted wi th CH_2Cl_2 three times in order to remove free imidazole. Combined organic layers were dri ed with $MgSO_4$ and filtered. The concentrated reaction mixture was redissolved in 1 ml of

ACN, to which hydrazine solution (80%, 28 l, 0.58 mmol, 2 equiv.) dissolved in ACN (1 ml) was added drop-wise at 0 °C. The reaction was completed in 1 hour when monitored by TLC. Accordingly, ACN was evaporated under reduced pressure. The resulting residue w as dissolved in H_2O and extracted with CH_2Cl_2 (x 3). Combined organic layers were dried with $MgSO_4$ and filtered. The filtrate was concentrated under the reduced pressure and the residue was purified with silica gel flash column chromatography (EtOAc:n-hexane = 1:1, to which MeOH was added gradually, 2 → 20%) to provide **compound 9** (107.1 mg, 91.7%) as a white solid. TLC R_f = 0.43 (EtOAc:n-hexane:MeOH = 5:5:1); ^{1}H NMR (500 MHz, Chloroform-*d*) δ 8.39 (s, 1H), 7.87 (s, 1H), 6.63 (d, *J* = 13.6 Hz, 2H), 6.07 (d, *J* = 2.0 H z, 1H), 5.46 (dd, *J* = 6.4, 1.9 Hz, 1H), 5.04 (dd, *J* = 6.5, 3.3 Hz, 1H), 4.47 – 4.34 (m, 4 H), 4.22 (dd, *J* = 11.5, 6.4 Hz, 1H), 3.75 (brs, 2H), 2.24 (t, *J* = 2.5 Hz, 1H), 1.58 (s, 3H), 1.36 (s, 3H). ^{13}C NMR (126 MHz, CDCl3) δ 158.14, 154.05, 153.23, 139.61, 120.51, 11 4.55, 91.07, 85.37, 84.13, 81.60, 80.08, 77.30, 77.24, 77.04, 76.98, 76.79, 71.53, 64.96, 50. 68, 30.19, 27.15, 25.38. MS calcd for $C_{17}H_{21}N_7O_5$ $[M + H]^+$: 404.16; Found: *m/z* 403.97

Synthesis of compound 10

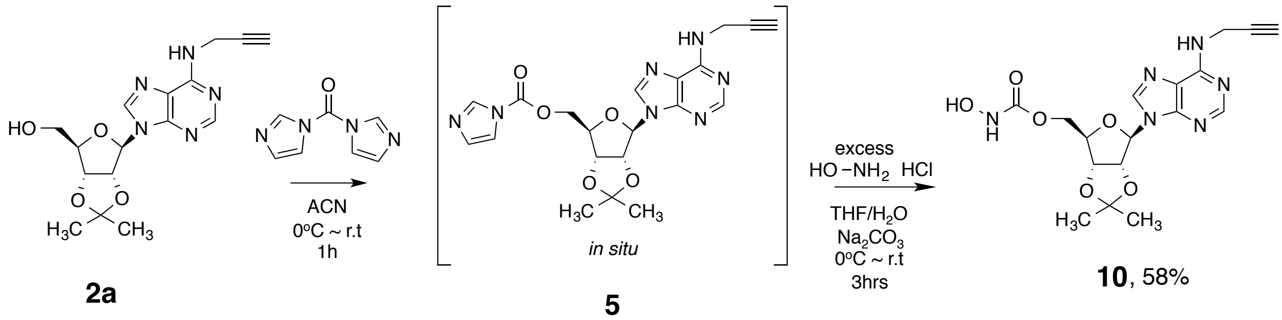

2a **5** **10**, 58%

Compound 5 was prepared from compound 2a (100 mg, 0.29 mmol, 1 equiv.) following th e previous procedure, and redissolved in THF/H_2O (1:1, 2 ml total volume). To the solutio n, was added hydroxylamine hydrochloride salt (150mg, excess) and sodium carbonate (61.4

mg, 0.58 mmol, 2 equiv.) at 0 °C. The reaction mixture was further stirred at r.t. for 3 h rs. Then, THF was evaporated under reduced pressure, followed by addition of saturated am monium chloride. The organic compounds were extracted with CH_2Cl_2 three times, dried wit h $MgSO_4$ and filtered. (^{1}H NMR (500 MHz, Chloroform-*d*) δ 9.61 (brs, 1H), 8.60 (s, 1H) , 8.33 (s, 1H), 8.01 (s, 1H), 6.85 (d, *J* = 6.8 Hz, 1H), 6.09 (s, 1H), 5.25 – 5.19 (m, 1H), 4.93 (dd, *J* = 6.1, 3.1 Hz, 1H), 4.48 – 4.21 (m, 5H), 2.24 – 2.16 (m, 1H), 1.53 (s, 3H), 1.28 (s, 3H). ^{13}C NMR (126 MHz, CDCl3) δ 207.17, 158.50, 153.70, 153.33, 139.47, 119 .46, 114.65, 90.44, 84.66, 84.24, 80.99, 80.26, 71.35, 64.98, 53.46, 30.93, 30.25, 27.12, 25. 28. MS calcd for $C_{17}H_{20}N_6O_6$ $[M + H]^+$: 405.14; Found: *m/z* 405.16

Synthesis of nucleophile analogs: global deprotection of acid labile protecting groups

A flask containing one of **compound 6, 7, 8, 9, 10** (50 mg) in CH_2Cl_2 (300 μL), was tre ated with a solution of TFA in H_2O (4:1 v/v, total volume 2 mL) at 0 ºC. The reaction m ixture was stirred at r.t. for 1 hr. The reaction progress was monitored by thin layer chrom atography. CH2Cl2, TFA and ddH2O were removed under high vacuum (oil pump, overnig ht). The remaining residue was purified by reverse phase high performance liquid chromato graphy (solvent A: 95% ddH_2O, 5% acetonitrile, 0.1% TFA, solvent B: 95% acetonitrile, 5 % ddH_2O, 0.1% TFA. Gradient method 0 to 100%) Collected fractions were lyophilized to yield the corresponding ABP analog.

ABP N1

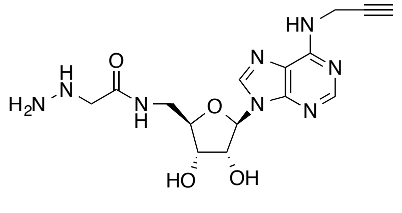

A modification of the aforementioned procedure was applied for the synthesis of ABP N1. Specifically, 2 equiv. of hydrazine was added to the TFA/H_2O (4:1) mixture, in order to sc avenge the acetone liberated from acetonide. Addition of free hydrazine was critical for the high yield, because in the absence of hydrazine, acetone reacted with the desired product, resulting in hydrazone formation. ^1H NMR (500 MHz, Methanol-d_4) δ 8.40 (s, J = 3.1 Hz, 1H), 8.33 (s, J = 7.2 Hz, 1H), 5.98 (d, J = 5.4 Hz, 1H), 4.80 (t, J = 5.5 Hz, 1H), 4.45 – 4.35 (brs, 2H), 4.29 – 4.23 (m, 1H), 4.22 – 4.19 (m, 1H), 3.81 – 3.76 (m, 1H), 3.74 (s, 2H), 3.58 (dd, J = 14.1, 4.0 Hz, 1H), 3.37 (s, 3H), 2.70 (s, 1H). ^{13}C NMR (126 MHz, MeOD) δ 164.13, 89.55, 83.52, 78.83, 73.43, 71.53, 50.35, 40.91 HRMS calcd for $C_{15}H_{20}N_8O_4$ $[M + H]^+$: 377.16; Found: *m/z* 377.1686

ABP N2

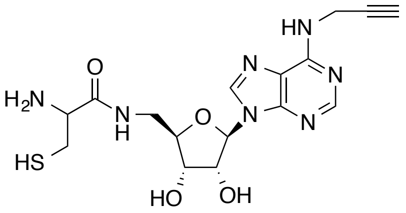

A modification of the aforementioned procedure was applied for the synthesis of **ABP N2**. Specifically, 4 equiv. of Et_3SiH was added to the TFA/H_2O (4:1) mixture, in order to scav enge trityl cation. ^1H NMR (500 MHz, Methanol-d_4) δ 8.43 (s, 2H), 6.02 (d, J = 5.5 Hz, 1H), 4.84 (t, J = 5.5 Hz, 1H), 4.54 – 4.35 (brs, 2H), 4.27 (dd, J = 5.4, 4.0 Hz, 1H), 4.21 (dt, J = 7.9, 4.1 Hz, 1H), 4.07 (t, J = 5.9 Hz, 1H), 3.84 (dd, J = 14.0, 7.0 Hz, 1H), 3.59 (dd, J = 14.2, 4.0 Hz, 1H), 3.01 (qd, J = 14.6, 5.9 Hz, 2H), 2.76 (s, 1H). ^{13}C NMR (126 MHz, MeOD) δ 168.72, 162.20, 161.91, 142.87, 118.78, 116.47, 90.80, 84.63, 79.89, 74.91, 72.93, 56.20, 42.94, 42.82, 31.2, 26.32 HRMS calcd for $C_{16}H_{21}N_7O_4S$ $[M + H]^+$: 408.14; Found: *m/z* 408.1448

ABP N3

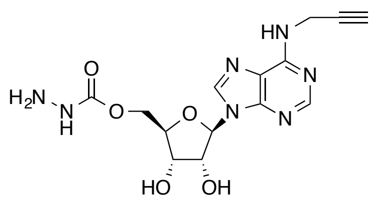

ABP N3 was prepared following the same procedure for **ABP N1**. ^{1}H NMR (500 MHz, D MSO-d_6) δ 8.43 (s, 1H), 8.34 (s, 1H), 5.98 (d, J = 5.6 Hz, 1H), 4.70 (t, J = 5.4 Hz, 1H), 4.48 – 4.34 (m, 2H), 4.34 – 4.21 (m, 3H), 4.15 (dt, J = 6.4, 3.9 Hz, 1H), 3.07 (s, 1H). ^{13}C NMR (126 MHz, DMSO) δ 159.07, 158.78, 153.96, 140.61, 119.79, 117.47, 115.14, 11 2.82, 87.86, 82.33, 82.07, 73.44, 72.95, 70.84, 66.55, 55.50, 49.04, 29.67 HRMS calcd for $C_{14}H_{17}N_7O_5$ $[M + H]^+$: 364.13; Found: *m/z* 364.1367

ABP N4

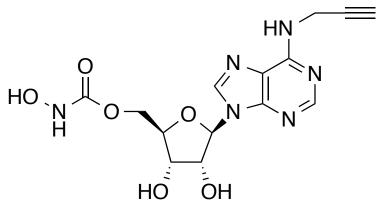

ABP N4 was prepared following the same procedure for **ABP N1**, except that instead of a dding free hydrazine, hydroxyl amine (2 equiv.) was added in order to prevent oxim format ion between the desired product and acetone. ^{1}H NMR (500 MHz, Methanol-d_4) δ 8.56 – 8 .47 (m, 1H), 8.46 – 8.43 (m, 1H), 6.19 – 6.06 (m, 1H), 4.74 – 4.65 (m, 1H), 4.55 – 4.24 (m, 6H), 2.82 (q, J = 2.3 Hz, 1H) ^{13}C NMR (126 MHz, MeOD) δ 160.55, 160.26, 158.7 6, 147.85, 141.21, 119.17, 117.17, 114.86, 88.70, 83.09, 77.70, 74.46, 72.77, 70.50, 64.08, 53.80, 30.38 HRMS calcd for $C_{14}H_{16}N_6O_6$ $[M + H]^+$: 365.11; Found: *m/z* 365.1199

ABP N5

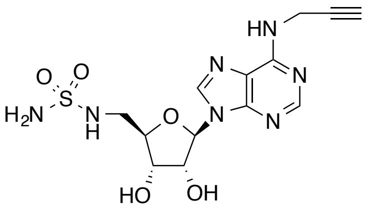

ABP N5 was prepared following the general deprotection procedure. ^{1}H NMR (500 MHz, Methanol-d_4) δ 8.48 – 8.29 (m, 2H), 5.97 (d, J = 5.6 Hz, 1H), 4.85 (t, J = 5.9 Hz, 1H), 4.56 – 4.22 (m, 4H), 3.48 – 3.38 (m, 2H), 2.76 (s, 1H), ^{13}C NMR (126 MHz, MeOD) δ 141.84, 90.05, 84.65, 78.39, 73.25, 71.99, 71.49, 44.61, 28.74 HRMS calcd for $C_{13}H_{17}N_7O_5$ S $[M + H]^+$: 384.10; Found: *m/z* 384.1082

III. Bio-experimental Protocols

General Information

Human recombinant UBE1, SAE1/SAE2, UbcH5a, Ubc9, SUMO1 proteins were purchased from R&D Systems. Ubiquitin (From bovine erythrocytes) and MG132 were purchased from from Sigma-Aldrich. Purchased proteins were used without further purification. Compound 1 was generously gifted from Peter Foote in Statsyuk group. In-gel fluorescence imaging was performed on a Typhoon 9600 (GE Healthcare). Proteins on polyacrylamide gels were visualized with Instant*Blue*™ (expedeon) staining solution. For desalting, Zeba Spin Desalting Columns (7K MWCO, Thermo Scientific) were used.

Tag-Ub-MesNa expression and purification

3xFlag 6xHis tagged ubiquitin inserted in pTYB1 plasmid was transformed into Rosetta DE3 cells (EMDmillipore). 1L LB madia containing 100μg/ml ampicillin was inoculated with 50 ml overnight culture and incubated at 37 ºC until OD reached ~1.2. The cell culture media was cooled down to 15 ºC on ice. Then, IPTG (0.1 mM final concentration) was added to the cell culture media, followed by 18 hours incubation at the same temperature. Cells were then harvested and lysed by sonication in phosphate buffered saline (PBS) with protease inhibitors (Complete Mini Protease Inhibitor Cocktail, Roche). The supernatant was incubated with Chitin beads (Pierce Biotechnology) for 1hour at 30 ºC with agitation. The beads were washed three times with KW buffer (HEPES 20mM, NaOAc 50mM, NaCl 75 mM, pH6.5) and incubated with 100 mM β-mercaptoethane sulfonic acid (MesNa) overnight at 30 ºC, 150 rpm to cleave the thioester bond and elute the desired tag-Ub-MesNa. The gathered eluent was concentrated (Amicon Ultra spin filter 3K, Millipore), purified by HPLC, and lyophilized.

Chemical reactivity test of ABPs with Ub-MesNa using Mass Analysis

3xFlag-6xHis-Ub-MesNa (1 mM) in buffer containing HEPES (25 mM, pH 7.6), NaCl (50 mM), and $MgCl_2$ (4 mM) was incubated with each ABP1 analog (1 mM) for 2 hours (20 μl total volume). The reaction mixture was filtered through Zeba Spin Desalting Column to remove excessive amount of **ABP**s. In the case of Figure 1S, TCEP (1 mM) was incubated with the reaction mixture for 15 min in order to reduce the di-thiobond. The filtrate was immediately subjected to MS analysis (LC-TOF: Agilent 6210A).

Cell viability Assay

MM.1S cells and A549 cells were seeded in 96-well plates (90 μl, 10^4 cells per well) and incubated overnight at 37 °C. Cells were treated with the indicated concentrations of **ABP A3,** 0.2 % DMSO or RPMI media (10 μl each), and incubated for 48 hours. The growth inhibitory effect of **ABP A3** was analyzed by measuring luminescent signal of each well after treating the cells with 100 μl of CellTiter-Glo (Promega) for 10 min. IC50 was obtained using Prism (GraphPad) software.

Cell Cycle Analysis

A549 cells logarithmically growing in T-75 flask were treated with **ABP A3** (0.8 or 2.5 μM) or DMSO, and incubated for 12, 24, 48 hours. RPMI media was removed, and cells were washed with DPBS followed by trypsin treatment. Collected cells (~ 3 x 10^6) were washed with DPBS (2 x 10 ml), and fixed with EtOH (80%) at 4 °C for 2 hours. 1 x 10^6 cells were stained with Propidium iodide/RNase staining solution (500 μl, Cell Signaling) for 20 min at dark, 37 °C. 5 x 10^4 cells were analyzed for cell cycle profile using flow cytometry (BD™ LSR II, BD biosciences) and processed with FlowJo X software.

Immunoblotting

A549 cells were cultured with small molecule inhibitors or DMSO in 6-well plates for the indicated time duration. Cells were washed with PBS, and lysed by direct addition of 2 x *Laemmli loading buffer* (200 μl, 2 x *Laemmli loading buffer*: 100 mM Tris-Cl, 4% SDS, 0.2% bromophenol blue, 20% glycerol, 200 mM DTT, pH 6.8). Collected cell lysates were boiled for 5 min at 95 °C, and subjected for SDS-PAGE, transferred to nitrocellulose (0.45 μm, ThermoScientific) or PVDF membrane (0.2 μm, Bio-Rad) in transfer buffer (25 mM Tris, 192 mM glycine, 10% methanol). The membrane was washed with TBS for 5 min, blocked by nonfat milk for one hour (5% in TBST), and treated with Ub, SUMO1, Nedd8, p21, LC3A/B, Cleaved PARP (Cell Signaling), Ufm1, p53 and β-actin (Millipore), IGS15 (R&D systems), and SUMO2 (Invitrogen) overnight. The membrane was washed with TBST 3×5 min, and incubated with the HRP conjugated secondary antibody (rabbit IgG or mouse IgG, Bio-rad) for one hour, followed by wash with TBST 3×5min. Immunoblots were developed with ECL reagent (Bio-rad) and imaged using ChemiDoc^{TM} XRS^{+}, Bio-rad).

Immunostaining

1.5 x 10^5 A549 cells were seeded on 6-well plate containing 3 x cover slips. After over night incubation, cells were washed with DPBS and incubated with fresh RPMI media containing ABP A3 (2.5 μM), MG132 (5 μM) or DMSO (0.025%). After each given incubation time, cells on the cover slips were washed with DPBS and fixed with freshly prepared paraformaldehyde (4%) for 15 min at r.t. The cells were washed with DPBS three times then further fixed (permeabilized) with ice-cold methanol (100%) for 10 min at -20ºC in order to detect LC3 on autophagosome lipid membrane. The cells were washed with DPBS 5min x 3, blocked with DPBS containing 1% BSA and 0.3% triton X-100 for 1hr

at r.t. Following the wash (DPBS, 5min x 3), cells were incubated with primary antibody at dark, r.t. overnight, washed again with DPBS (5min x 3), and incubated with secondary antibodies (Anti-Rabbit IgG-Alexa Fluor 594, Anti-mouse IgG-Alexa Fluor 488, Cell Signaling Technology) for 2hrs, r.t. Finally, cells were washed again (DPBS, 5min x 3) and a drop of Prolong gold antifade reagent with DAPI (Cell Signaling Technology) was applied before mounting on a slide glass. 24 hrs later, the specimen was analyzed by Leica SP5 II Laser Scanning Confocal Microscope.

AnnexinV-PI staining and Flow Cytometry (FC) analysis

~1.25 x 10^5 A549 cells were seeded on a 6-well plate, and incubated overnight. The cells were washed and incubated with fresh RPMI media containing **ABP A3** (2.5 μM) or DMSO (0.025%) for 24 hrs. Cells were washed with DPBS and trypsinized. After another wash, 1 x 10^6 cells were collected and stained with AlexaFluor488-AnnexinV and PI solution (*Life*technologies) at r.t. for 15 min at dark. The cells were immediately analyzed using flow cytometry (BD™ LSR II, BD biosciences) and processed with FlowJo X software.

ISG15 induction by IFN-β and ABP A3 treatment

A549 cells were grown in RPMI medium (*life*technologies) supplemented with 10% fetal bovine serum in 6-well plates. When cells reached ~30% confluency, they were washed with Dulbecco's Phosphate-Buffered Saline (DPBS) 2×3 mL and treated with RPMI medium containing 1000 U/mL of IFN-β and 10% FBS. The cells were incubated for 24 hrs at 37 ºC to induce the expression of ISG15. Cells in the each well were treated with the indicated concentrations of inhibitors and incubated for additional 1, 4, 8 hours. The growth

media was removed and the cells were washed with DPBS (3 mL). Cells were then lysed by direct addition of 2 x 2 x *Laemmli loading buffer* (200 µl, 2 x *Laemmli loading buffer*: 100 mM Tris-Cl, 4% SDS, 0.2% bromophenol blue, 20% glycerol, 200 mM DTT, pH 6.8), and boiled at 95 ºC for 5 min. The general immunoblotting process was followed.

Concentration dependence on the ubiquitin●ABP formation *in vitro*

Ub (50 µM), ATP (50 µM), and increasing concentration of each **ABP** in buffer containing HEPES (25 mM, pH 7.6), NaCl (50 mM), and $MgCl_2$ (4 mM) were treated with UBE1 (0.5 µM) at room temperature (10 µL total volume) for 2 hrs. Each reaction mixture was filtered through Zeba Spin Desalting Column to remove excessive amount of **ABP**. Collected filtrates were treated with 1 µl of 10% SDS followed by 4 µL of click reaction mixture: $CuSO_4$ (final conc. 1 mM), TBTA (final conc. 100 µM), sodium ascorbate (final conc. 1 mM), and Azide-Fluor-585 (final conc. 100 µM). The reaction mixture was incubated for 30 min at r.t., followed by the addition of 3 µL of 6× *Laemmli loading buffer*, and resolved by 18% SDS-PAGE. The bottom of the gel was cut to eliminate the excessive amount of Azide-Fluor-585. The acrylamide gel was kept in 50% ddH_2O, 40% EtOH and 10% Acetic acid solution until it was subjected to the in-gel scanning fluorescence imaging (Typhoon 9600, GE Healthcare). Long storage (more than two hours) in the storage solution led to decreased fluorescence intensity of the signals. The proteins in the gel were then visualized with Instant *Blue*™.

In-gel fluorescent detection of UBL●ABP in live A549 cells

A549 cells were grown in RPMI medium (*life*technologies) supplemented with 10% fetal bovine serum in 6-well plates. Confluent A549 cells (9.6 cm^2 per condition in 6-well plates)

were washed with Dulbecco's Phosphate-Buffered Saline (DPBS, 3 mL) and the growth medium was replaced with serum-free RPMI medium containing **ABP1 analogs**. After 1 hr incubation time at 37 ºC, cells were washed with DPBS (3 mL). 200 μL of lysis buffer containing Tris HCl (25 mM, pH 7.6), NaCl (150 mM), 1% NP40, 1% sodium deoxycholate, 0.1 % SDS, and protease inhibitor cocktail for mammalian cells (Sigma-Aldrich, 1:100v/v) was added to each well of the 6-well plates, and cells were lysed on ice for 10 min. The complete lysis of the cells was monitored using Carl Zeiss Primo Vert inverted microscope. Cellular debris were cleared by centrifugation (21,000×g) for 15 min at 4 ºC. Each cell lysate was then concentrated with a centrifugal filter (Amicon Ultra Centrifugal Filters 3K, Millipore) to 100μL volume, and the total protein concentration was normalized to 2.0 mg/mL, using Bradford assay. 10 μL of each cell lysate was treated with sodium dodecyl sulfate (final concentration 1%), followed by the incubation with the click chemistry reagents ($CuSO_4$ (final conc. 1 mM), TBTA (final conc. 100 μM), sodium ascorbate (final conc. 1 mM) and Azide-Fluor-585 (final conc. 100 μM) for 30 min. Proteins were resolved by 18% SDS-PAGE, and subjected to in-gel scanning fluorescence imaging to visualize UBL●**ABP** covalent adducts. Total proteins in the gel were then visualized by staining the gel with Instant *Blue*™.

Isolation of ABP1/ABP A3 labeled proteins from A549 cells and MS analysis

A549 cells were grown in RPMI medium supplemented with 10% fetal bovine serum. Confluent cells in T-175 cm^2 flask were washed with Dulbecco's Phosphate-Buffered Saline (DPBS, 20 ml) and treated with serum-free RPMI medium containing **ABP1** (50 μM) or **ABP A3** (50 μM). After one hour of incubation at 37 ºC, the growth media was removed and the cells were washed with DPBS (20 mL). 5 mL of lysis buffer containing Tris HCl

(25 mM, pH 7.6), NaCl (150 mM), 1% NP40, 1% sodium deoxycholate, 0.1% SDS, and protease inhibitor cocktail for mammalian cells (Sigma-Aldrich, 1:100v/v) was added, and cells were lysed on ice for 10 min. Cell lysis was monitored with the microscope (Carl Zeiss Primo Vert inverted microscope). Cell lysates were passed through 21 gauge needle attached to the 10 mL plastic syringe. Cellular debris were cleared by centrifugation (21,000×g) for 15 min at 4 ºC. Each cell lysate was incubated with the click chemistry reagents ($CuSO_4$ (final conc. 1 mM), TBTA (final conc. 100 μM), sodium ascorbate (final conc. 1 mM), and PEG_4 carboxamide-6-azidohexanyl biotin (final conc. 100 μM) for 1 hr at room temperature (6 mL total volume). Cold acetone (24 mL) was added to the reaction mixture then incubated at -20 ºC for 30 min. Protein precipitates were then centrifuged (4,700×g) for 20 min at 4 ºC, and supernatant was removed. Protein pellet was redissolved in 10 ml of HEPES buffer (pH 7.4, 100 mM) containing 0.5% SDS, followed by incubation with 1.0 ml of streptavidin beads (invitrogen) for 1 hr at r.t. The beads were then washed with HEPES buffer 2×10 mL (pH 7.4, 100mM, 0.5% SDS), 2×10 mL of ultrapure water, and with 2×10 mL of HEPES buffer (pH 7.4, 100 mM). Proteins were digested on bead by trypsin or LysC and subjected for MS analysis.

MS anaylsis

The samples were loaded directly onto a 10 cm long, 75 μM reversed phase capillary column (ProteoPep™ II C18, 300 Å, 5 μm size, New Objective, Woburn MA) and separated with a 70 minute gradient from 5% acetonitrile to 100% acetonitrile on a Proxeon Easy n-LC II (Thermo Scientific, San Jose, CA). The peptides were directly eluted into an LTQ Orbitrap Velos mass spectrometer (Thermo Scientific, San Jose, CA) with electrospray ionization at 350 nl/minute flow rate. The mass spectrometer was operated in data dependent mode, and for each MS1 precursor ion scan the ten most

intense ions were selected from fragmentation by CID (collision induced dissociation). The other parameters for mass spectrometry analysis were: resolution of MS1 was set at 60,000, normalized collision energy 35%, activation time 10 ms, isolation width 1.5, and the +1 and +4 and higher charge states were rejected.

The data were processed using PD 1.4(Proteome Discoverer, version 1.4, Thermo Scientific, San Jose, CA) and searched using SEQUEST HT search algorithm as in PD 1.4. The data were searched against H. sapiens reference proteome downloaded from uniprot.org (version 2013_7). The other parameters were as follows: (i) enzyme specificity: trypsin (ii) fixed modification: cysteine carbamidomethylation; (iv) variable modification: methionine oxidation and N-terminal acetylation; (v) precursor mass tolerance was ±10 ppm; and (vi) fragment ion mass tolerance was ±0.8 Da. The PSM (Peptide spectral match) were used for peptide assignments. The peptide identification was considered valid if its corresponding mascot score was equal to or less then the threshold. Protein grouping was enabled in Proteome discoverer and proteins were grouped to satisfy the rule of parsimony. Further, in the final protein list protein identification was considered only valid if supported by at least one unique peptide.

Protein abundance was determined by spectral counting as number of total spectral count assigned to all the protein identification by PD 1.4. In addition to spectral counting, protein abundance was also calculated by summing the total ion intensities for each peptide assignments for a given protein. The extracted ion intensities from MS1 raw data based on precursor mass assigned by SEQUEST HT was done as in PD 1.4

IV. Spectral Data

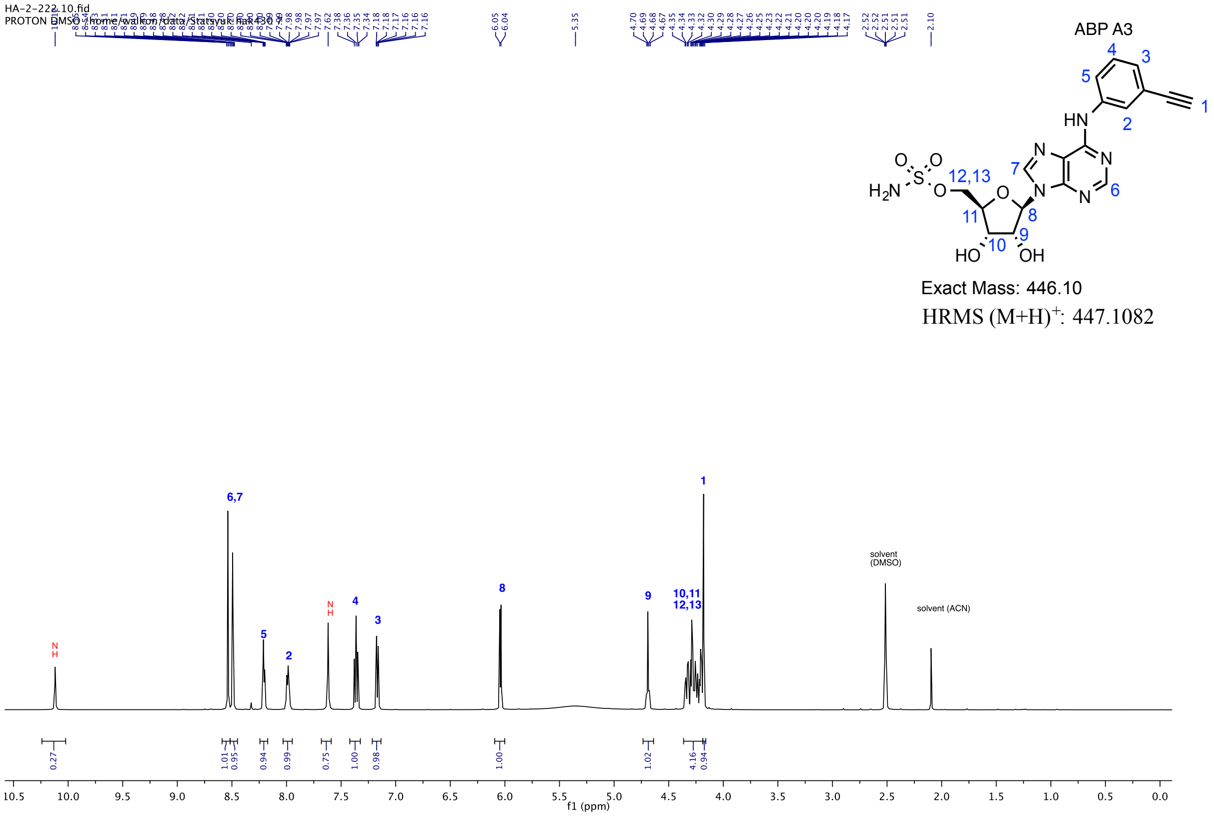

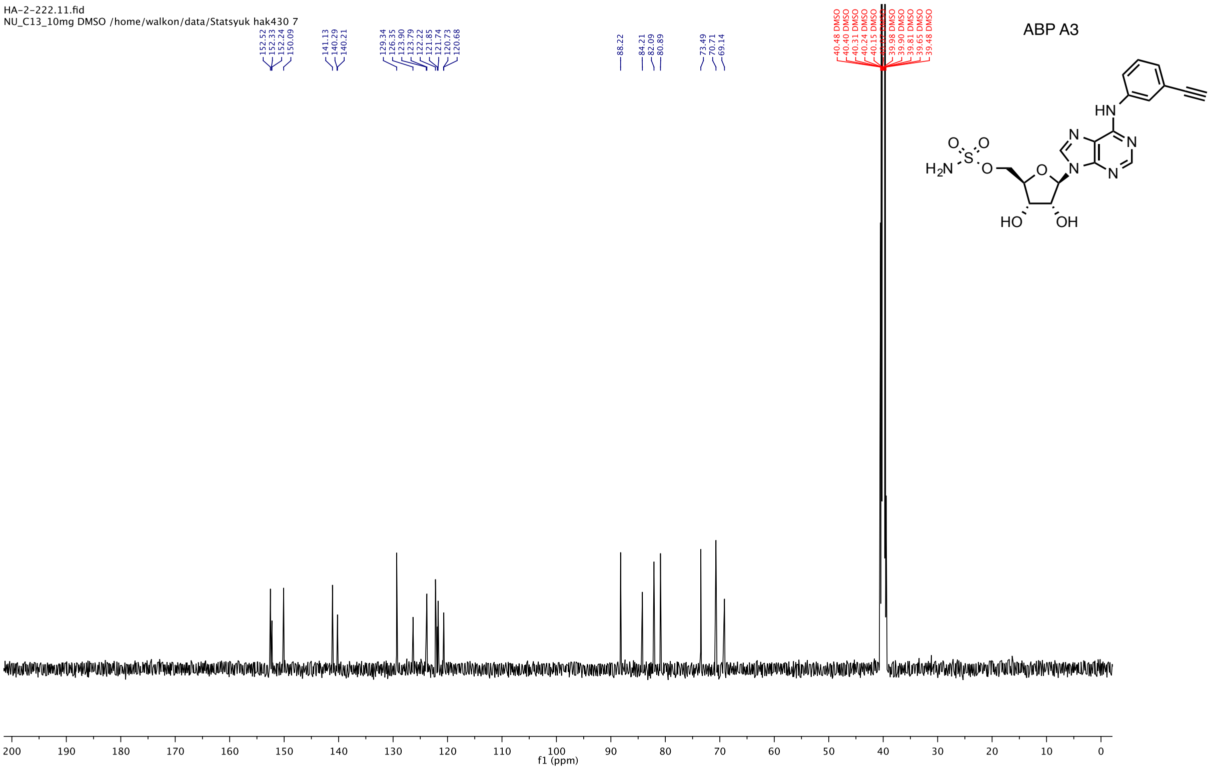

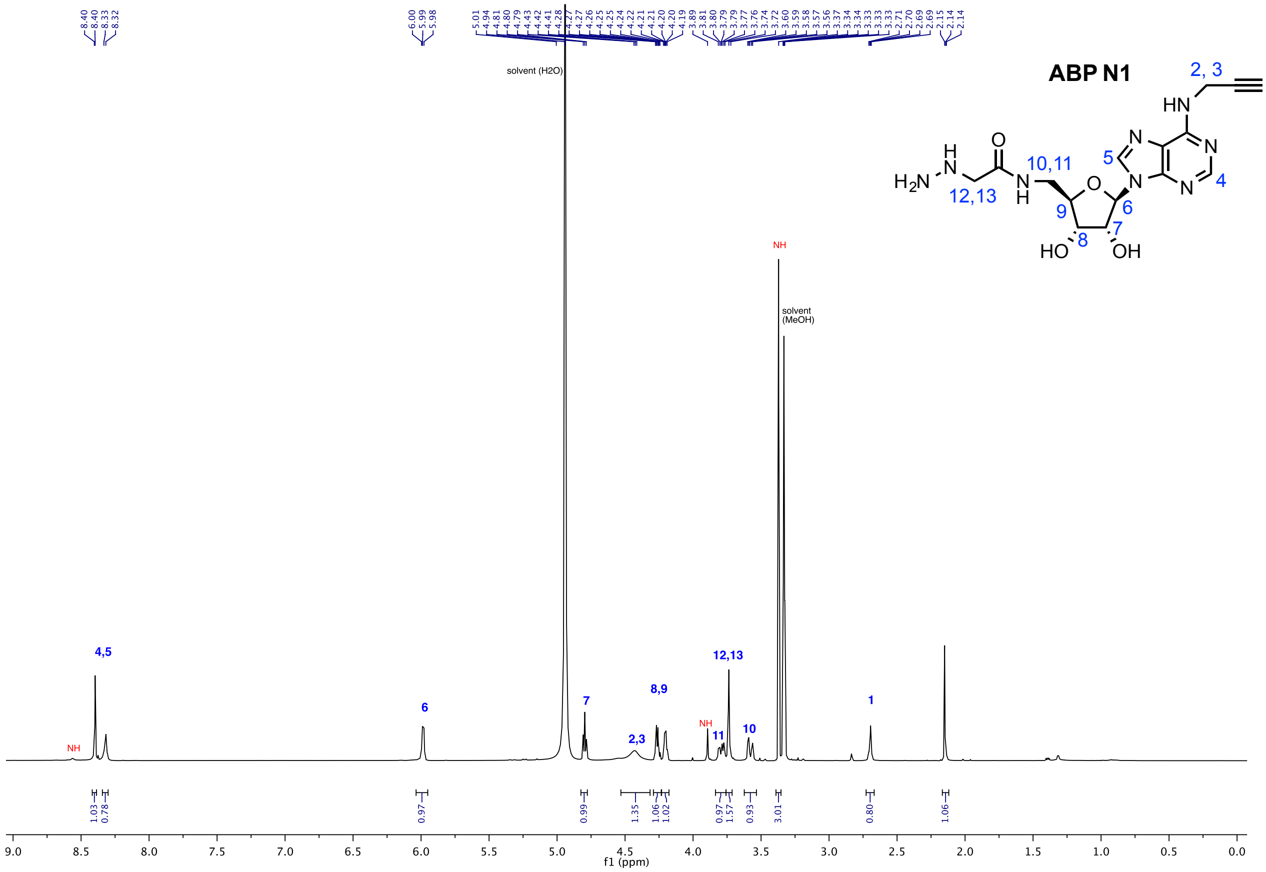
ABP N1
solvent (H2O)
solvent (MeOH)
NH
4,5
6
7
2,3
8,9
11
12,13
10
1
f1 (ppm)

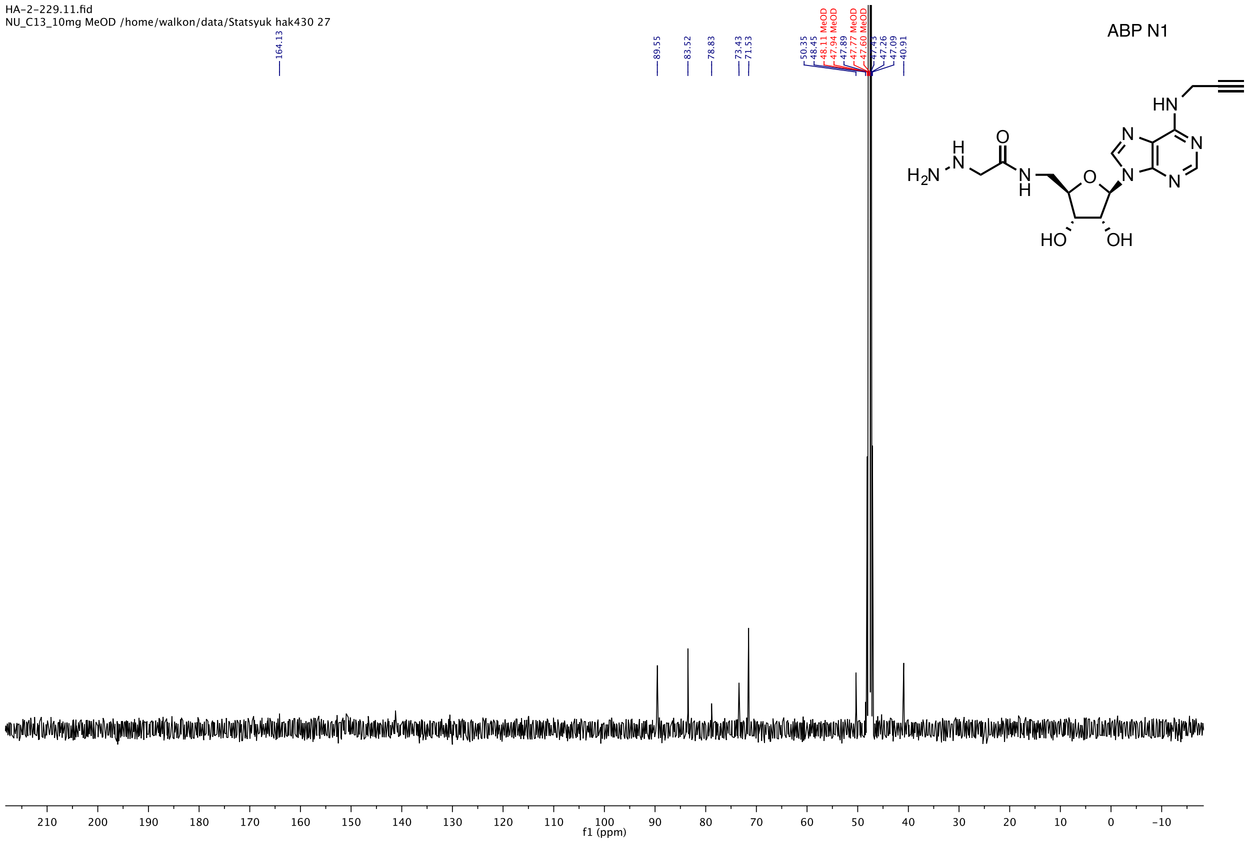
HA-2-229.11.fid
NU_C13_10mg MeOD /home/walkon/data/Statsyuk hak430 27
ABP N1
164.13
89.55
83.52
78.83
73.43
71.53
50.35
48.45
48.11 MeOD
47.94 MeOD
47.89
47.77 MeOD
47.60 MeOD
47.43
47.26
47.09
40.91
f1 (ppm)

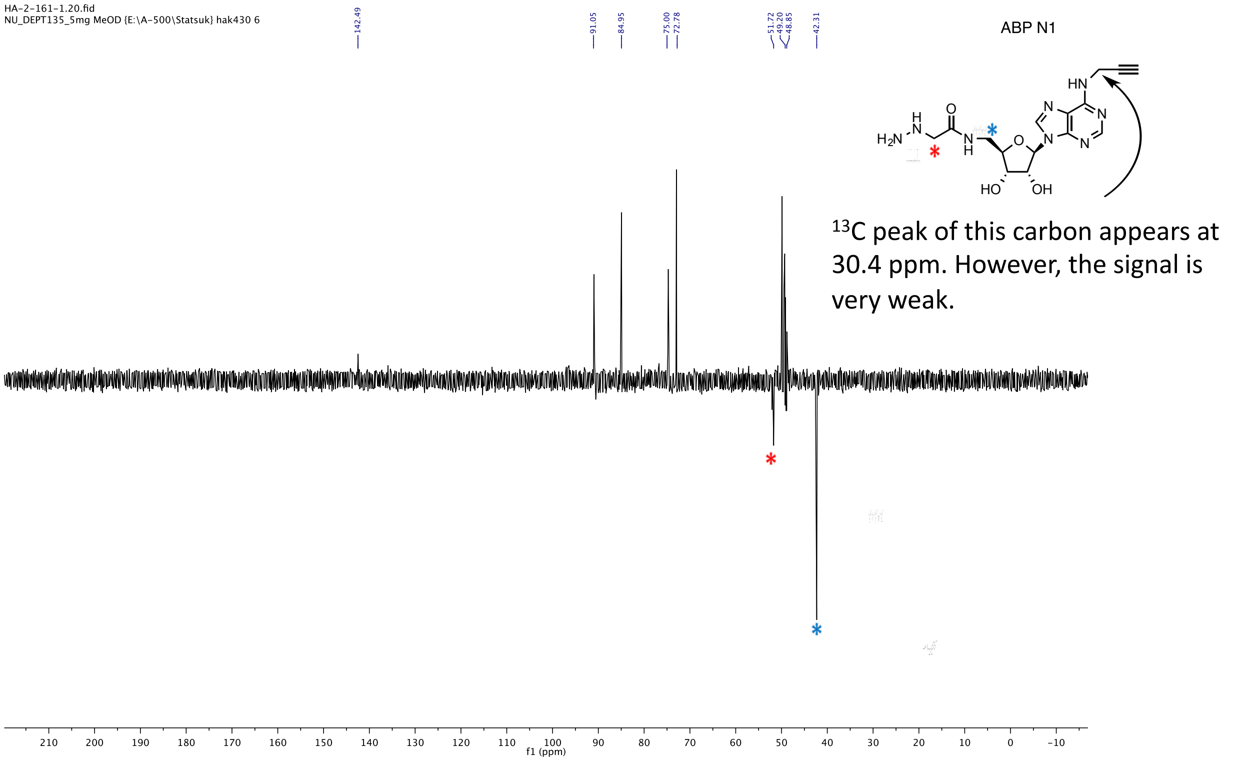
HA-2-161-1.20.fid
NU_DEPT135_5mg MeOD {E:\A-500\Statsuk} hak430 6
142.49
91.05
84.95
75.00
72.78
51.72
49.20
48.85
42.31
ABP N1
^{13}C peak of this carbon appears at 30.4 ppm. However, the signal is very weak.
f1 (ppm)

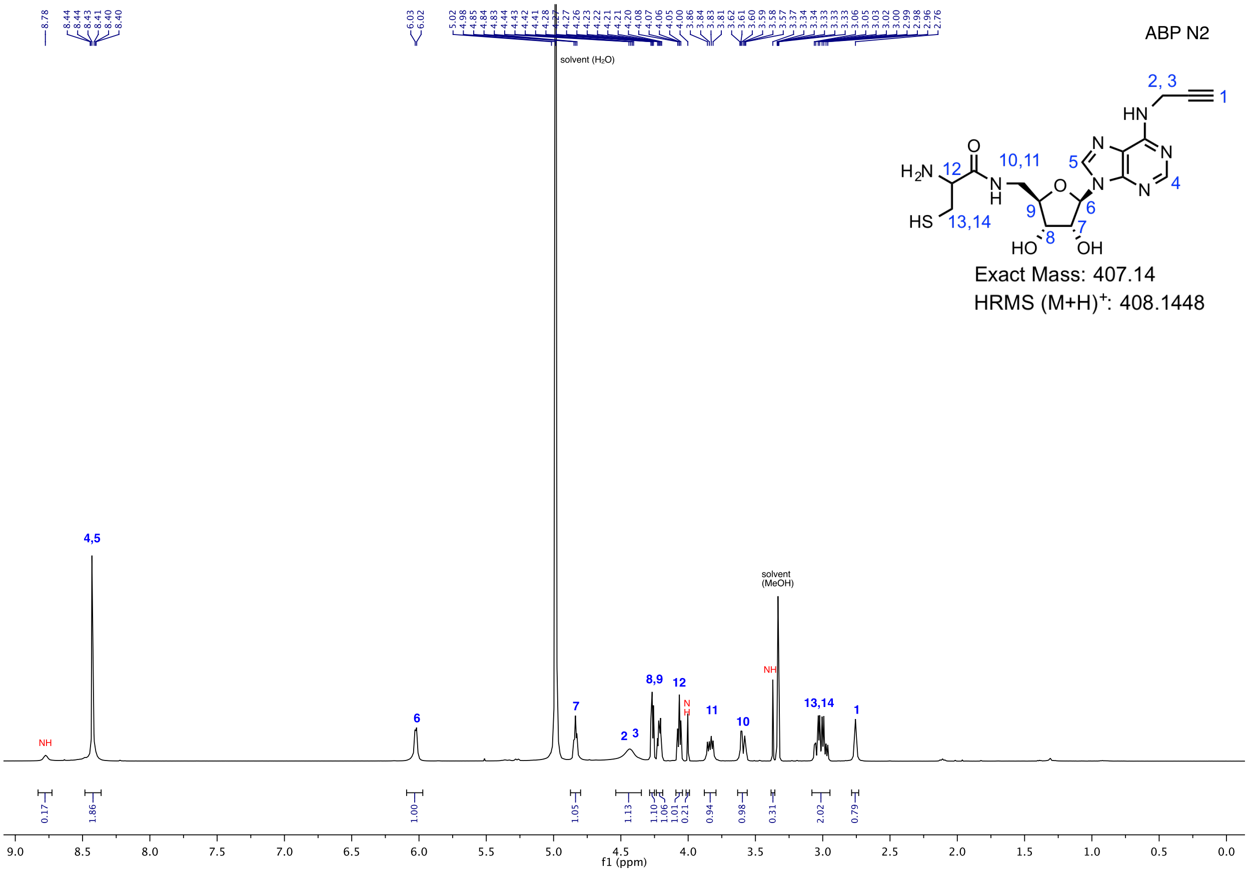
ABP N2
Exact Mass: 407.14
HRMS (M+H)$^{+}$: 408.1448
solvent (H₂O)
solvent (MeOH)
NH
4,5
6
7
2 3
8,9
12
11
10
13,14
1
f1 (ppm)

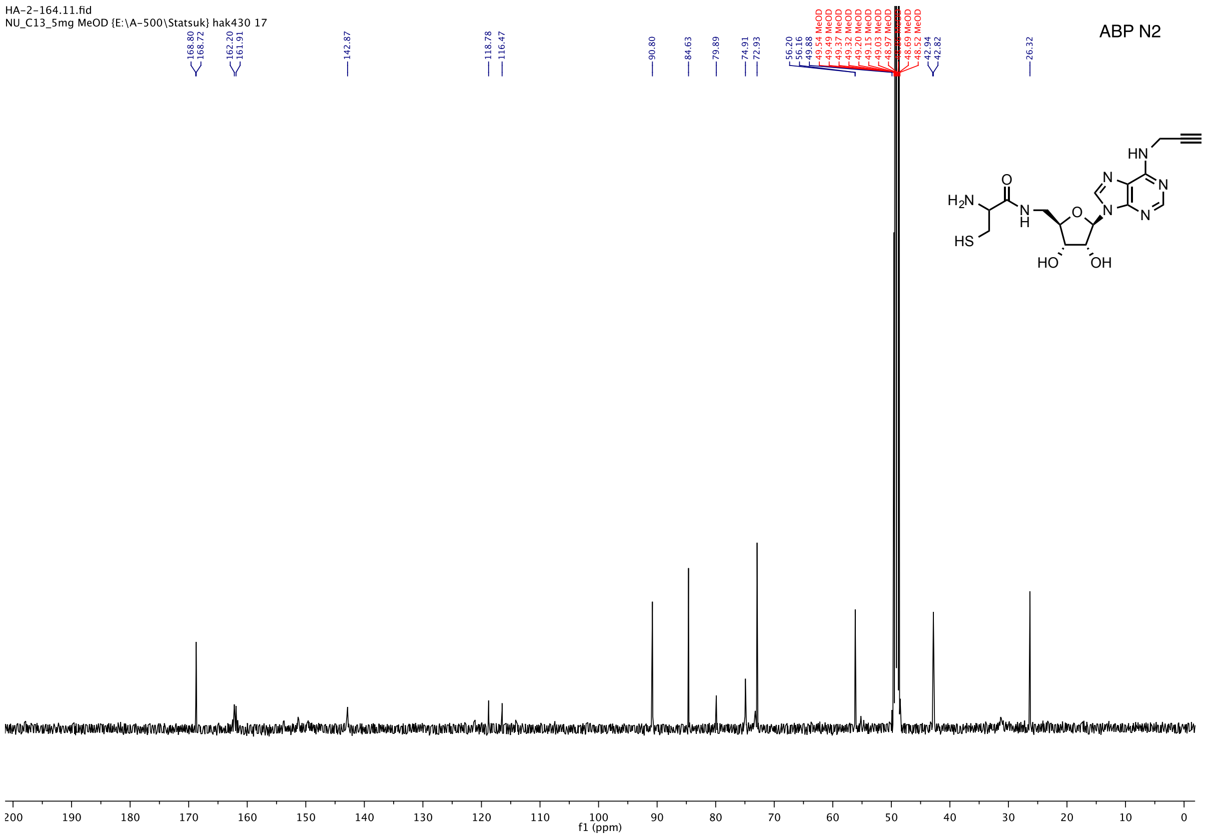
HA-2-164.11.fid
NU_C13_5mg MeOD {E:\A-500\Statsuk} hak430 17
ABP N2
168.80
168.72
162.20
161.91
142.87
118.78
116.47
90.80
84.63
79.89
74.91
72.93
56.20
56.16
49.88
42.94
42.82
26.32
f1 (ppm)

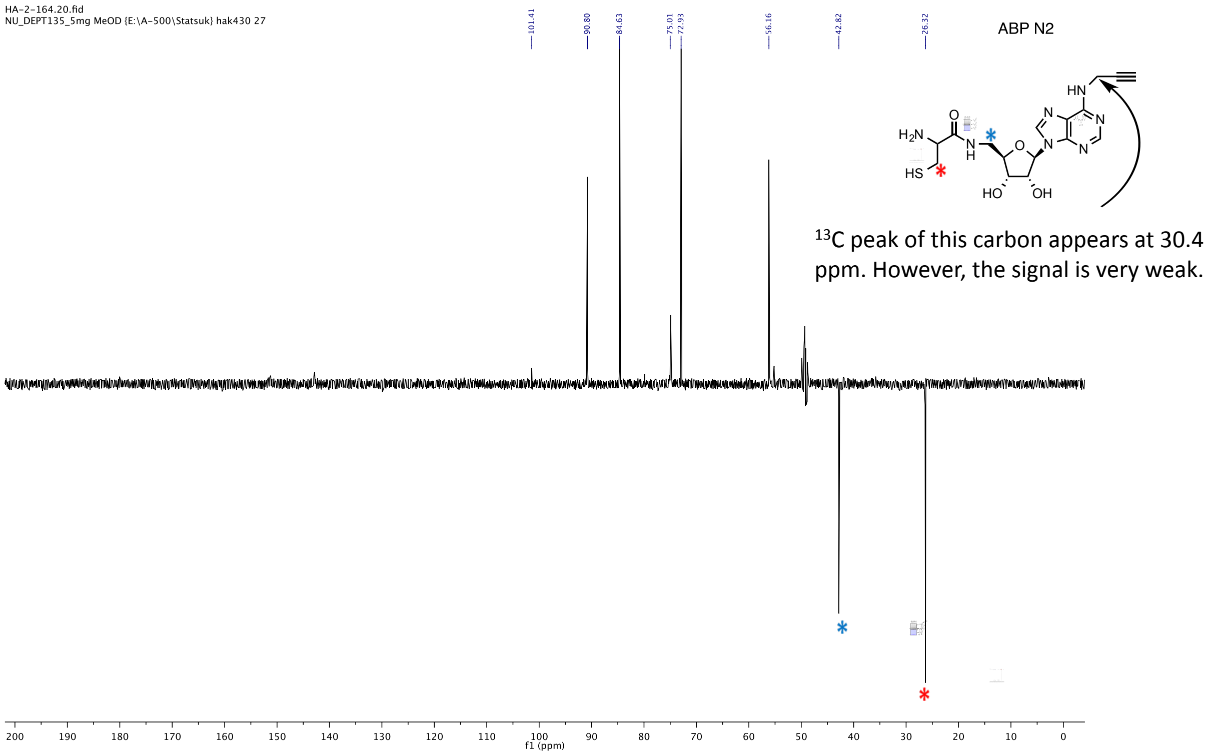
HA-2-164.20.fid
NU_DEPT135_5mg MeOD {E:\A-500\Statsuk} hak430 27
ABP N2
101.41
90.80
84.63
75.01
72.93
56.16
42.82
26.32
^{13}C peak of this carbon appears at 30.4 ppm. However, the signal is very weak.
f1 (ppm)

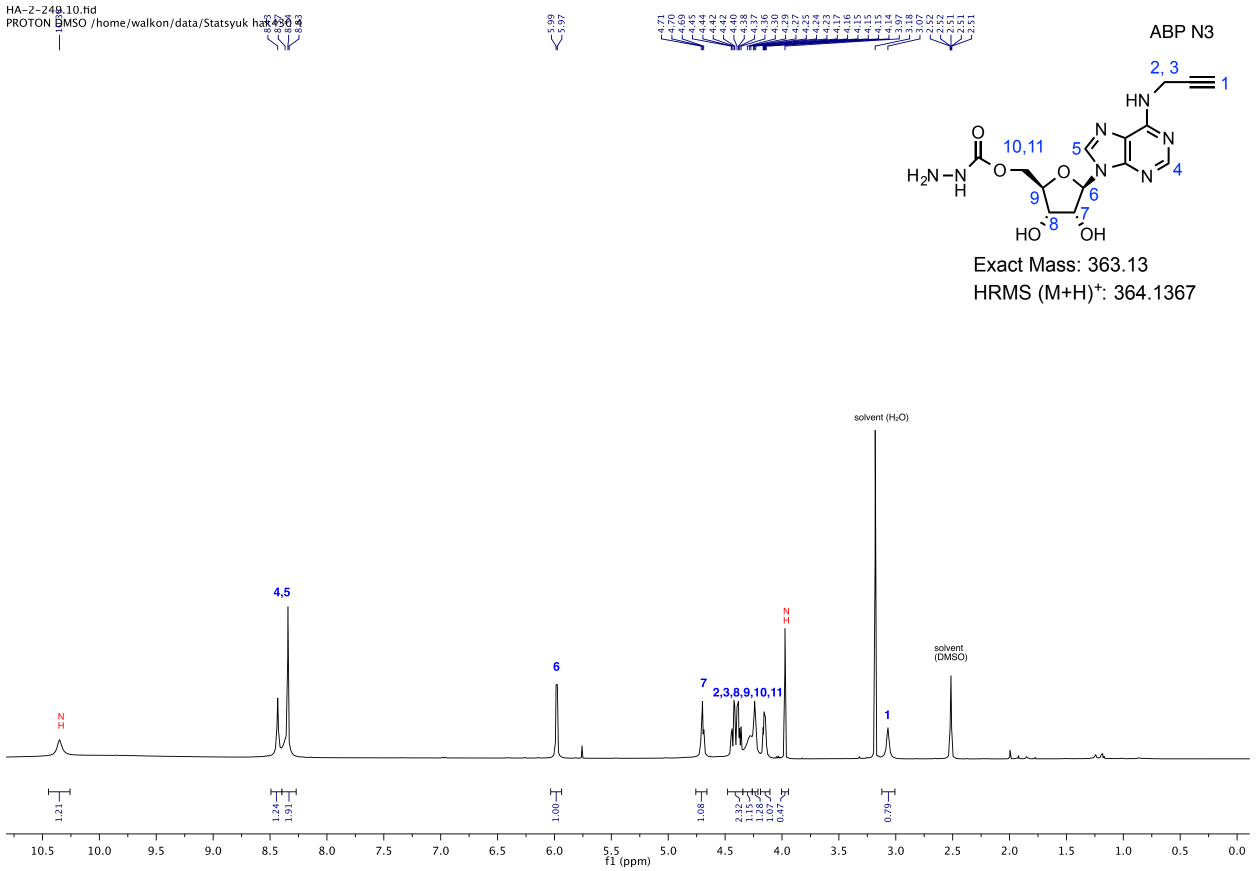
HA-2-249.10.fid
PROTON DMSO /home/walkon/data/Statsyuk hak430 4
ABP N3
Exact Mass: 363.13
HRMS (M+H)+: 364.1367
solvent (H2O)
solvent (DMSO)
4,5
6
7
2,3,8,9,10,11
1
f1 (ppm)

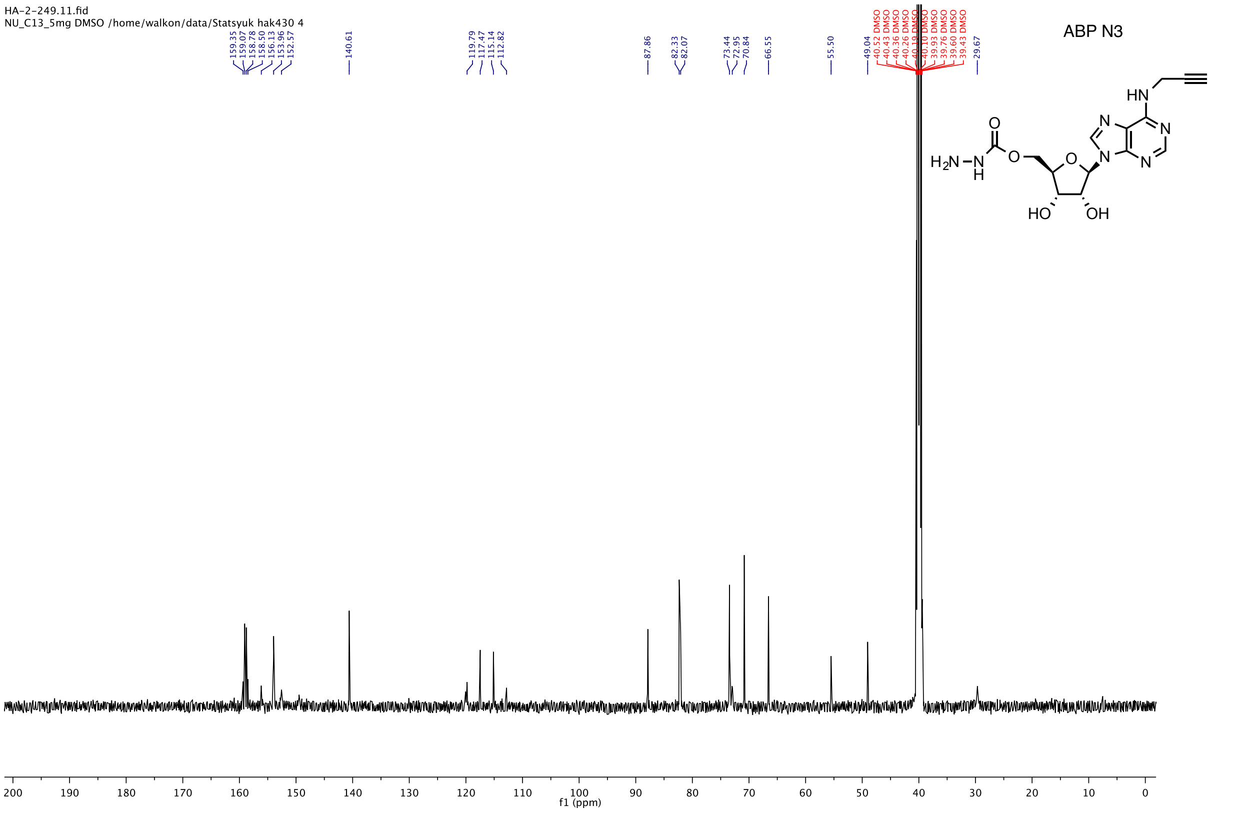
HA-2-249.11.fid
NU_C13_5mg DMSO /home/walkon/data/Statsyuk hak430 4
ABP N3
140.61
87.86
66.55
55.50
49.04
29.67
f1 (ppm)

ABP N4

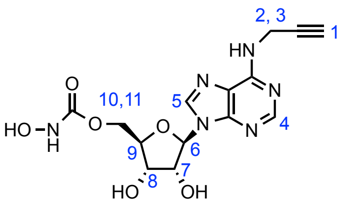

Exact Mass: 364.11

HRMS (M+H)$^+$: 365.1199

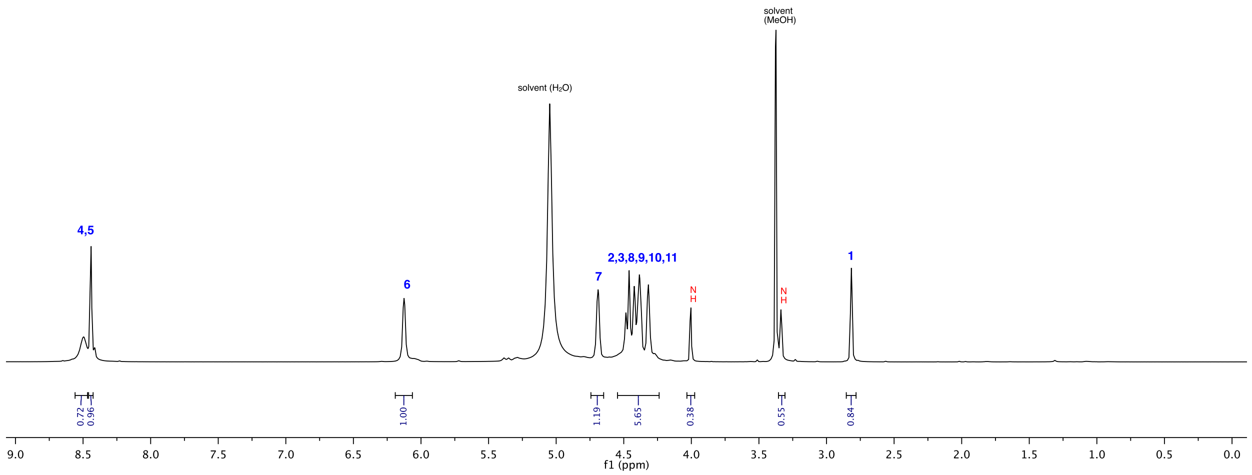

ABP N4

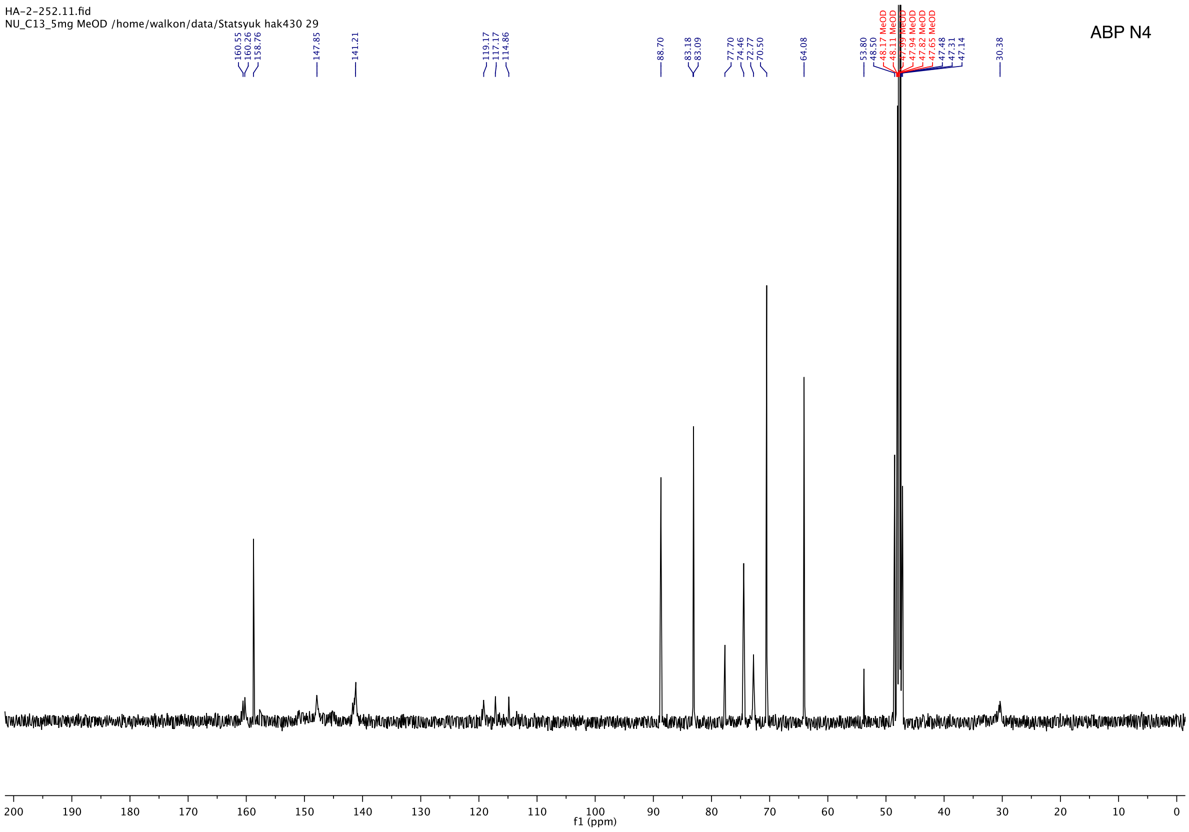

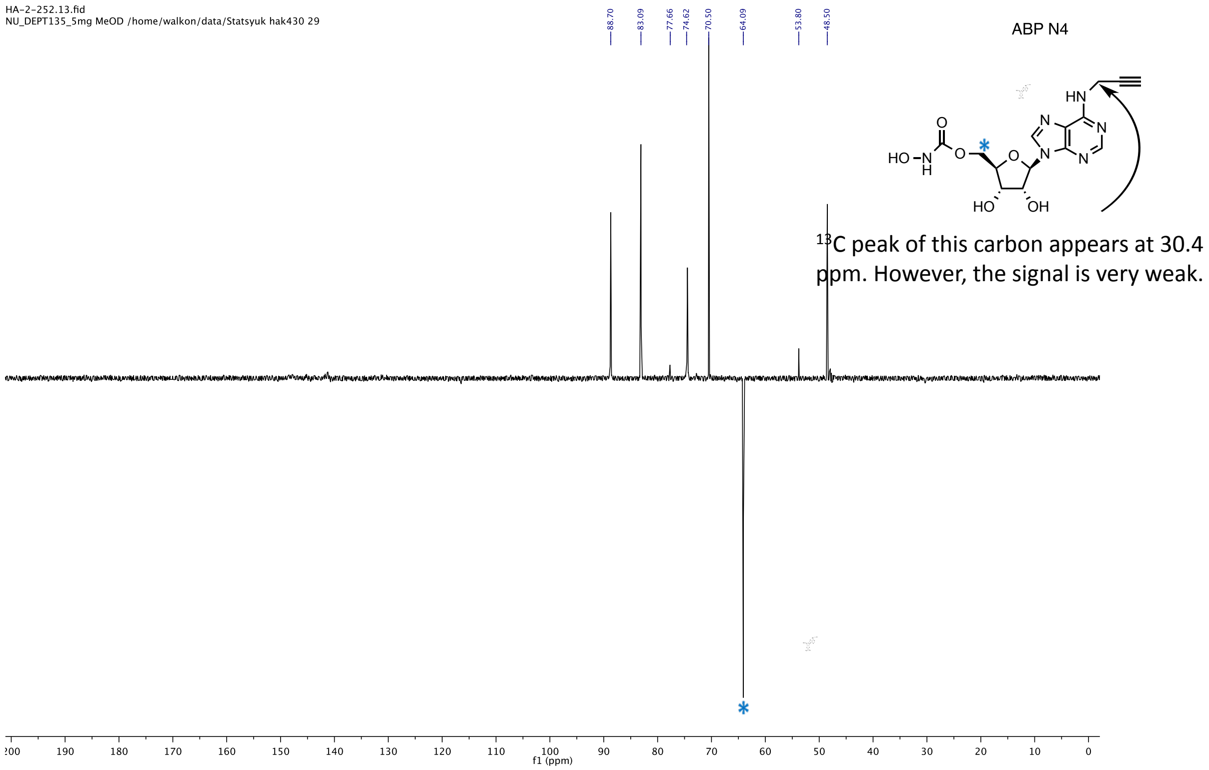

^{13}C peak of this carbon appears at 30.4 ppm. However, the signal is very weak.

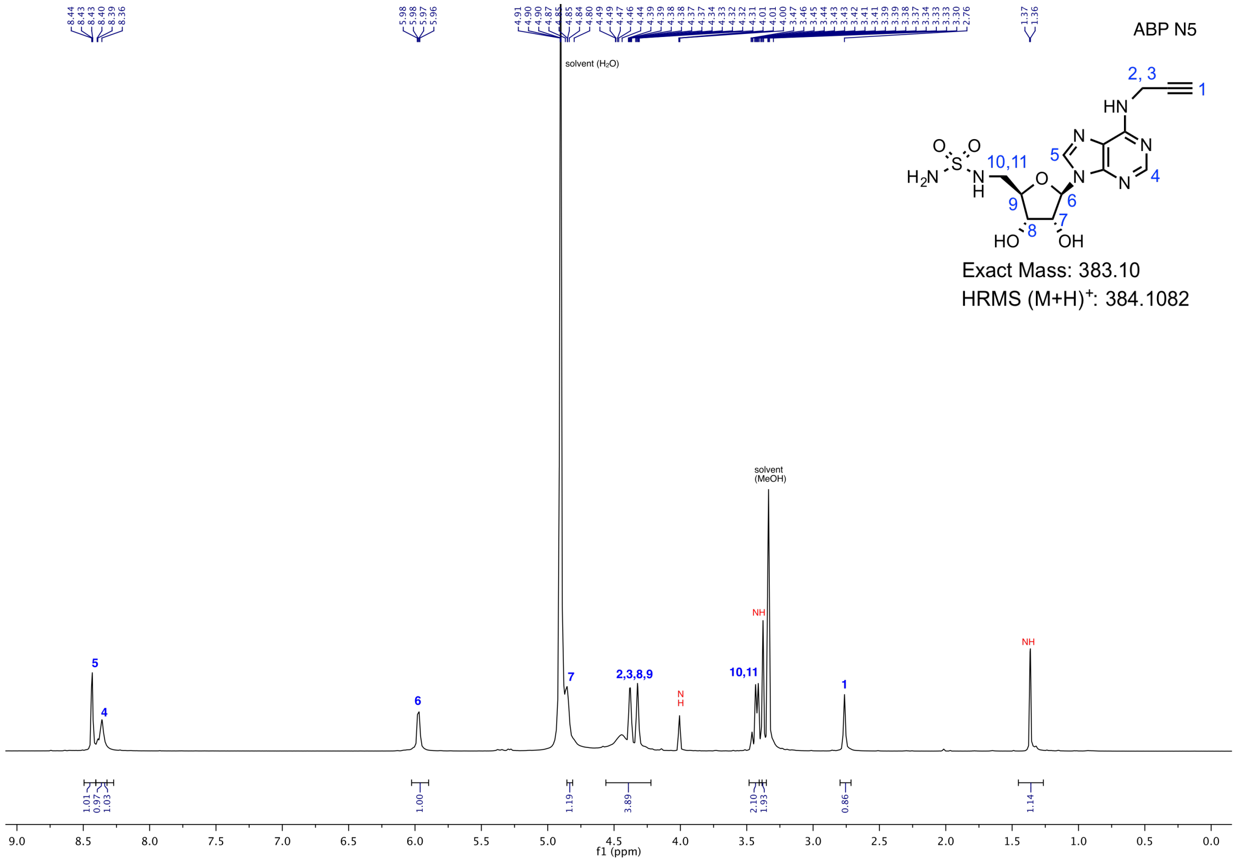

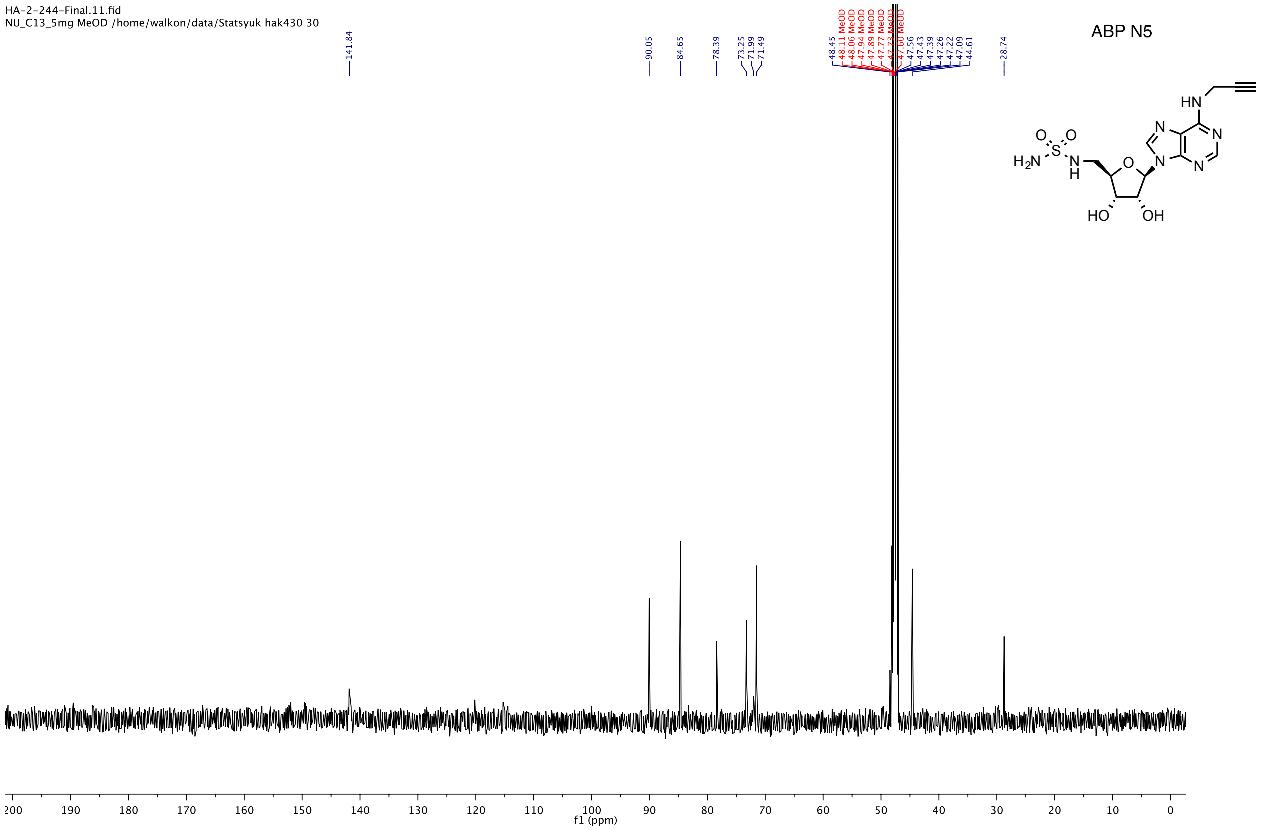

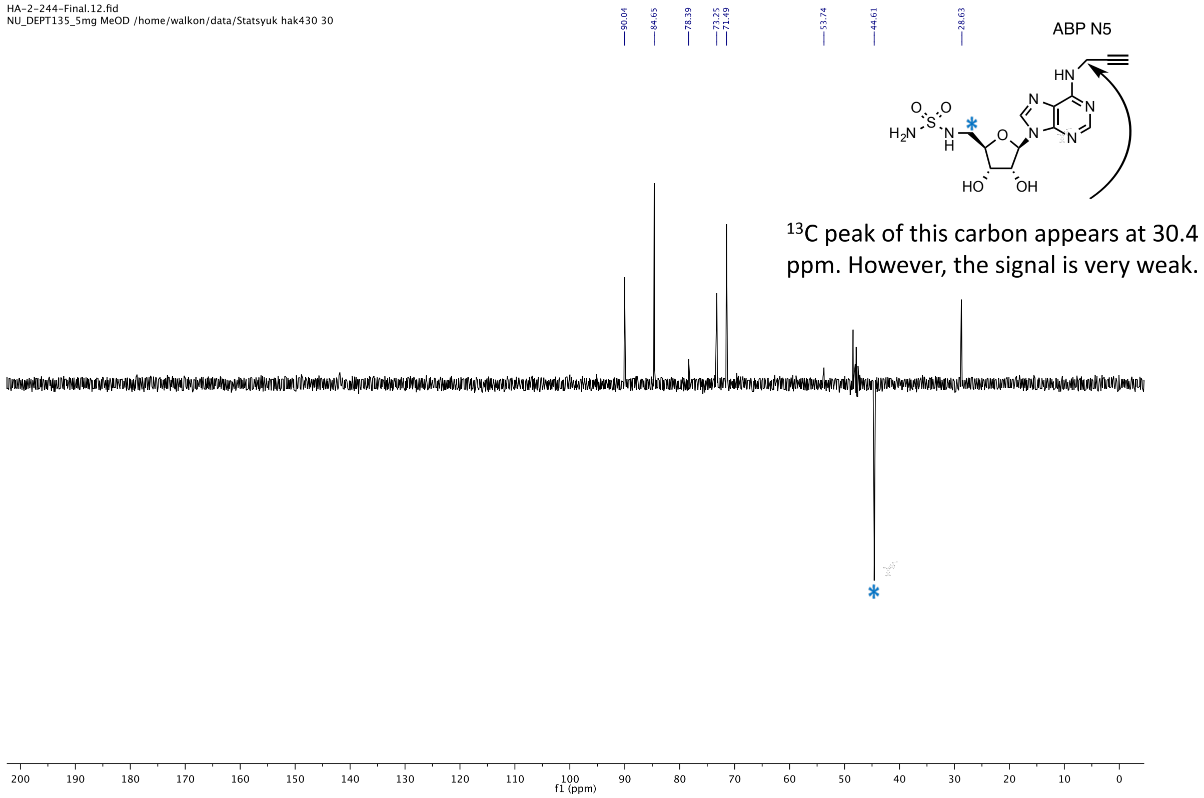

^{13}C peak of this carbon appears at 30.4 ppm. However, the signal is very weak.

(1) Engelhart, C. A.; Aldrich, C. C. *The Journal of organic chemistry* **2013**, *78*, 7470.
